# Supplementary material for: The GATAD2B-NuRD complex drives DNA:RNA hybrid-dependent chromatin boundary formation upon DNA damage
Source: EMBO J. 2024 May 8;43(12):8. doi: 10.1038/s44318-024-00111-7 (PMC11183058; doi:10.1038/s44318-024-00111-7)
Supplement: Supplementary file 1 — Appendix [file 44318_2024_111_MOESM1_ESM.pdf]

The GATAD2B-NuRD complex drives DNA:RNA hybrid-dependent chromatin boundary formation upon DNA damage

Zhichao Liu et al

Table of Contents:

Appendix Figure S1
Appendix Figure S2
Appendix Figure S3
Appendix Figure S4
Appendix Figure S5
Appendix Figure S6
Appendix Figure S7
Appendix Figure S8

Appendix Figure S1

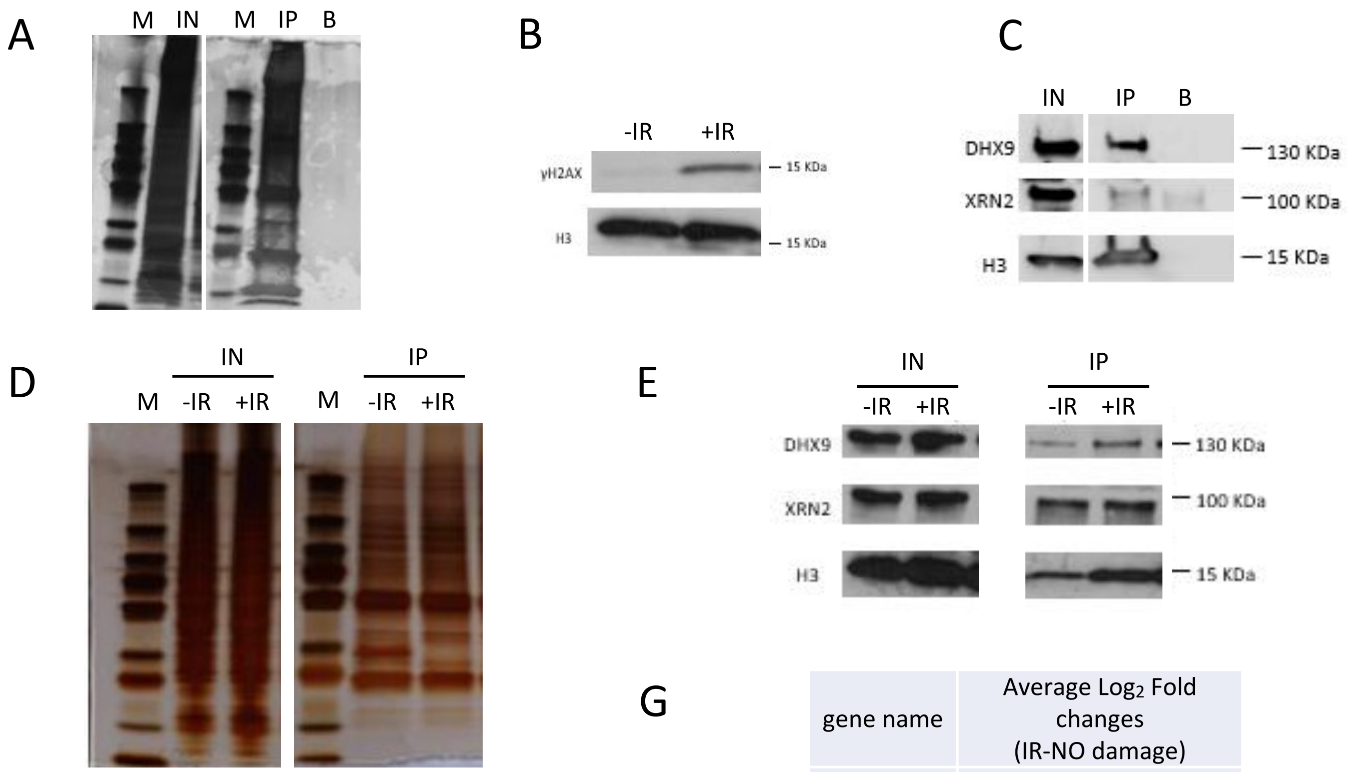

F

Name	Intensity
DDX5	6.12E+10
DDX21	5.81E+10
RBM14	3.07E+10
SMARCA5	1.81E+10
XRN2	1.14E+10
PARP1	1.12E+10
ILF3	6.14E+09
CHD4	4.84E+09
RBMX;RBMXL1	1.97E+09
TOP1	1.55E+09
SRSF7	1.22E+09
HDAC1	8.96E+08
SRPK1	8.21E+08
WHSC1	6.26E+08
RAD50	4.94E+08
DNMT1	2.8E+08

G

gene name	Average Log_2 Fold changes (IR-NO damage)
IGFN1	10.75378799
TRIM4	7.780150414
PUF60	4.597714424
NOP10	3.916346073
HNRNPL	3.782220364
RUVBL2	3.518584728
RAN	3.48355341
SAFB2	3.428174496
ZNF189	3.157113552
LUC7L3	2.918427467
CRNKL1	2.841762066
BYSL	2.800688267
PCMT1	2.786775112
RBMX	2.751998425
HNRNPDL	2.715929508
PRPF31	2.357191563
PDS5B	2.226240635
SF3A3	2.145429134
MBD3	2.127829075
LMNB2	2.095678329
GATAD2B	1.500137329
HNRNPR	1.27636528
LARP7	1.211964607
PWP2	1.142299652

Appendix Figure S1: A) Silver-stained gel showing proteins in S9.6 IP and no antibody (B) control. B) Western blot analysis of γH2AX showing that DSBs were successfully induced by 10 Gy IR. C) Western blot analysis showing presence of XRN2, DHX9 and H3, in S9.6 IP and control (B) samples. D) Silver-stained gel showing proteins in S9.6 IP samples prepared for mass spectroscopy analysis. E) Western blot analysis showing presence of XRN2, DHX9 and H3 in S9.6 IP samples prepared for mass spectroscopy analysis. F) Table showing known R-loop binding factors which have been verified by mass spectroscopy in S9.6 IP in no damage condition and their intensities. G) Table showing proteins which have been identified by mass spectroscopy to preferentially interact with R-loops upon IR treatment.

Appendix Figure S2

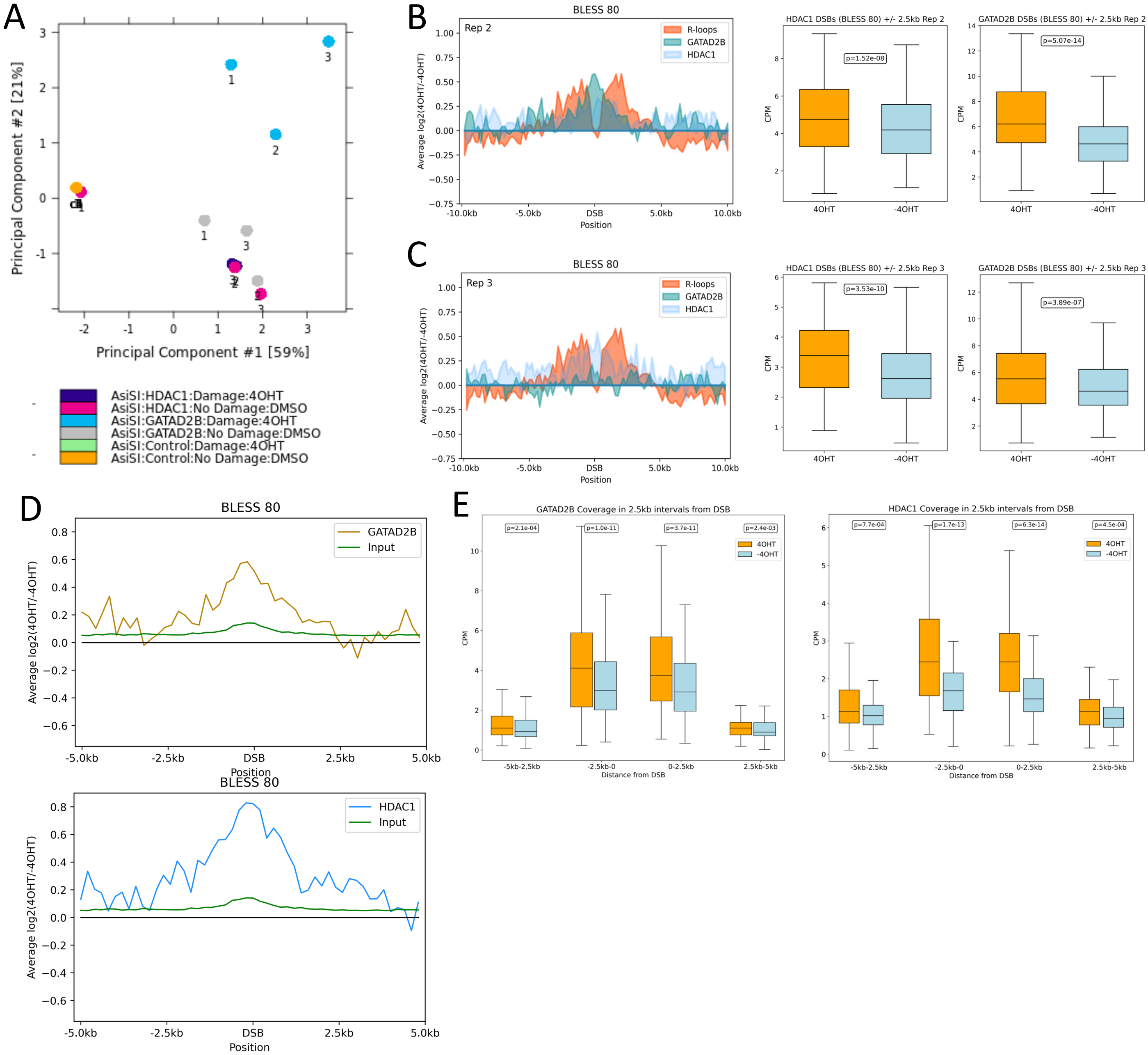

Appendix Figure S2: A) PCA plots showing input (control), GATAD2B and HDAC1 ChIP-seq replicates from 4 hr 4OHT-treated and untreated DIvA cells comparing consensus peaks detected using MACS2. HDAC1: Damage replicates are all clustered together appearing as a single black point. The Control: No Damage i,e Input samples from no damage, are also clustered together appearing as a single orange point. One of the HDAC1:No Damage clustered with the Control: No Damage samples, see one orange and pink points close together. B) Right: GATAD2B and HDAC1 ChIP-seq metagene profiles showing average log2(4OHT/-40HT) from biological replicate 2. Left: Box plots comparing ChIP Seq read coverage of HDAC1 and GATAD2B in 2.5kb flanking region of BLESS 80 sites between damage (+4OHT) and no damage conditions in replicate 2. Wilcoxon 2 sample test is used for statistically testing of medians between coverages in control and condition. C) Right: GATAD2B and HDAC1 ChIP-seq metagene profiles showing average log2(4OHT/-40HT) from biological replicate 3. Left: Box plots comparing ChIP Seq read coverage of HDAC1 and GATAD2B in 2.5kb flanking region of BLESS 80 sites between damage (+4OHT) and no damage conditions in replicate 3. Wilcoxon 2 sample test is used for statistically testing of medians between coverages in control and condition. D) Metagene profile showing log_2fold (+4OHT/-4OHT) ChIP-seq enrichment of GATAD2B, HDAC1 and input at AsiSI cut sites (as defined by BLESS technique). E) GATAD2B and HDAC1ChIP-Seq coverage in 2.5kb intervals from BLESS80 sites in both damage(+4OHT) and no damage conditions. Wilcoxon 2 sample test is used for statistically testing of medians between coverages in control and condition.

Appendix Figure S3

A

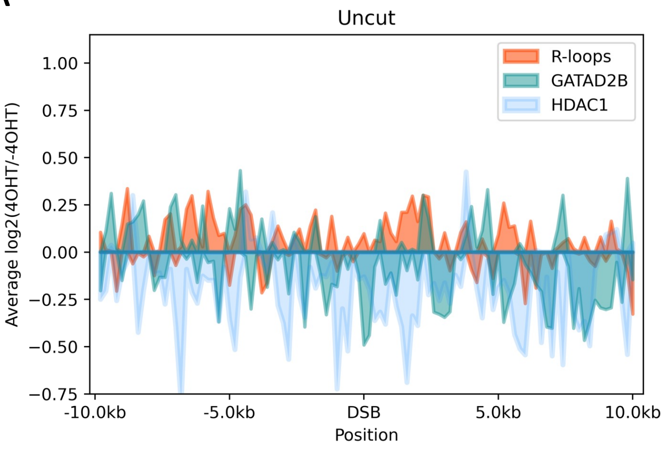

B

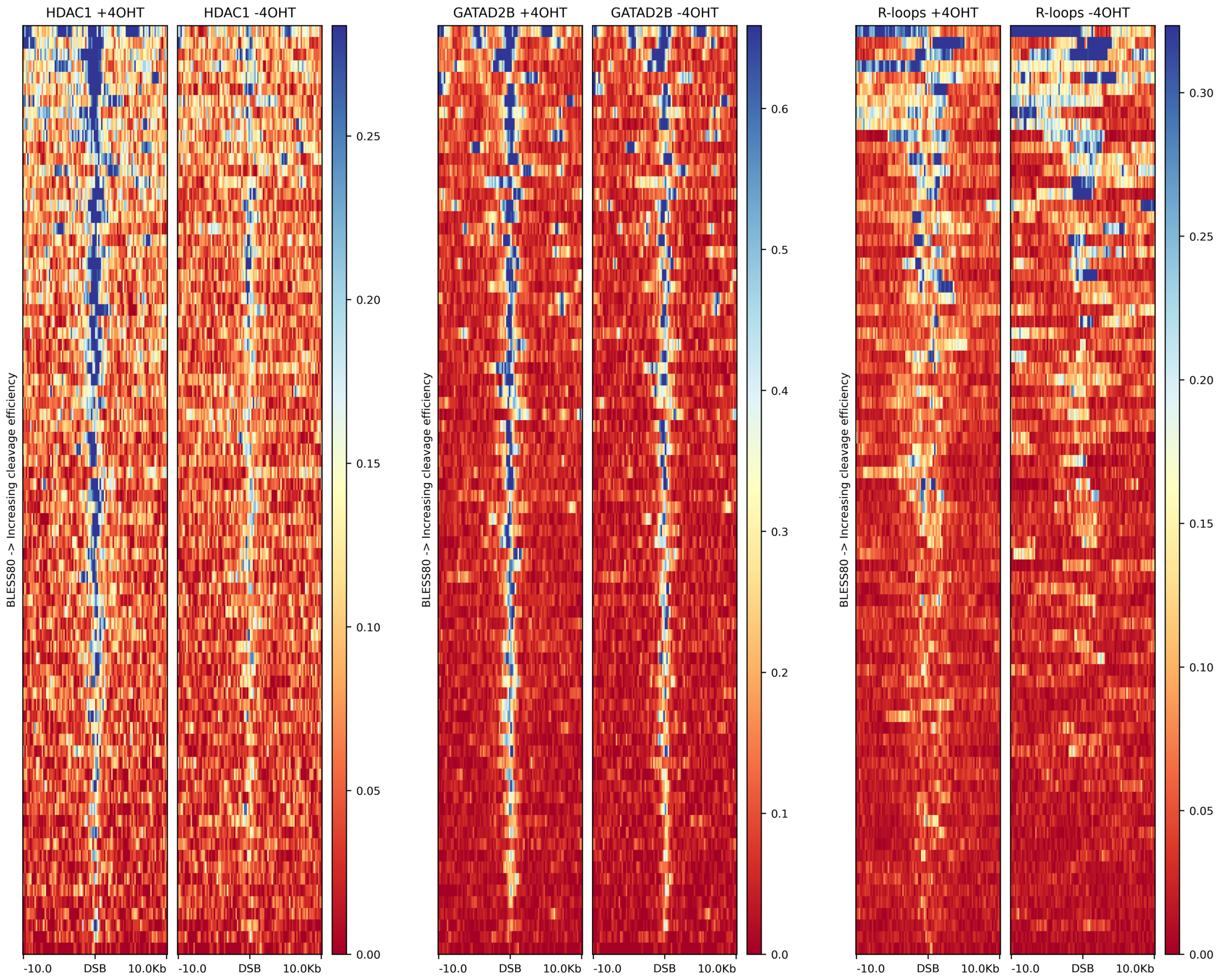

Appendix Figure S3: A) Metagene profile showing ChIP-seq enrichment of GATAD2B, HDAC1 and R-loops between 4 hr 4OHT-treated and untreated DIvA cells at AsiSI uncut sites (as defined by BLESS technique) over a 20 kb window. Values are presented as log_2 ratios. B) Heatmaps representing GATAD2B and HDAC1 ChIP-seq count over a 20 kb window centered on the DSB before(−4OHT) and after(+4OHT) DSB induction. DSBs are sorted according to decreasing cutting efficiency.

Appendix Figure S4

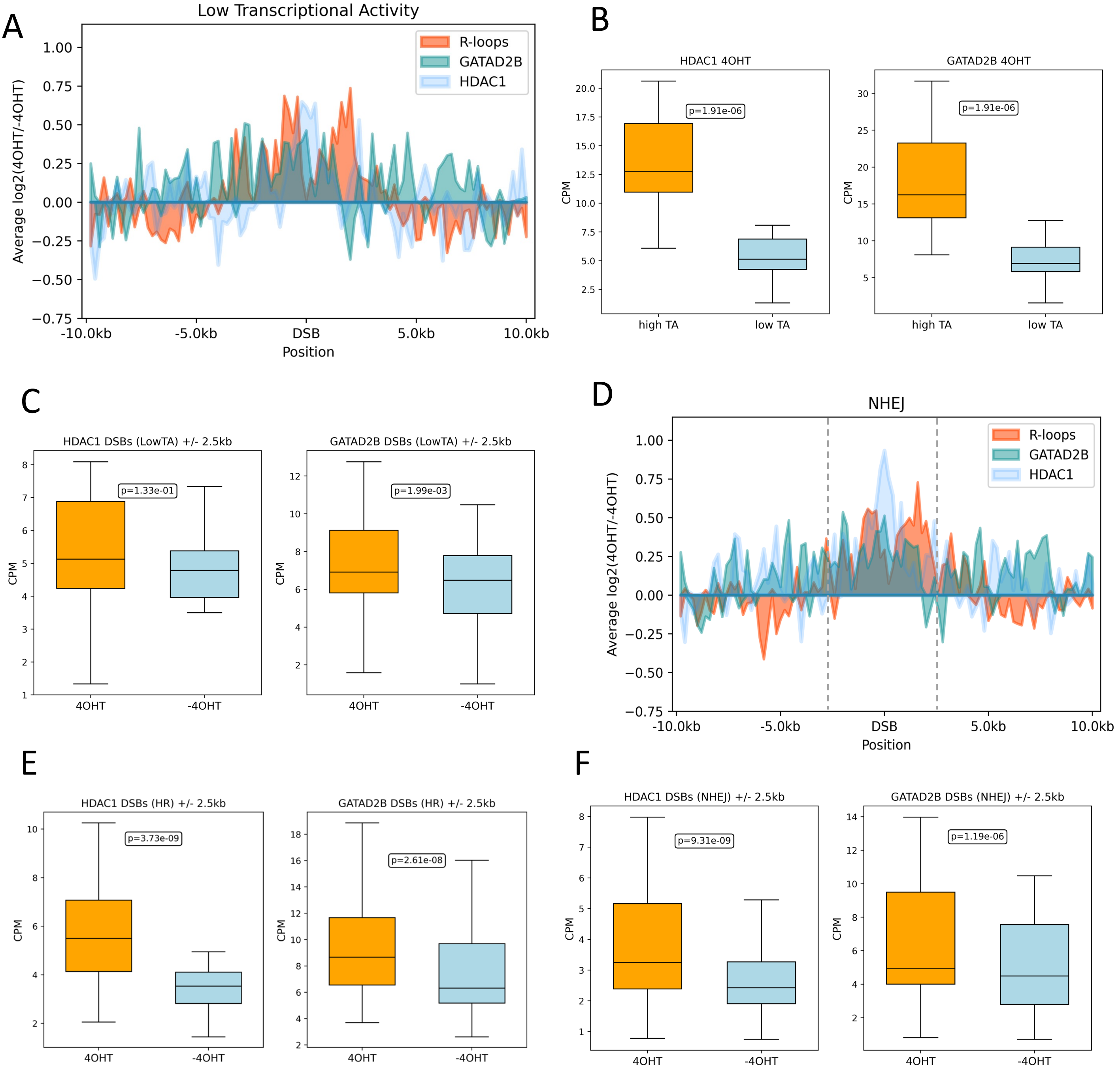

Appendix Figure S4: A) Metagene profile showing ChIP-seq enrichment of GATAD2B, HDAC1 and R-loops between 4 hr 4OHT-treated and untreated DIvA cells at AsiSI cut sites in low transcribed regions over a 20 kb window. Values are presented as log_2 ratios. B) Box plot graph showing levels of HDAC1 and GATAD2B ChIP-seq reads mapping to AsiSI sites in presence or absence of 4OHT at high transcription regions. C) Box plot graph showing levels of HDAC1 and GATAD2B ChIP-seq reads mapping to AsiSI sites in presence or absence of 4OHT at low transcription regions. D) Metagene profile showing ChIP-seq enrichment of GATAD2B, HDAC1 and R-loops between 4 hr 4OHT-treated and untreated DIvA cells at NHEJ prone AsiSI cut sites (as defined by BLESS technique) over a 20 kb window. Values are presented as log_2 ratios. E) Box plot graph showing levels of HDAC1 and GATAD2B ChIP-seq reads mapping to AsiSI sites in presence or absence of 4OHT at HR prone regions. F) Box plot graph showing levels of HDAC1 and GATAD2B ChIP-seq reads mapping to AsiSI sites in presence or absence of 4OHT at NHEJ prone regions.

Appendix Figure S5

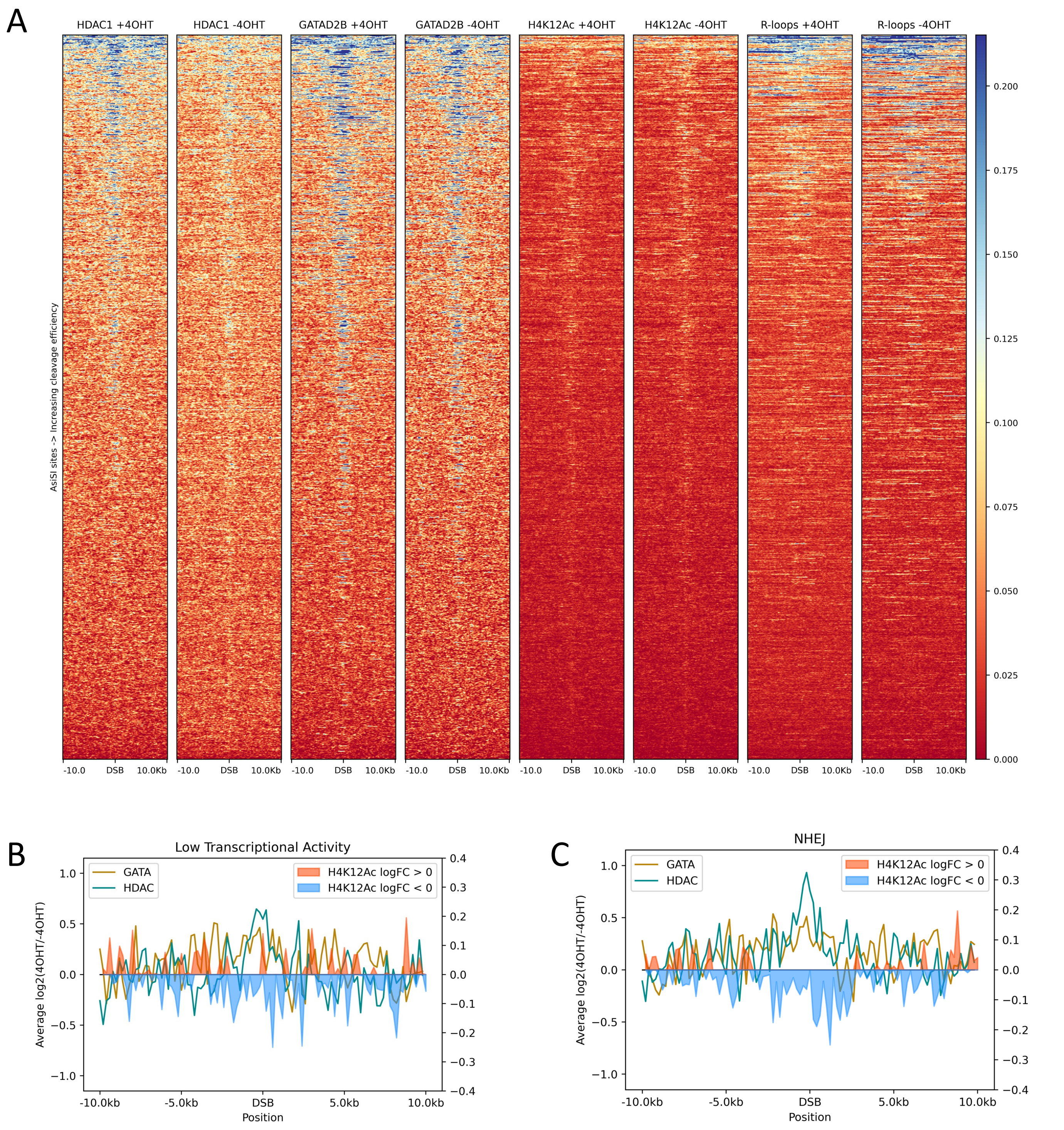

Appendix Figure S5: A) Heatmaps representing GATAD2B and HDAC1 ChIP-seq, DRIP-seq and H4K12Ac ChIP-seq count over a 20 kb window centered on the DSB before(−4OHT) and after(+4OHT) DSB induction. DSBs are sorted according to decreasing cutting efficiency. B) Metagene profile showing ChIP-seq enrichment of GATAD2B, HDAC1 and H4K12ac between 4 hr 4OHT-treated and untreated DIvA cells at AsiSI cut sites in low transcribed regions over a 20 kb window. Values are presented as log_2 ratios. C) Metagene profile showing ChIP-seq enrichment of GATAD2B, HDAC1 and H4K12ac between 4 hr 4OHT-treated and untreated DIvA cells at NHEJ prone AsiSI cut sites over a 20 kb window. Values are presented as log_2 ratios.

Appendix Figure S6

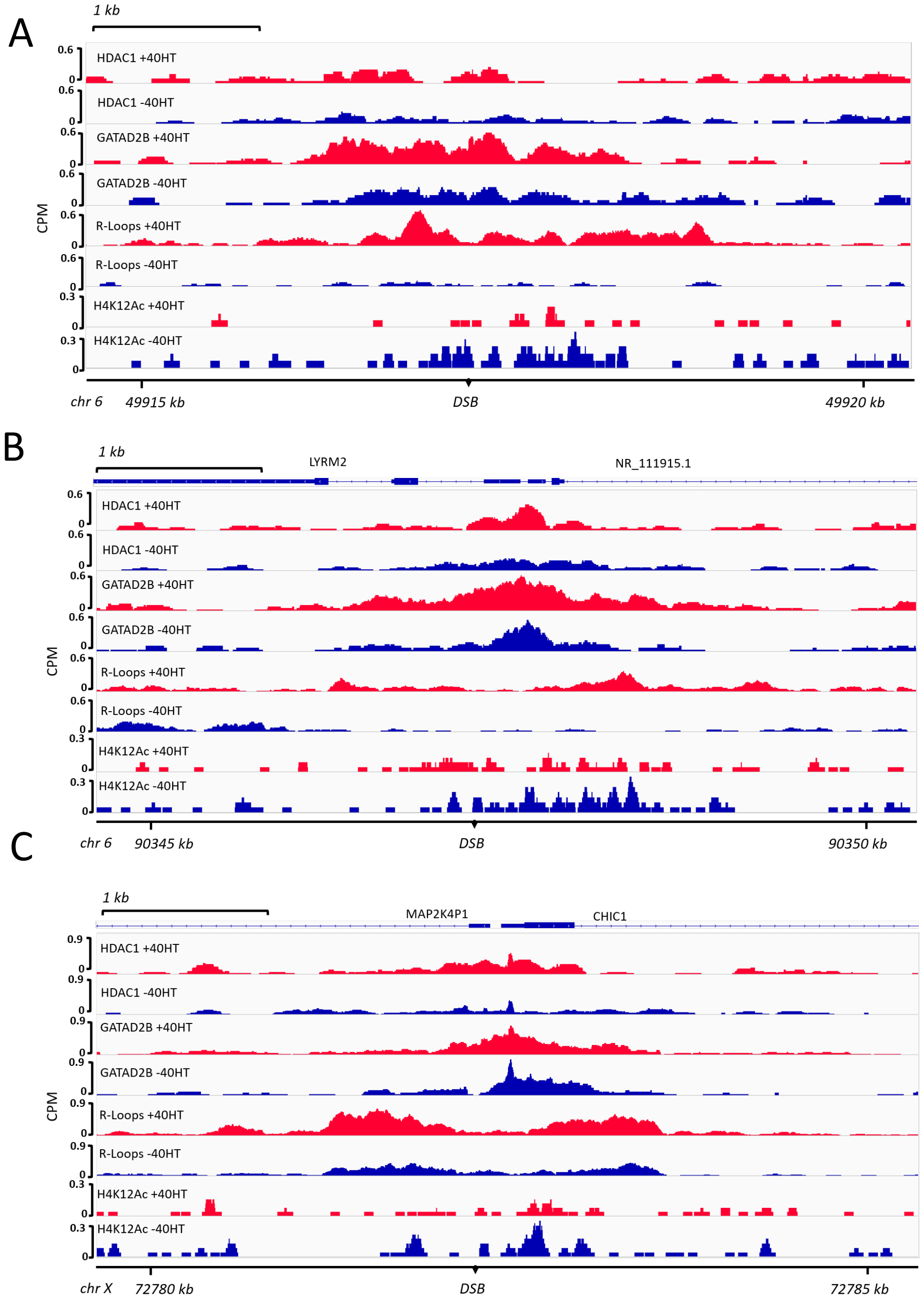

Appendix Figure S6 A-C) IGV screenshots showing HDAC1 and GATAD2B ChIP-Seq, DRIP-seq and H4K12Ac ChIP-seq reads count in no damage (-4OHT) and damage (+4OHT) conditions at representative AsiSI cutting sites.

Appendix Figure S7

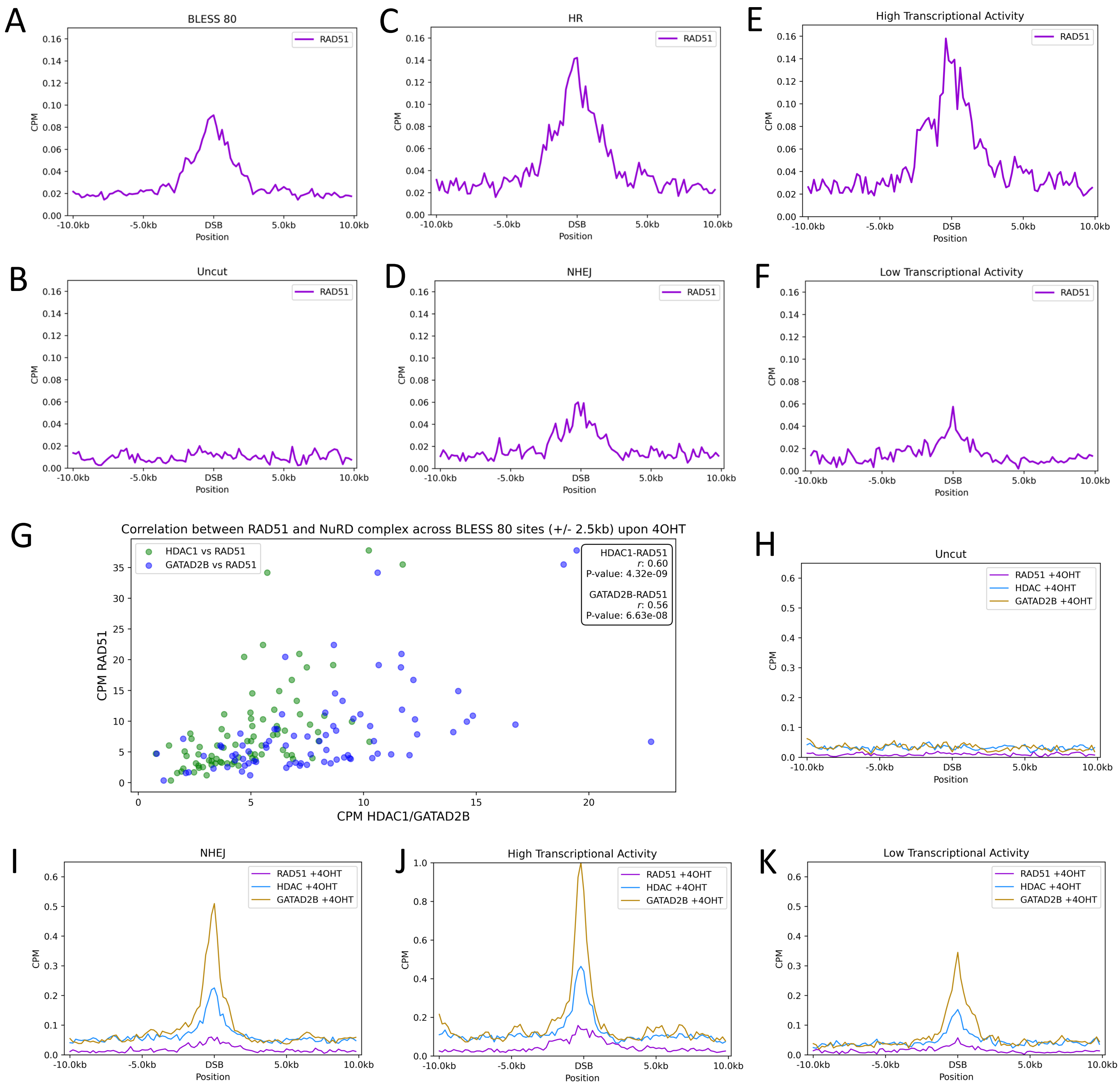

Appendix Figure S7: A) Metagene profile showing ChIP-seq enrichment of RAD51 at BLESS 80 AsiSI cut sites. B) Metagene profile showing ChIP-seq enrichment of RAD51 at uncut AsiSI sites. C) Metagene profile showing ChIP-seq enrichment of RAD51 at cut HR prone AsiSI sites. D) Metagene profile showing ChIP-seq enrichment of RAD51 at cut NHEJ prone AsiSI sites. E) Metagene profile showing ChIP-seq enrichment of RAD51 at highly transcribed cut AsiSI sites. F) Metagene profile showing ChIP-seq enrichment of RAD51 at low transcribed cut AsiSI sites. G) Scatter plot of RAD51 coverage vs HDAC1/GATAD2B coverage in 2.5kb flanking region of BLESS 80 sites upon damage induction with tamoxifen. Pearson's correlation coefficient and p-value shows correlation between RAD51 and HDAC1/GATAD2B coverage in 2.5kb flanking region of BLESS 80 sites upon tamoxifen treatment. H) Metagene profile showing ChIP-seq enrichment of GATAD2B, HDAC1 and RAD51 at uncut AsiSI sites. I) Metagene profile showing ChIP-seq enrichment of GATAD2B, HDAC1 and RAD51 at NHEJ prone cut AsiSI sites. J) Metagene profile showing ChIP-seq enrichment of GATAD2B, HDAC1 and RAD51 at highly transcribed cut AsiSI sites. K) Metagene profile showing ChIP-seq enrichment of GATAD2B, HDAC1 and RAD51 at low transcribed cut AsiSI sites.

Appendix Figure S8

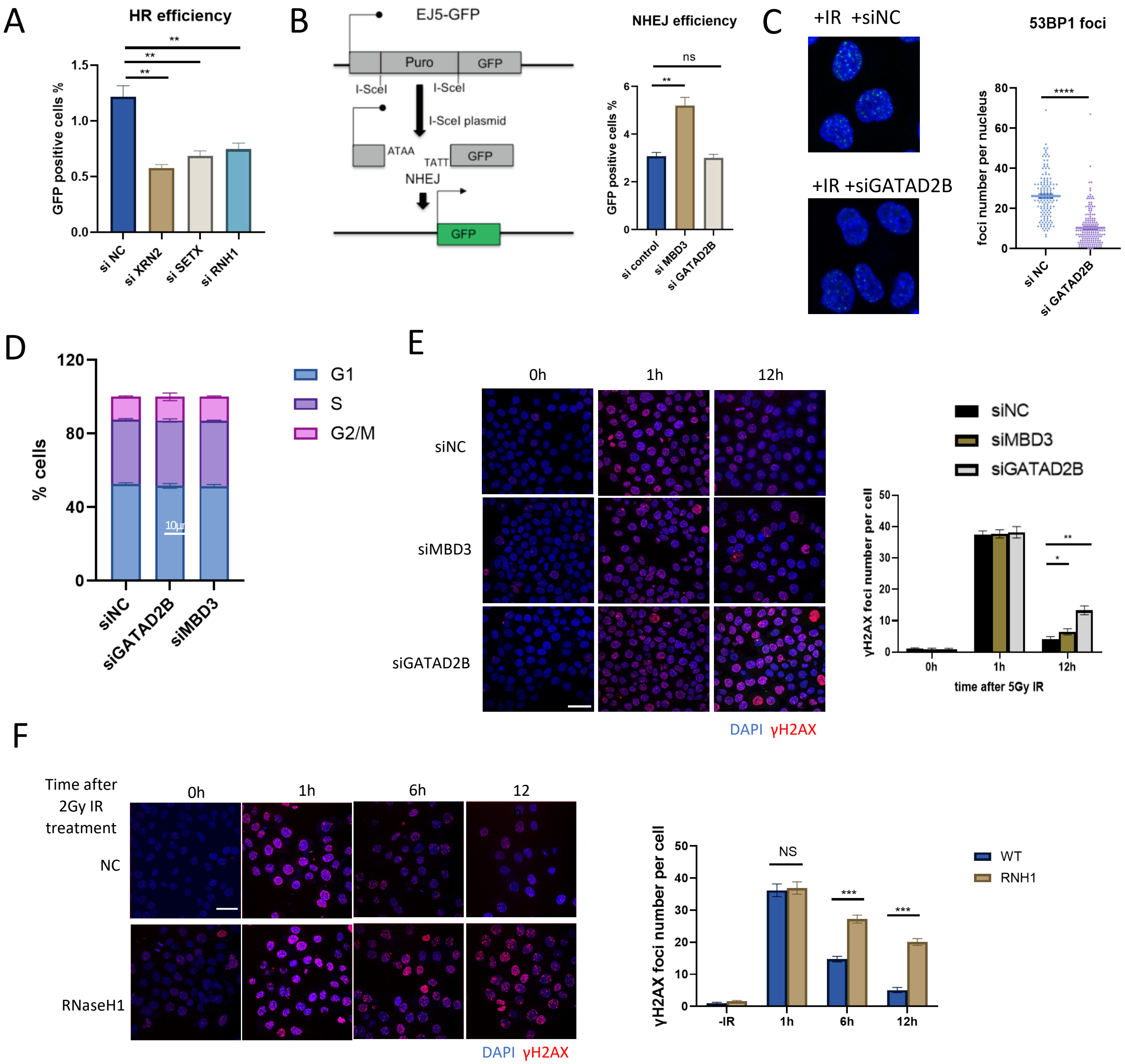

Appendix Figure S8: A) Bar charts showing HR repair efficiency under DSB inducted by I-SceI in U2OS cells containing HR reporter, and control (siNC) or knock down of XRN2, SETX or with overexpression of RNH1. error bar = mean ± SEM, significance was determined using non-parametric Mann-Whitney test. ** $p \le 0.01$. B) Left: drawing indicating structure of NHEJ reporter cassette. Right: Bar charts showing NHEJ repair efficiency under DSB inducted by I-SceI in U2OS cells containing NHEJ reporter, and knock down of MBD3 and GATAD2B C) Immunofluorescence of 53BP1 cells with 5 Gy IR followed by 20 min recovery, and depletion of GATAD2B. Left: representative confocal microscopy images; right: quantification of left, error bar = mean ± SEM, significance was determined using non-parametric Mann-Whitney test. ****$p \le 0.0001$. D) Percentage of cells in each stage of cell cycle with 5 Gy IR followed by 2hr recovery, and depletion of GATAD2B and MBD3, analyzed by flow cytometry. Data are presented as mean ± SEM, n=3. E) Immunofluorescence of γH2AX in cells with 5 Gy IR followed by recovery as indicated and without IR, and depletion of GATAD2B and MBD3. Left: representative confocal microscopy images; right: quantification of left, error bar = mean ± SEM, significance was determined using non-parametric Mann-Whitney test. ***$p \le 0.001$, ** $p \le 0.01$, * $p \le 0.05$. Scale bar = 50 μm, n > 3 F) As Supplementary Fig 8.E, immunofluorescence of γH2AX in cells with IR, and overexpression of RNaseH. Scale bar = 50 μm, n > 3
