# Supplementary figures and images for: The GATAD2B-NuRD complex drives DNA:RNA hybrid-dependent chromatin boundary formation upon DNA damage
Source: EMBO J. 2024 May 8;43(12):8. doi: 10.1038/s44318-024-00111-7 (PMC11183058; doi:10.1038/s44318-024-00111-7)

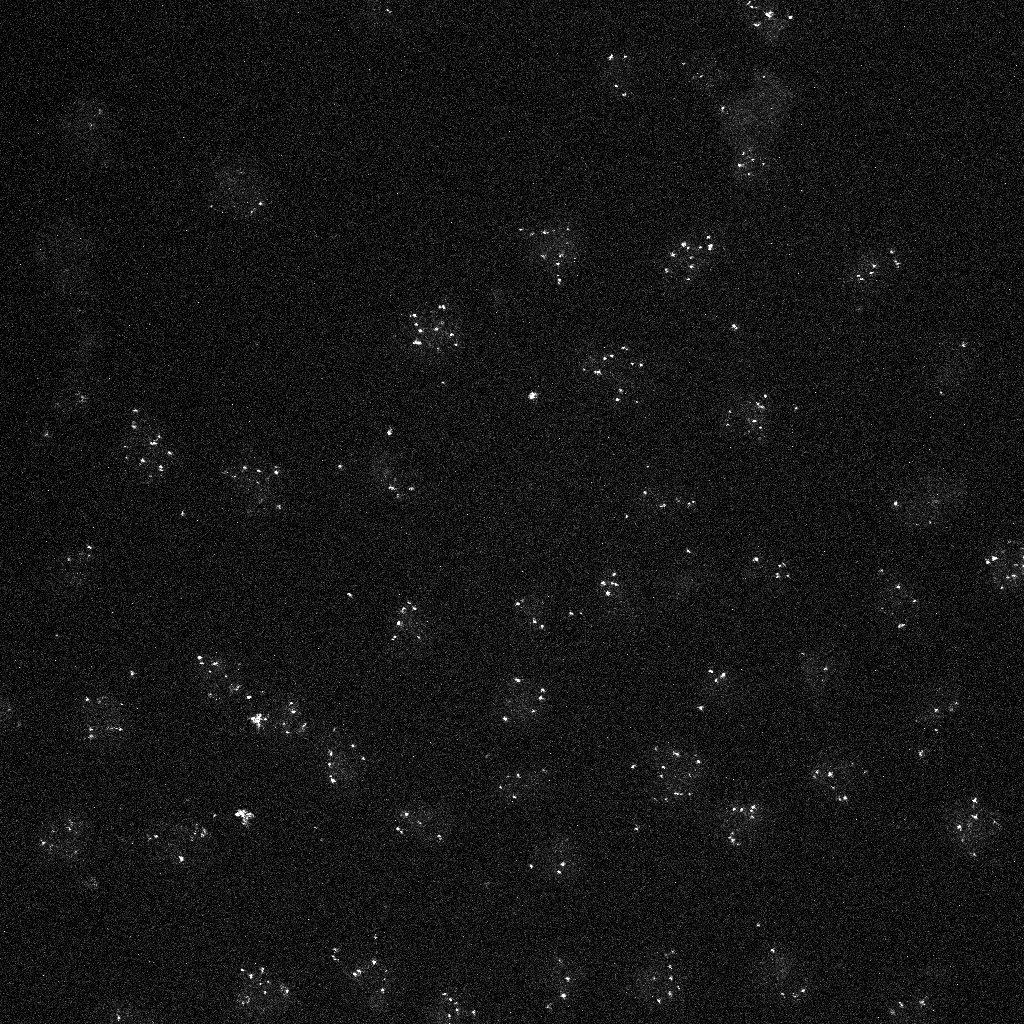

Supplement: Supplementary file 2 — Source data Fig. 1 [file 44318_2024_111_MOESM2_ESM.zip › Figure 1/Figure 1F/+IR +RNH1.tif]

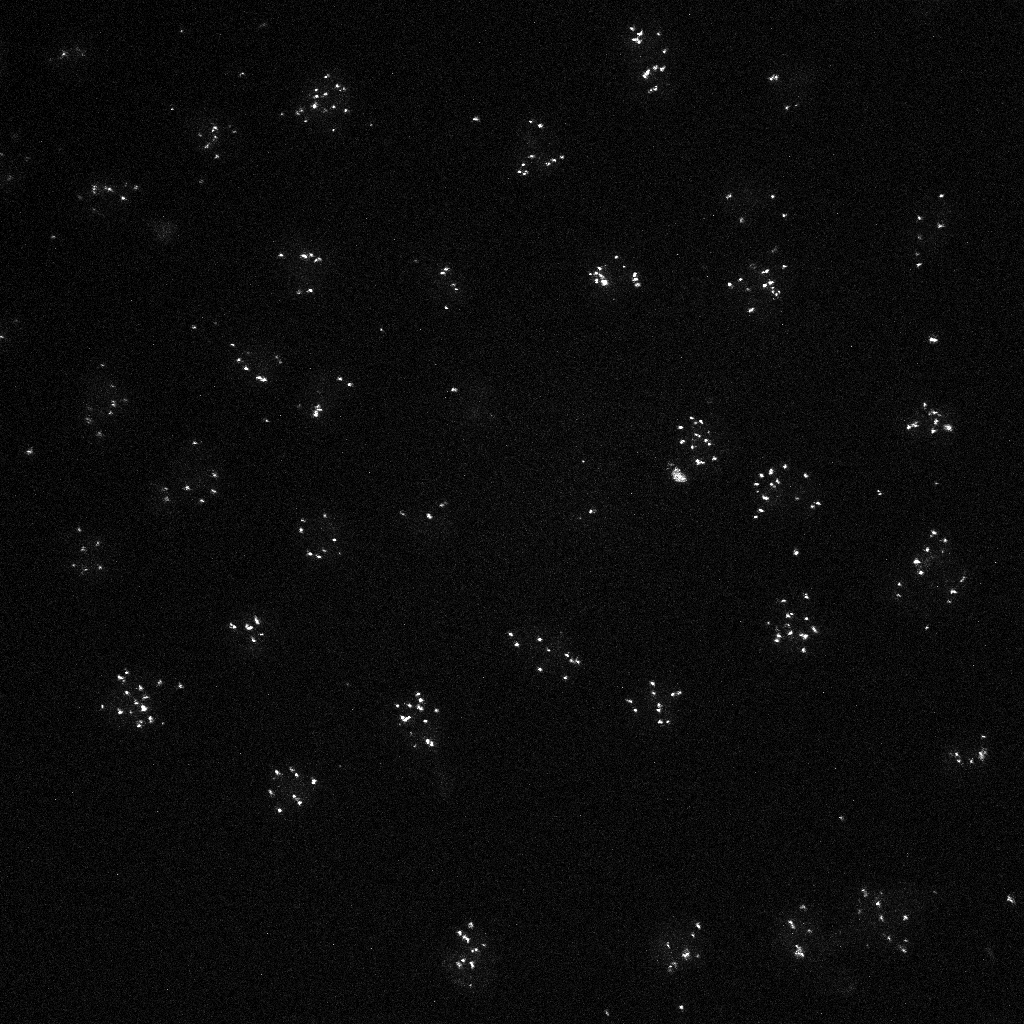

Supplement: Supplementary file 2 — Source data Fig. 1 [file 44318_2024_111_MOESM2_ESM.zip › Figure 1/Figure 1F/+IR.tif]

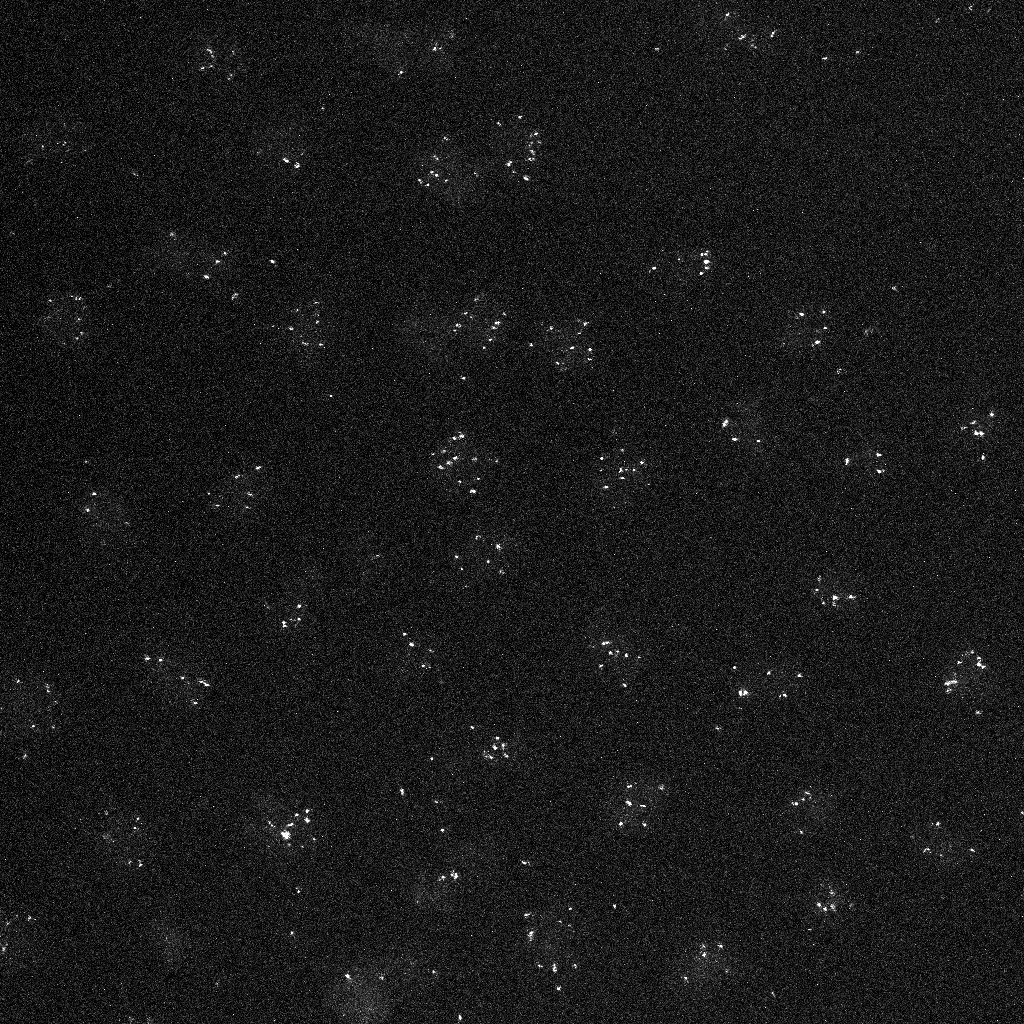

Supplement: Supplementary file 2 — Source data Fig. 1 [file 44318_2024_111_MOESM2_ESM.zip › Figure 1/Figure 1F/-IR 1.tif]

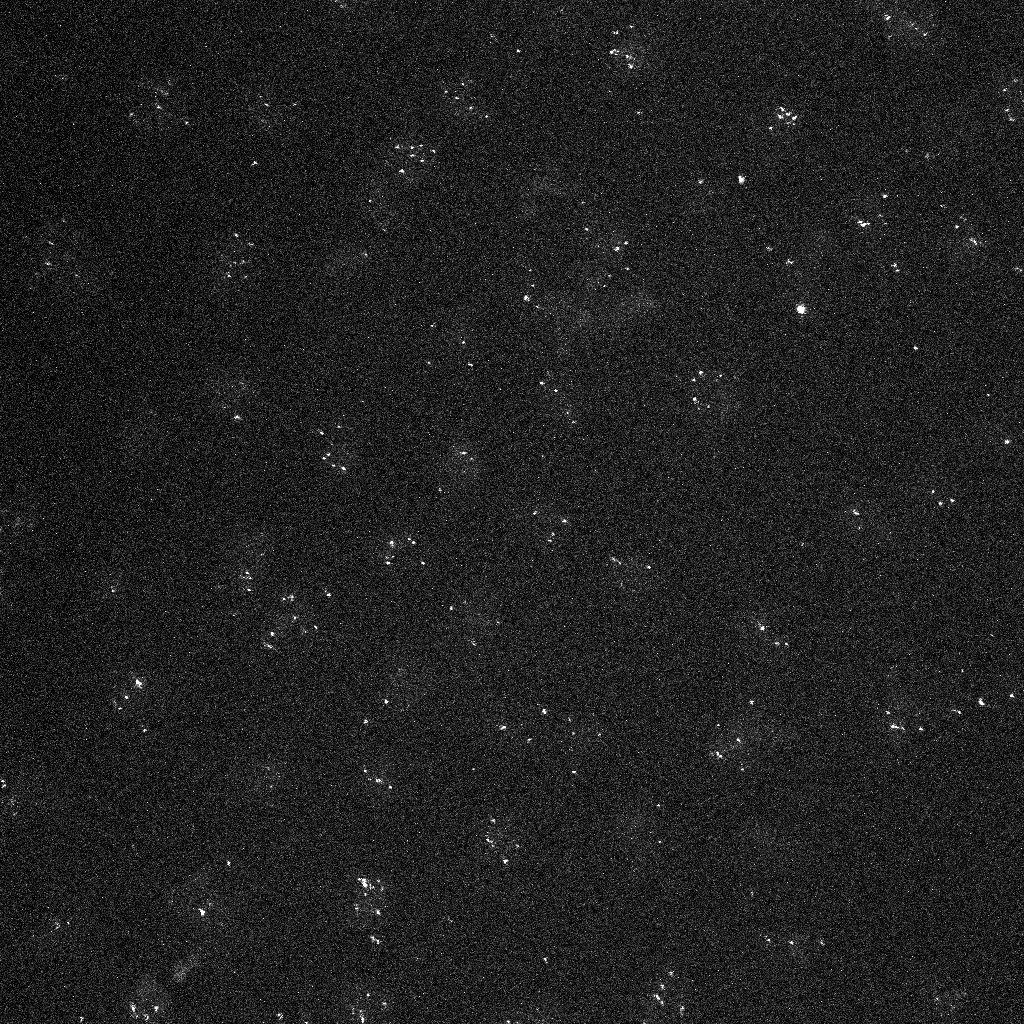

Supplement: Supplementary file 2 — Source data Fig. 1 [file 44318_2024_111_MOESM2_ESM.zip › Figure 1/Figure 1F/-IR 2.tif]

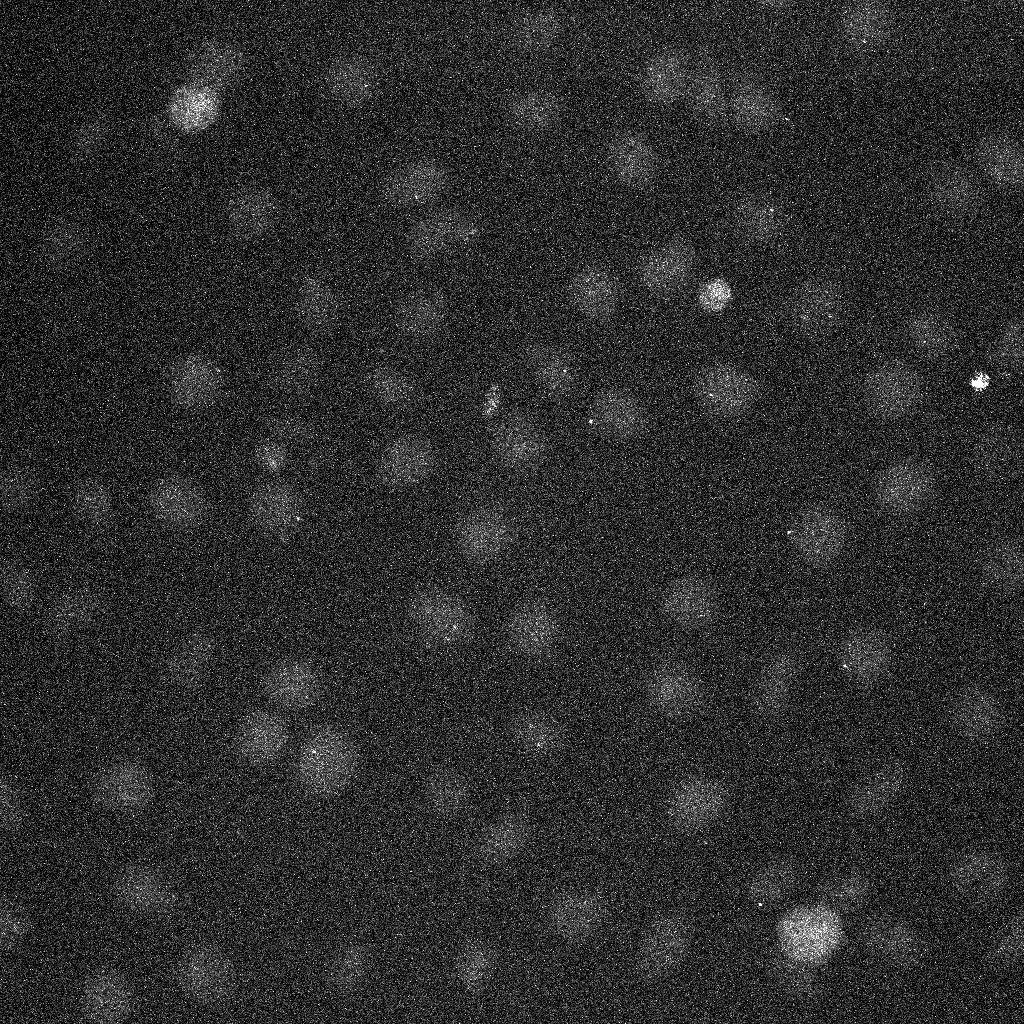

Supplement: Supplementary file 2 — Source data Fig. 1 [file 44318_2024_111_MOESM2_ESM.zip › Figure 1/Figure 1G/-IR.tif]

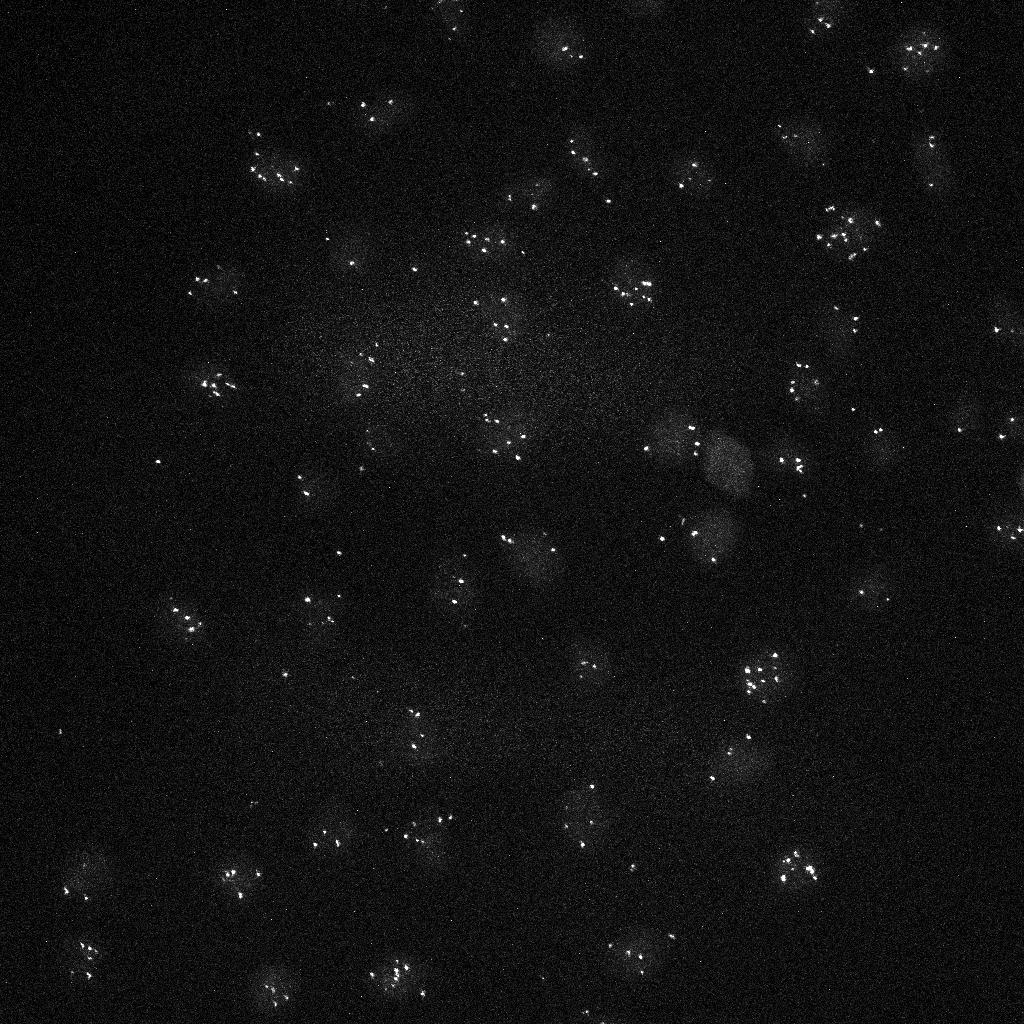

Supplement: Supplementary file 2 — Source data Fig. 1 [file 44318_2024_111_MOESM2_ESM.zip › Figure 1/Figure 1G/+IR.tif]

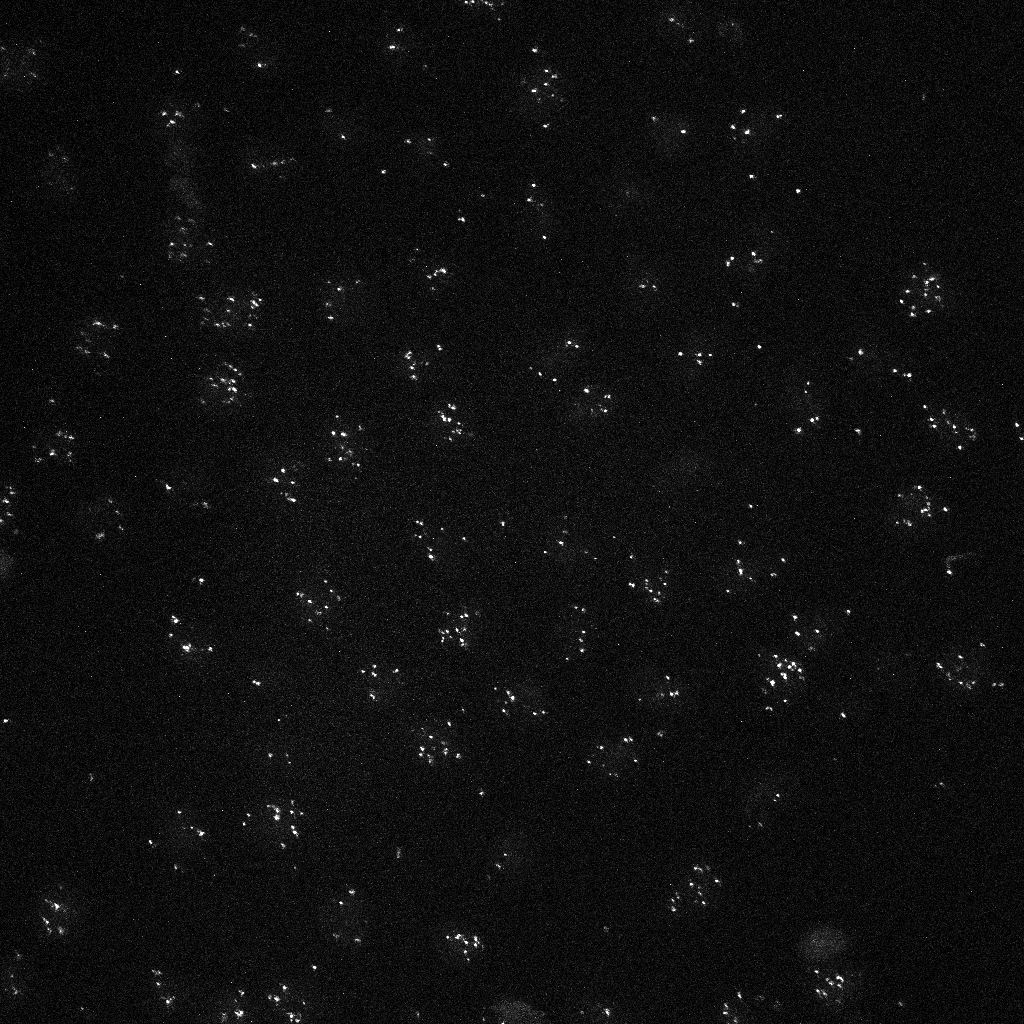

Supplement: Supplementary file 2 — Source data Fig. 1 [file 44318_2024_111_MOESM2_ESM.zip › Figure 1/Figure 1G/+IR +ATMi.tif]

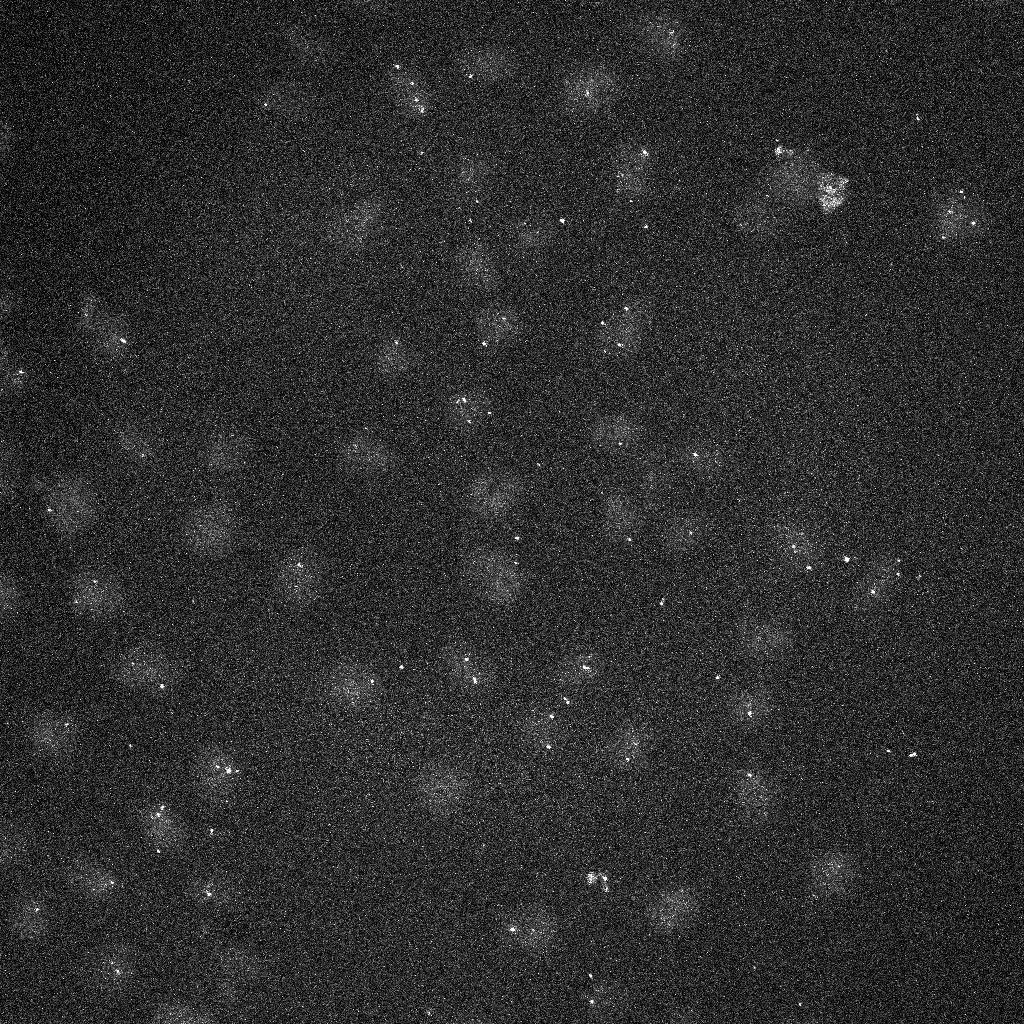

Supplement: Supplementary file 2 — Source data Fig. 1 [file 44318_2024_111_MOESM2_ESM.zip › Figure 1/Figure 1G/+IR +PARPi.tif]

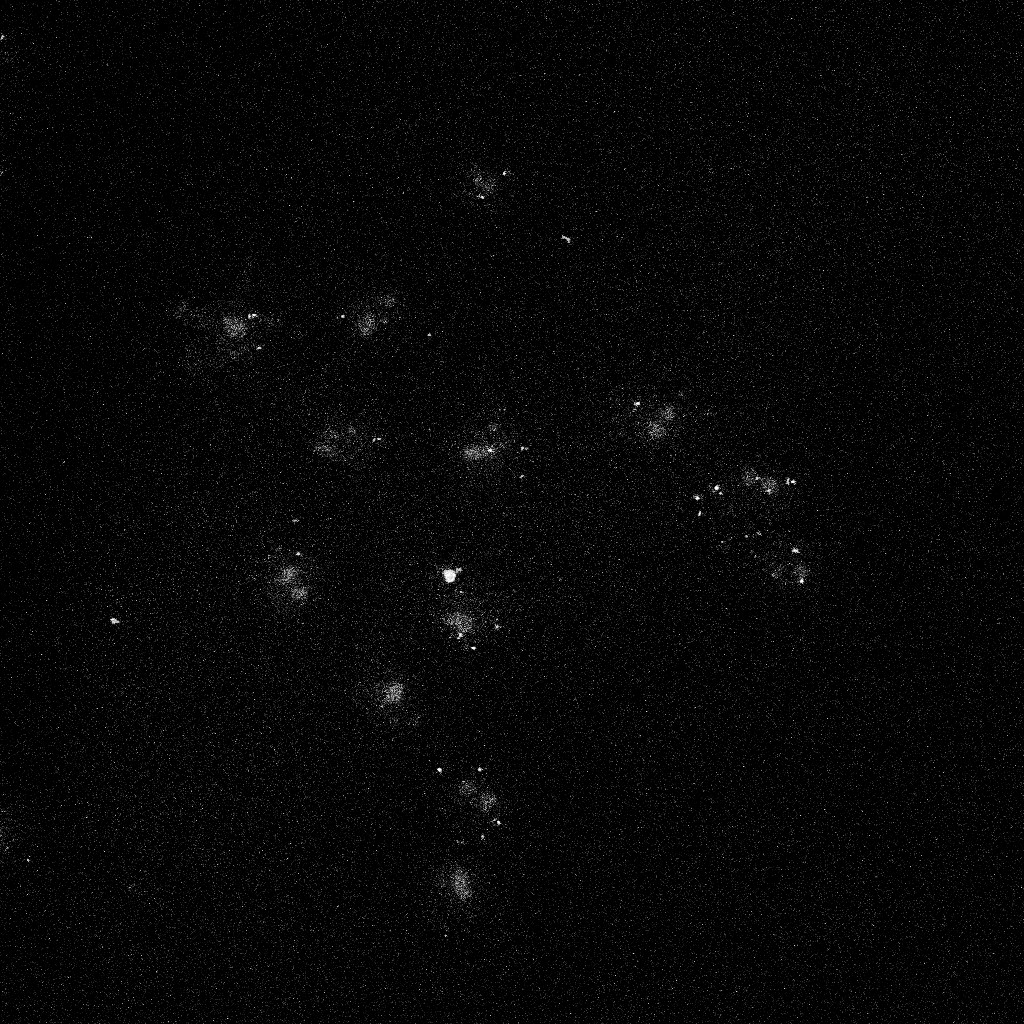

Supplement: Supplementary file 2 — Source data Fig. 1 [file 44318_2024_111_MOESM2_ESM.zip › Figure 1/Figure 1E/+IR +RNH1.tif]

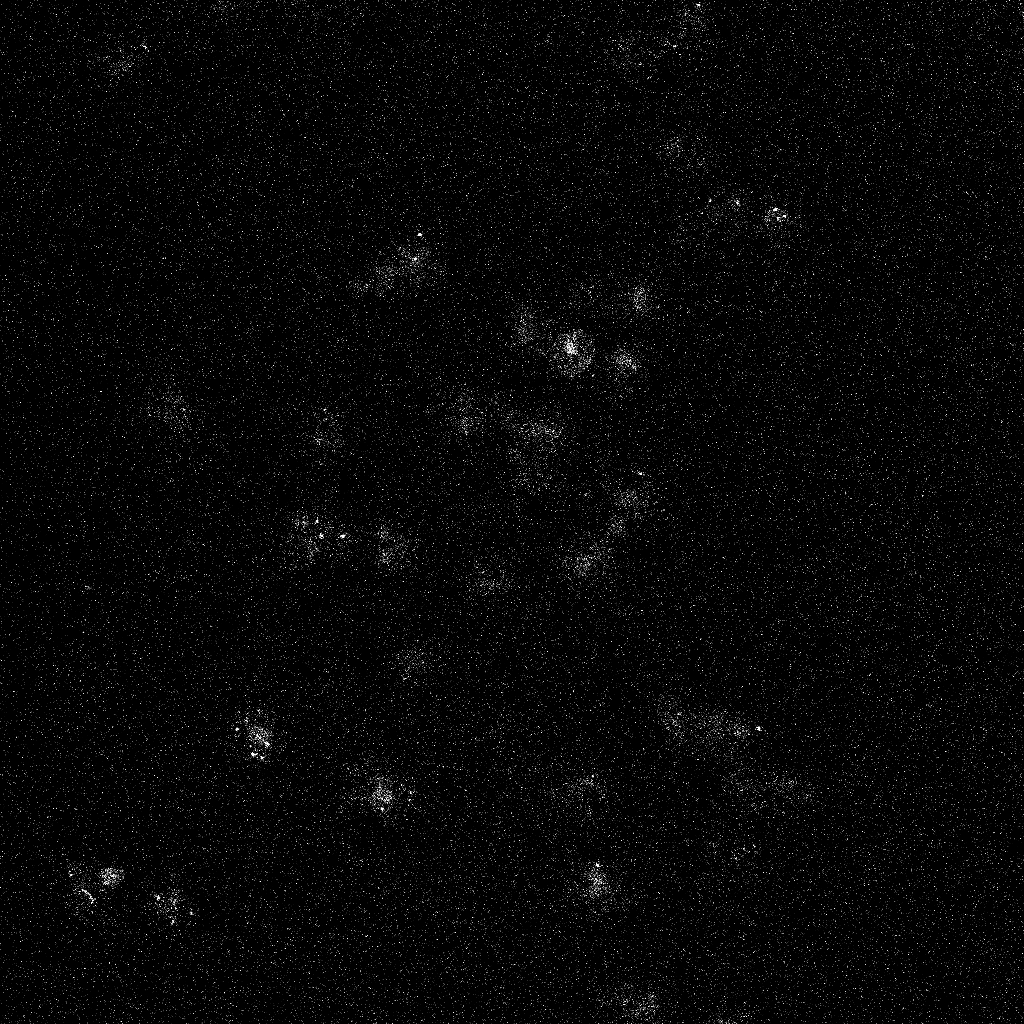

Supplement: Supplementary file 2 — Source data Fig. 1 [file 44318_2024_111_MOESM2_ESM.zip › Figure 1/Figure 1E/-IR.tif]

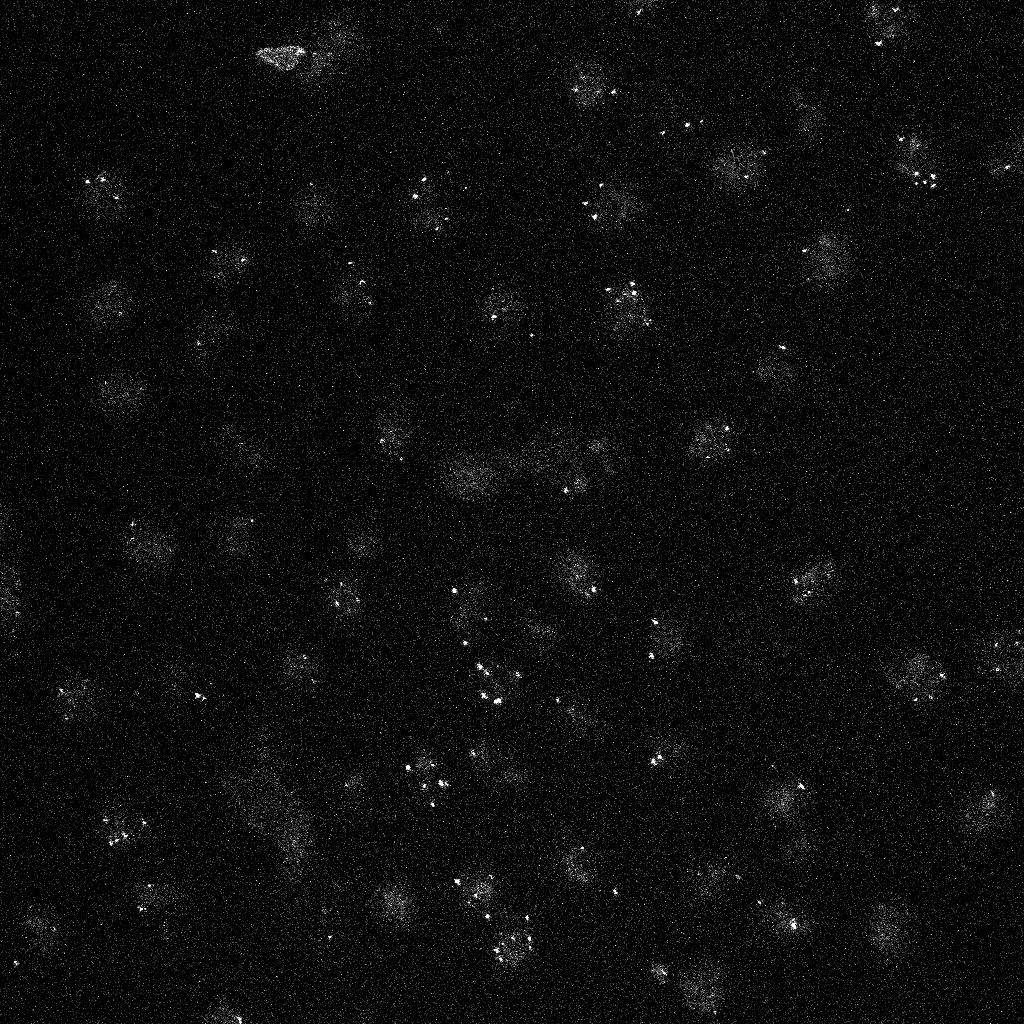

Supplement: Supplementary file 2 — Source data Fig. 1 [file 44318_2024_111_MOESM2_ESM.zip › Figure 1/Figure 1E/+IR.tif]

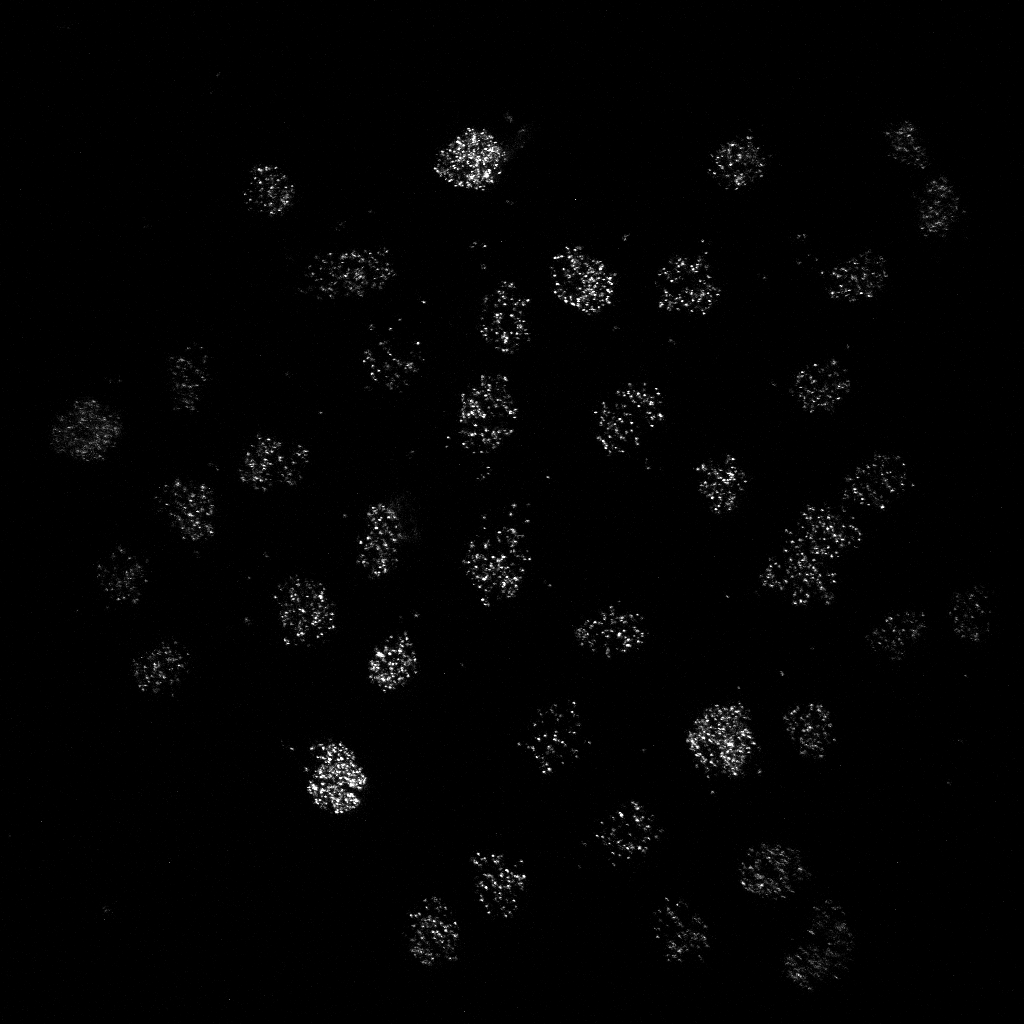

Supplement: Supplementary file 3 — Source data Fig. 2 [file 44318_2024_111_MOESM3_ESM.zip › Figure 2/Figure 2C/+IR +RNH1.tif]

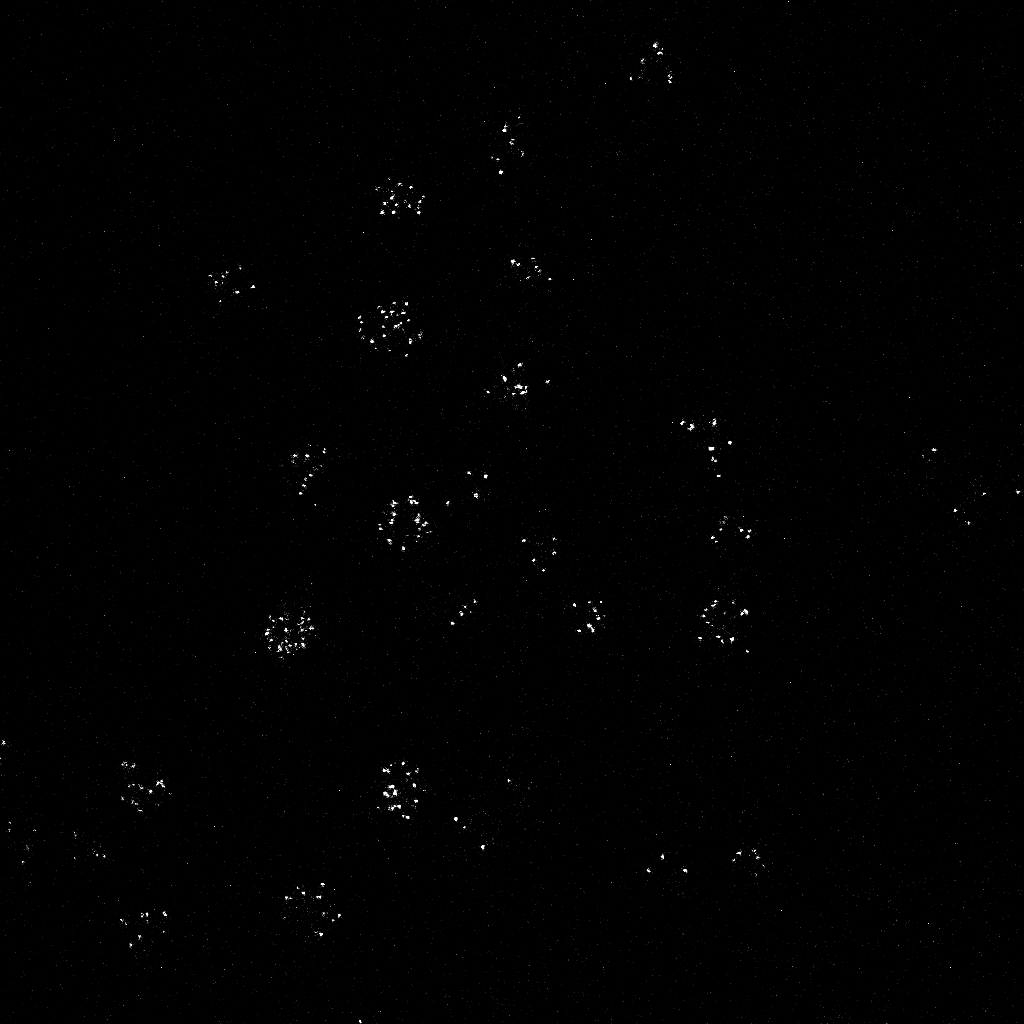

Supplement: Supplementary file 3 — Source data Fig. 2 [file 44318_2024_111_MOESM3_ESM.zip › Figure 2/Figure 2C/-IR.tif]

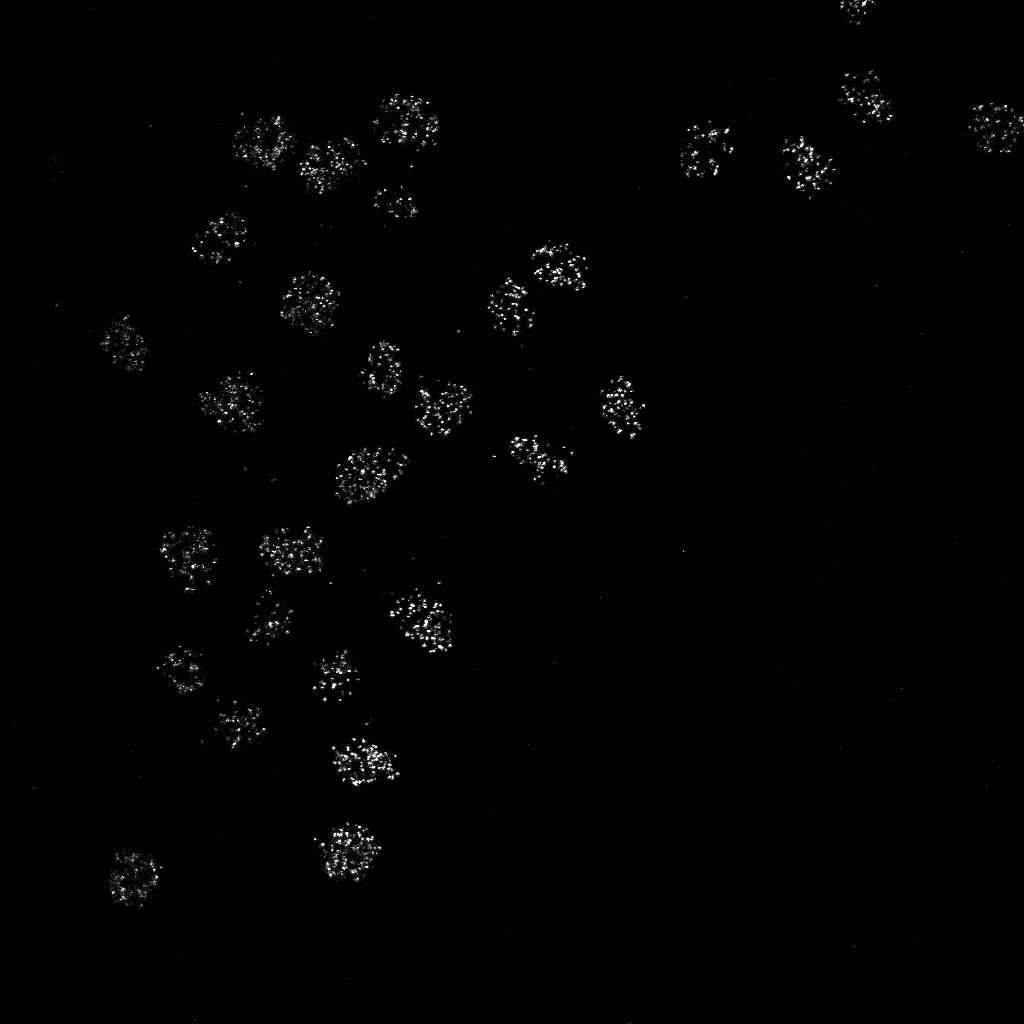

Supplement: Supplementary file 3 — Source data Fig. 2 [file 44318_2024_111_MOESM3_ESM.zip › Figure 2/Figure 2C/+IR.tif]

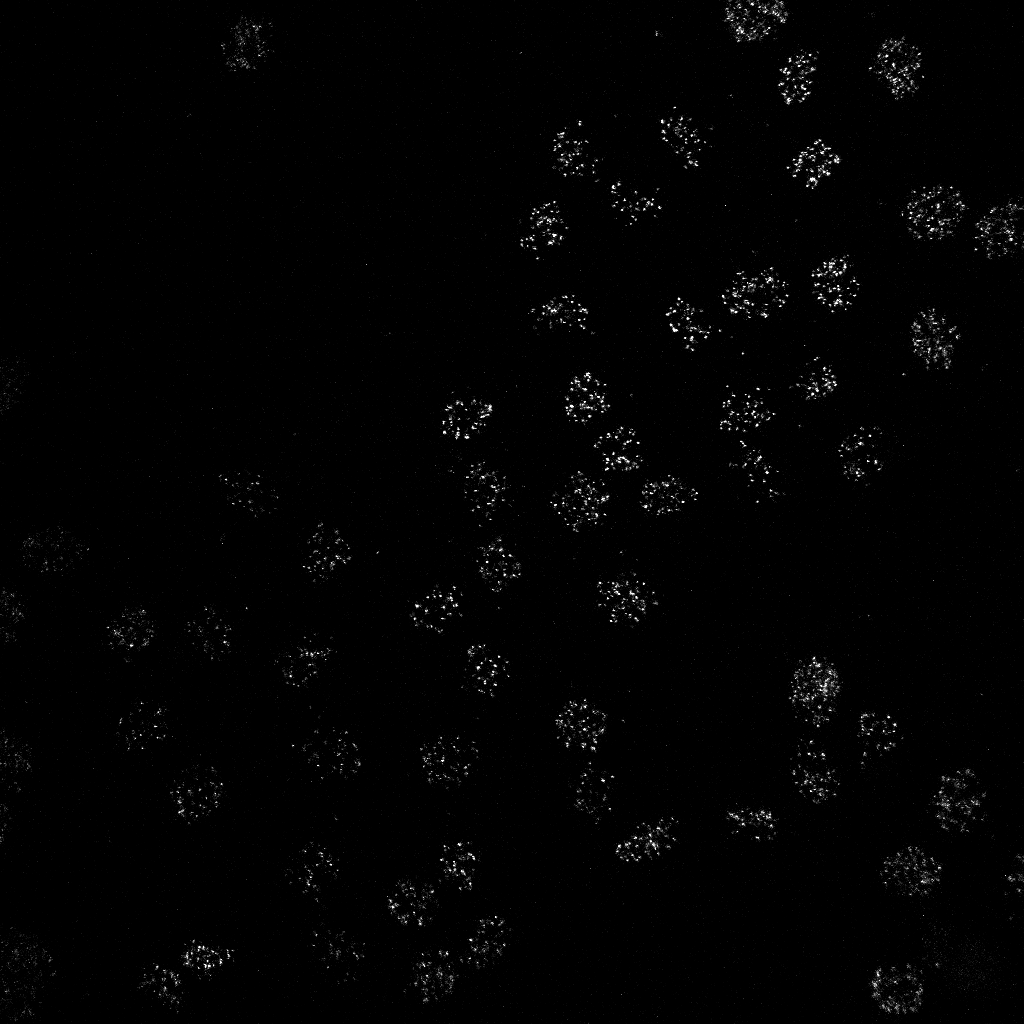

Supplement: Supplementary file 3 — Source data Fig. 2 [file 44318_2024_111_MOESM3_ESM.zip › Figure 2/Figure 2C/+IR +TPL3.tif]

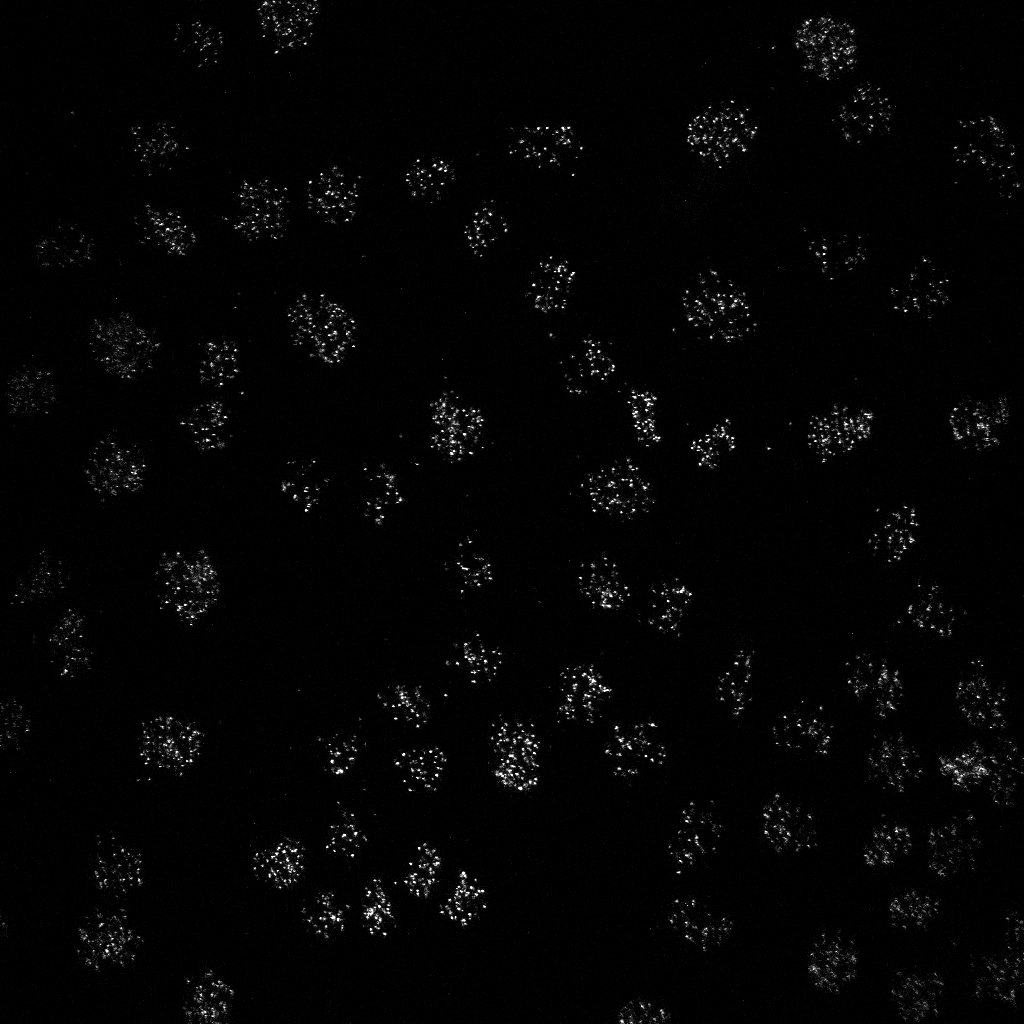

Supplement: Supplementary file 3 — Source data Fig. 2 [file 44318_2024_111_MOESM3_ESM.zip › Figure 2/Figure 2C/+ IR +DRB.tif]

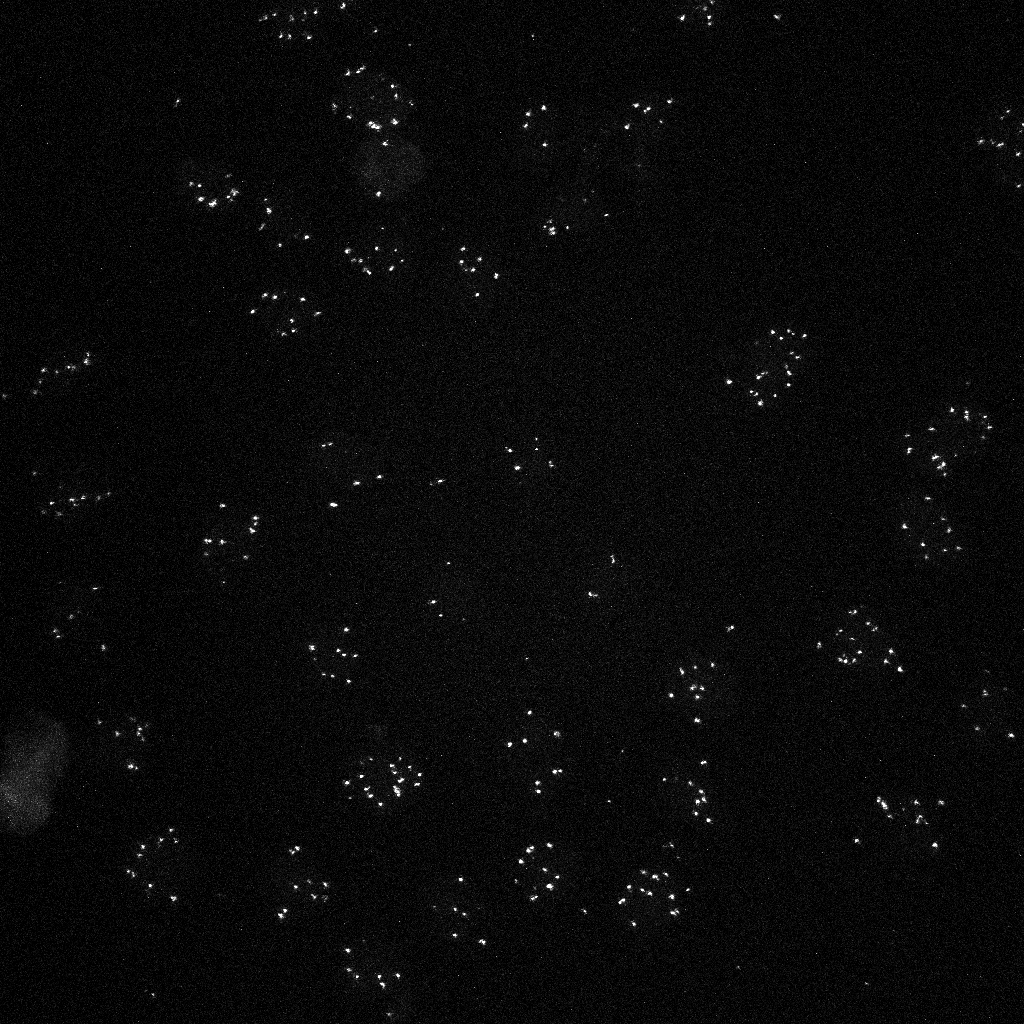

Supplement: Supplementary file 3 — Source data Fig. 2 [file 44318_2024_111_MOESM3_ESM.zip › Figure 2/Figure 2D/+IR +MBD3.tif]

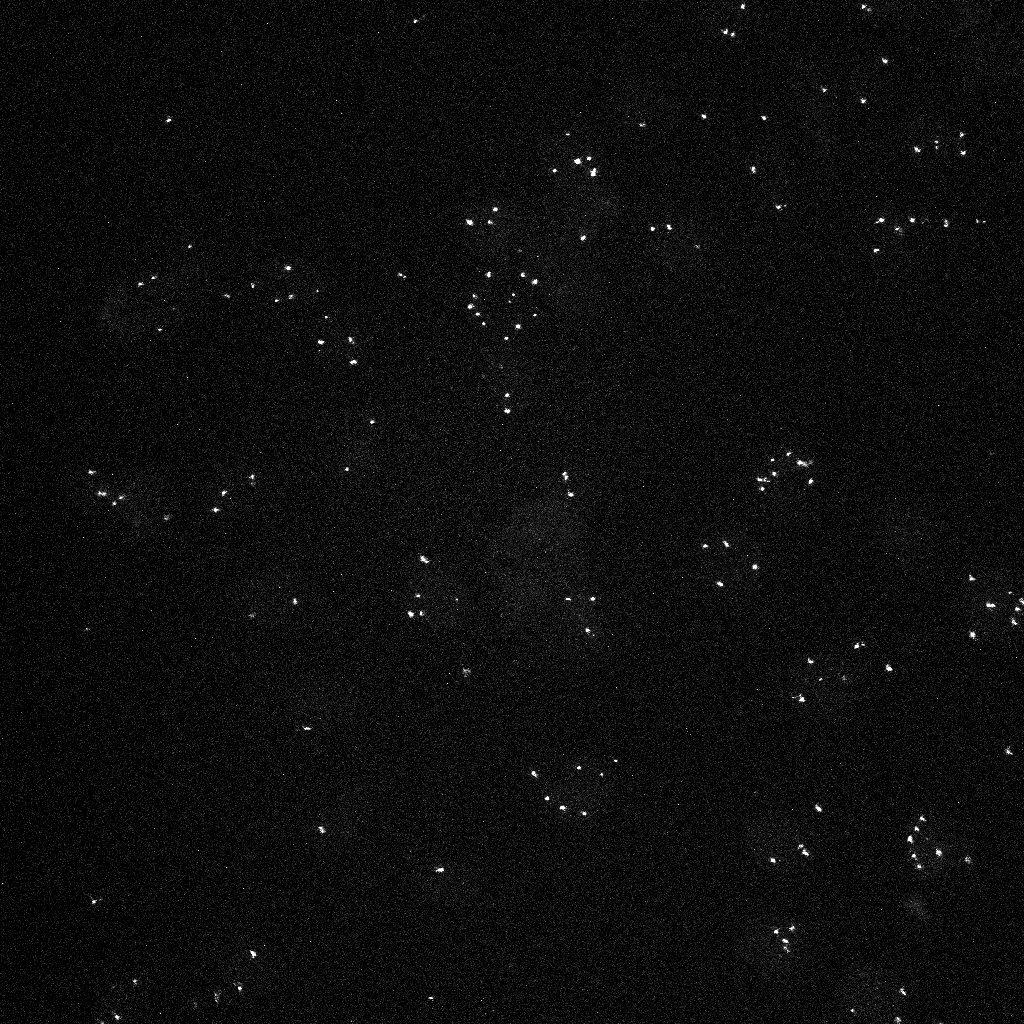

Supplement: Supplementary file 3 — Source data Fig. 2 [file 44318_2024_111_MOESM3_ESM.zip › Figure 2/Figure 2D/+IR +RNH1.tif]

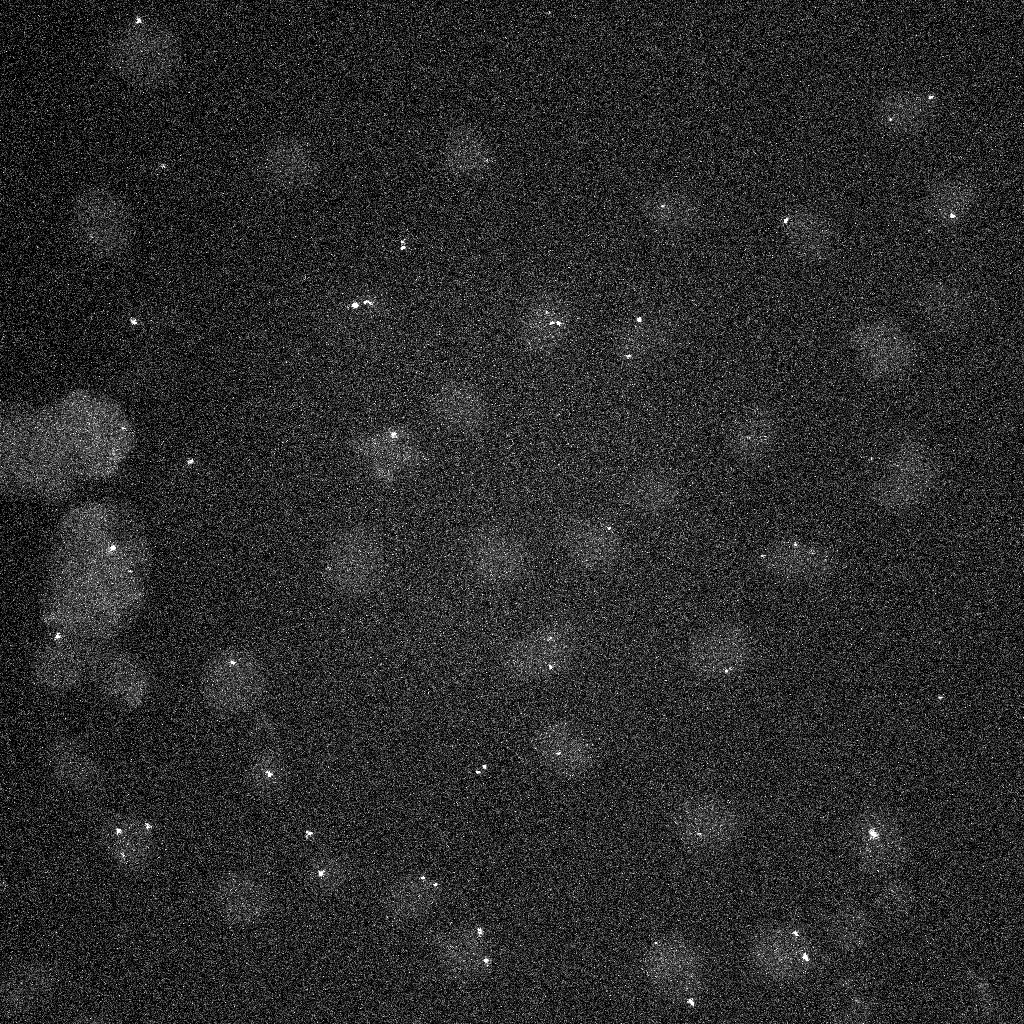

Supplement: Supplementary file 3 — Source data Fig. 2 [file 44318_2024_111_MOESM3_ESM.zip › Figure 2/Figure 2D/-IR.tif]

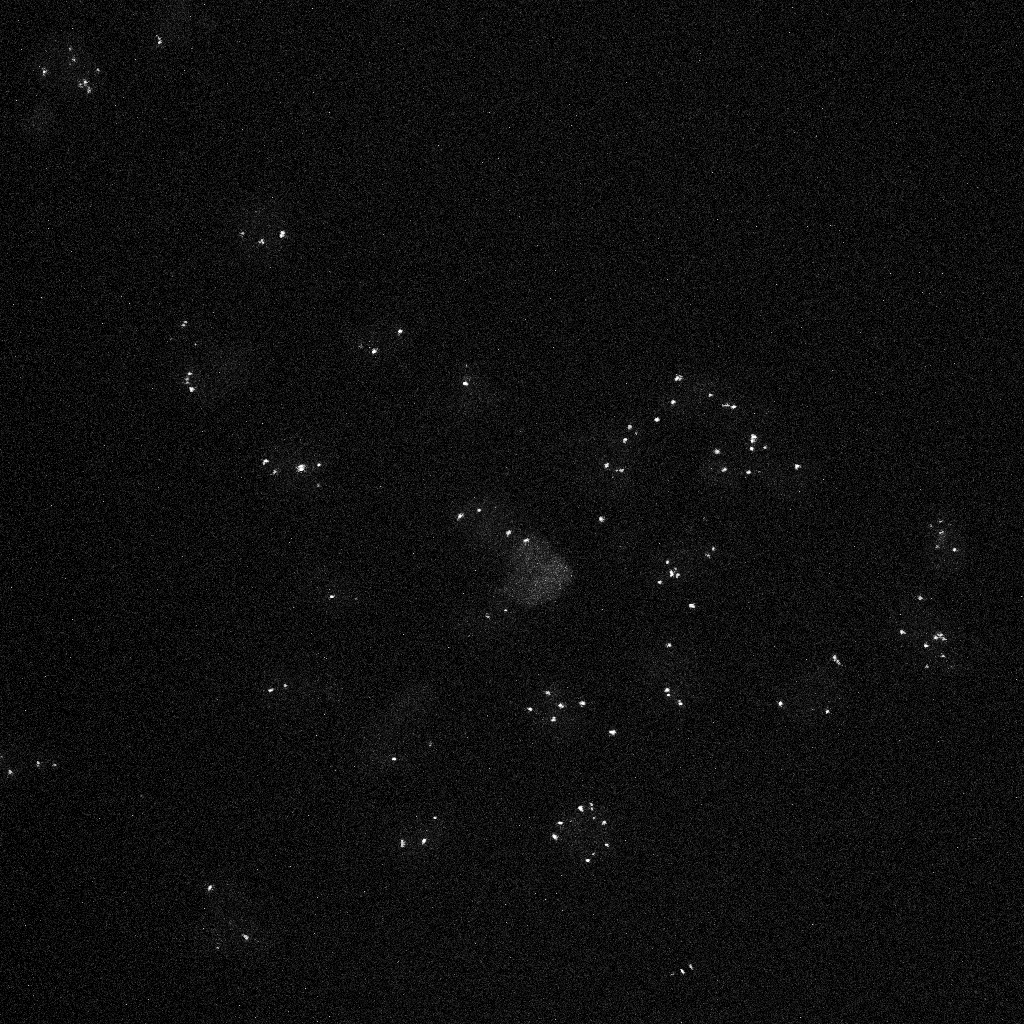

Supplement: Supplementary file 3 — Source data Fig. 2 [file 44318_2024_111_MOESM3_ESM.zip › Figure 2/Figure 2D/+IR +siGATAD2B.tif]

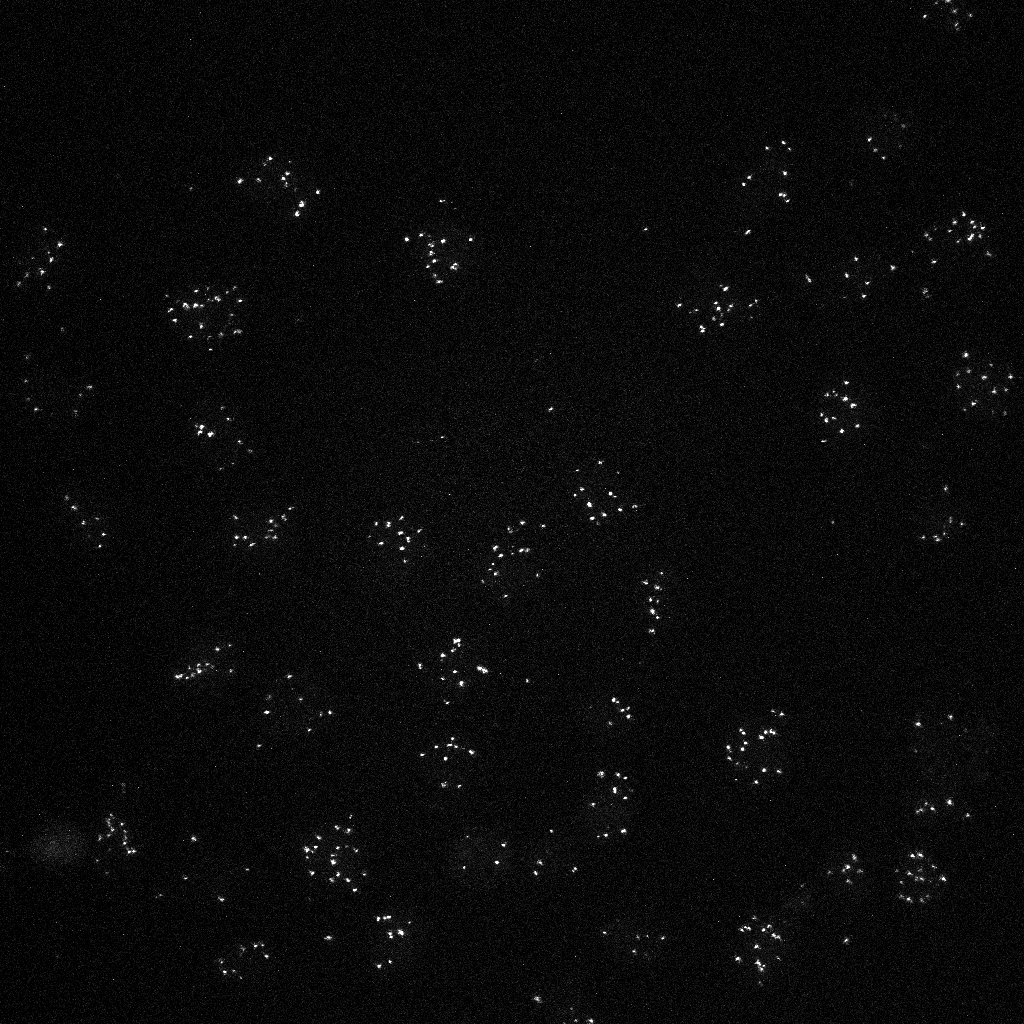

Supplement: Supplementary file 3 — Source data Fig. 2 [file 44318_2024_111_MOESM3_ESM.zip › Figure 2/Figure 2D/+IR.tif]

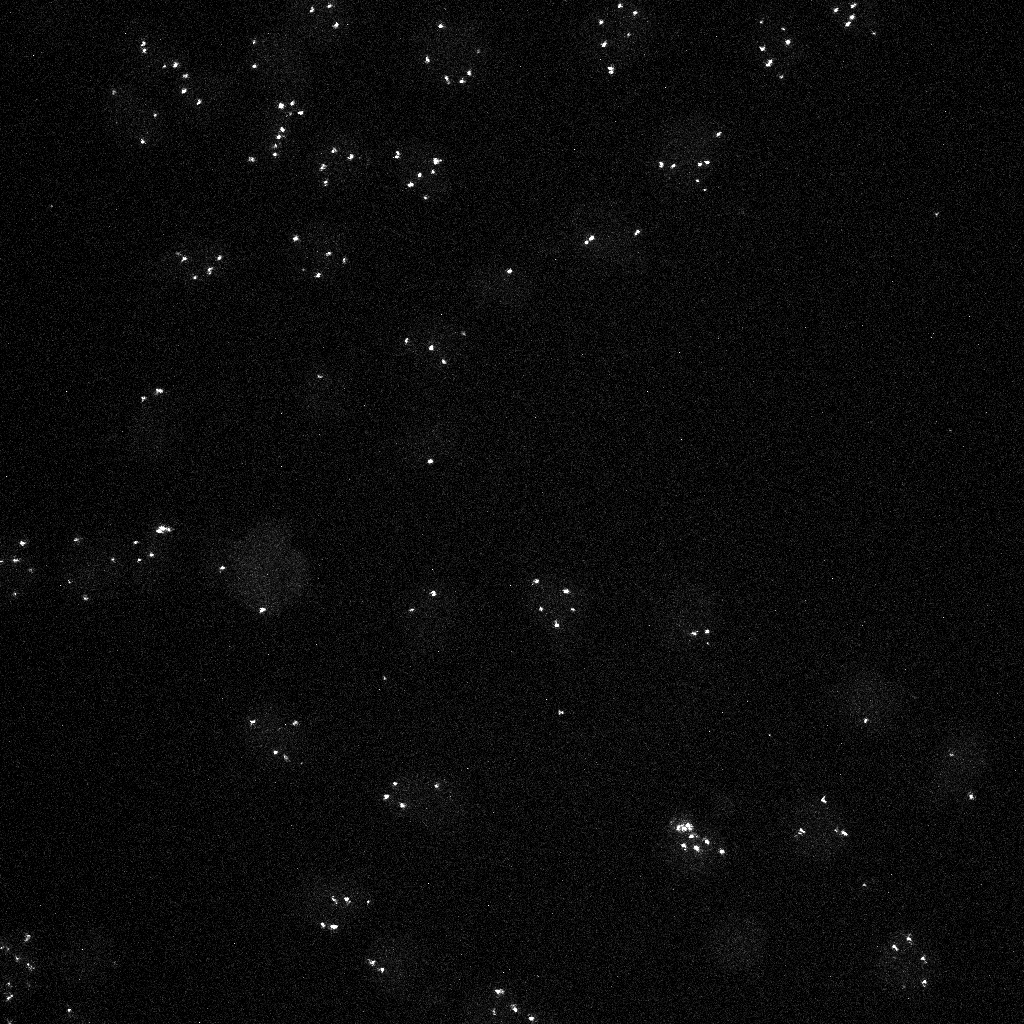

Supplement: Supplementary file 3 — Source data Fig. 2 [file 44318_2024_111_MOESM3_ESM.zip › Figure 2/Figure 2D/+IR +TPL3.tif]

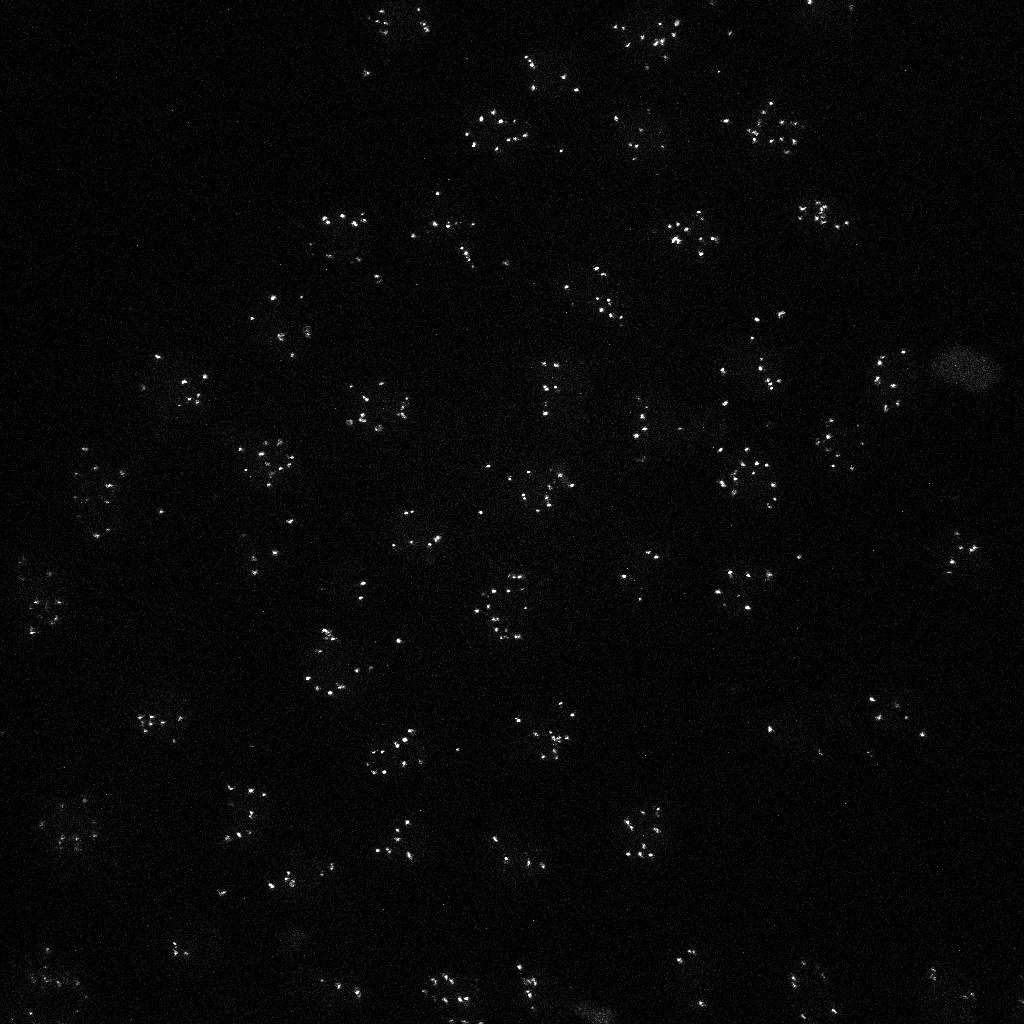

Supplement: Supplementary file 3 — Source data Fig. 2 [file 44318_2024_111_MOESM3_ESM.zip › Figure 2/Figure 2D/+IR +DRB.tif]

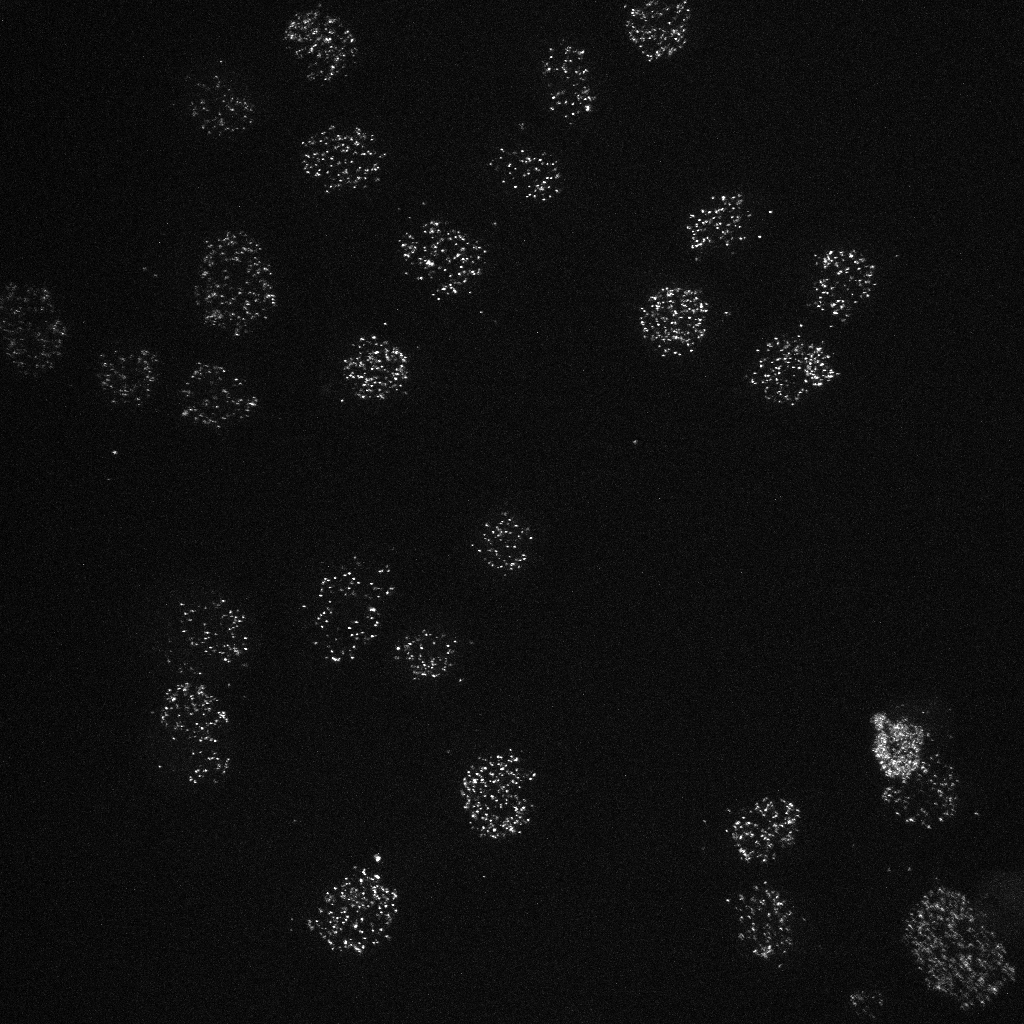

Supplement: Supplementary file 4 — Source data Fig. 4 [file 44318_2024_111_MOESM4_ESM.zip › Figure 4/Figure 4F/+IR siMBD3.tif]

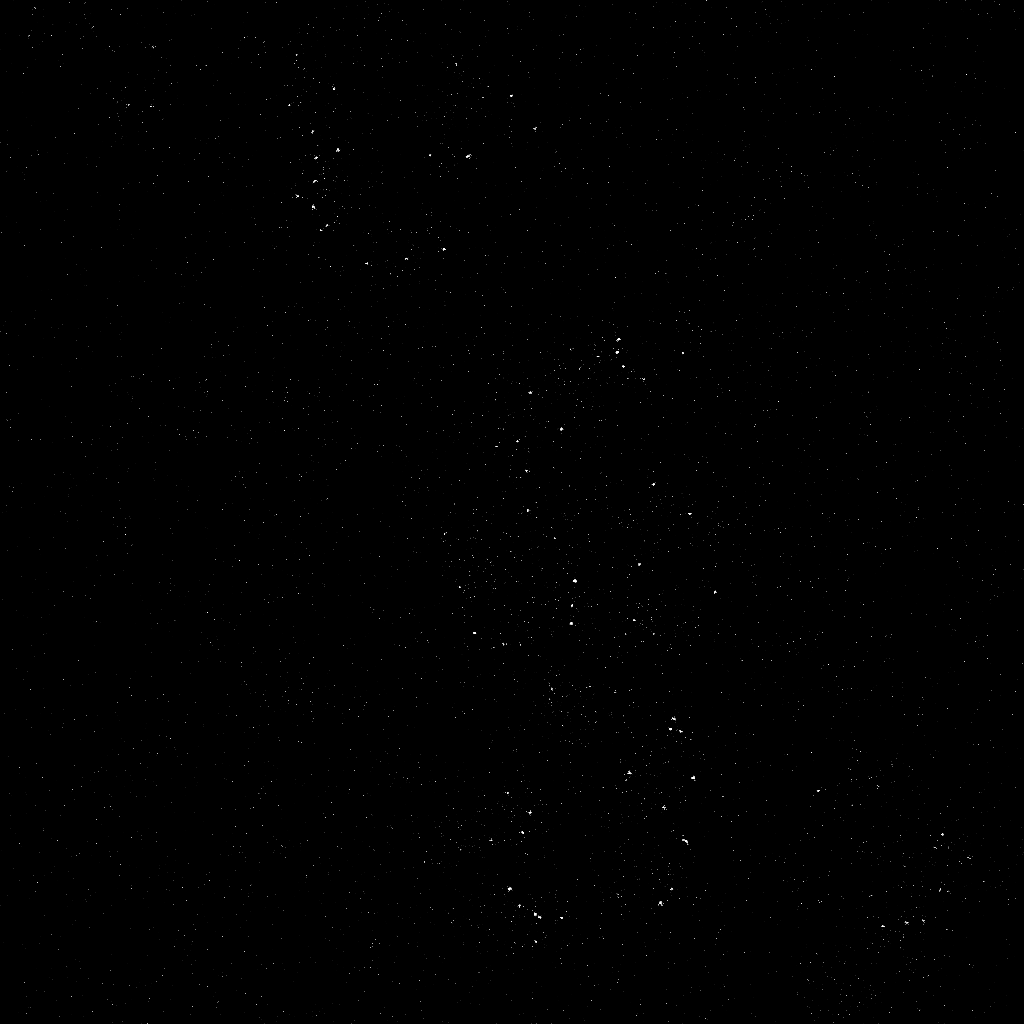

Supplement: Supplementary file 4 — Source data Fig. 4 [file 44318_2024_111_MOESM4_ESM.zip › Figure 4/Figure 4F/pan-H4 ace.tif]

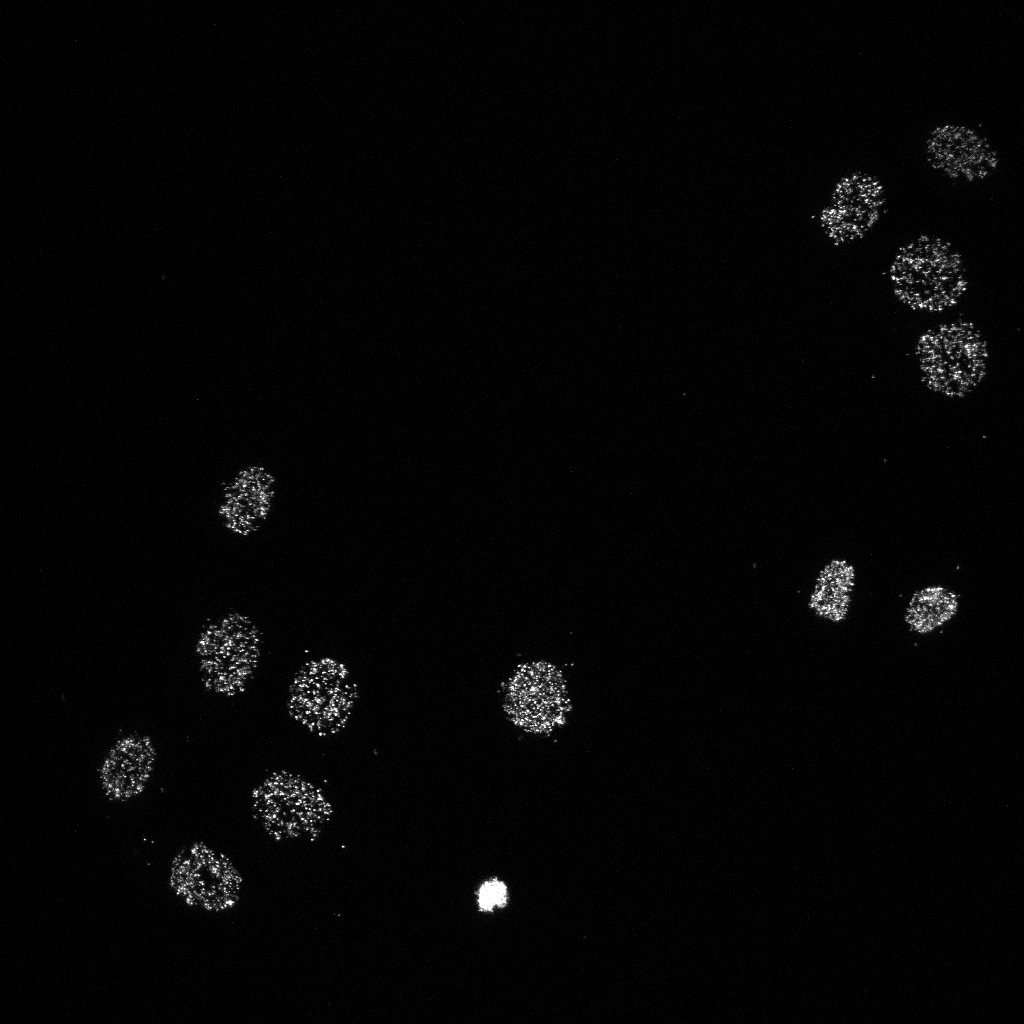

Supplement: Supplementary file 4 — Source data Fig. 4 [file 44318_2024_111_MOESM4_ESM.zip › Figure 4/Figure 4F/+IR +RNH1.tif]

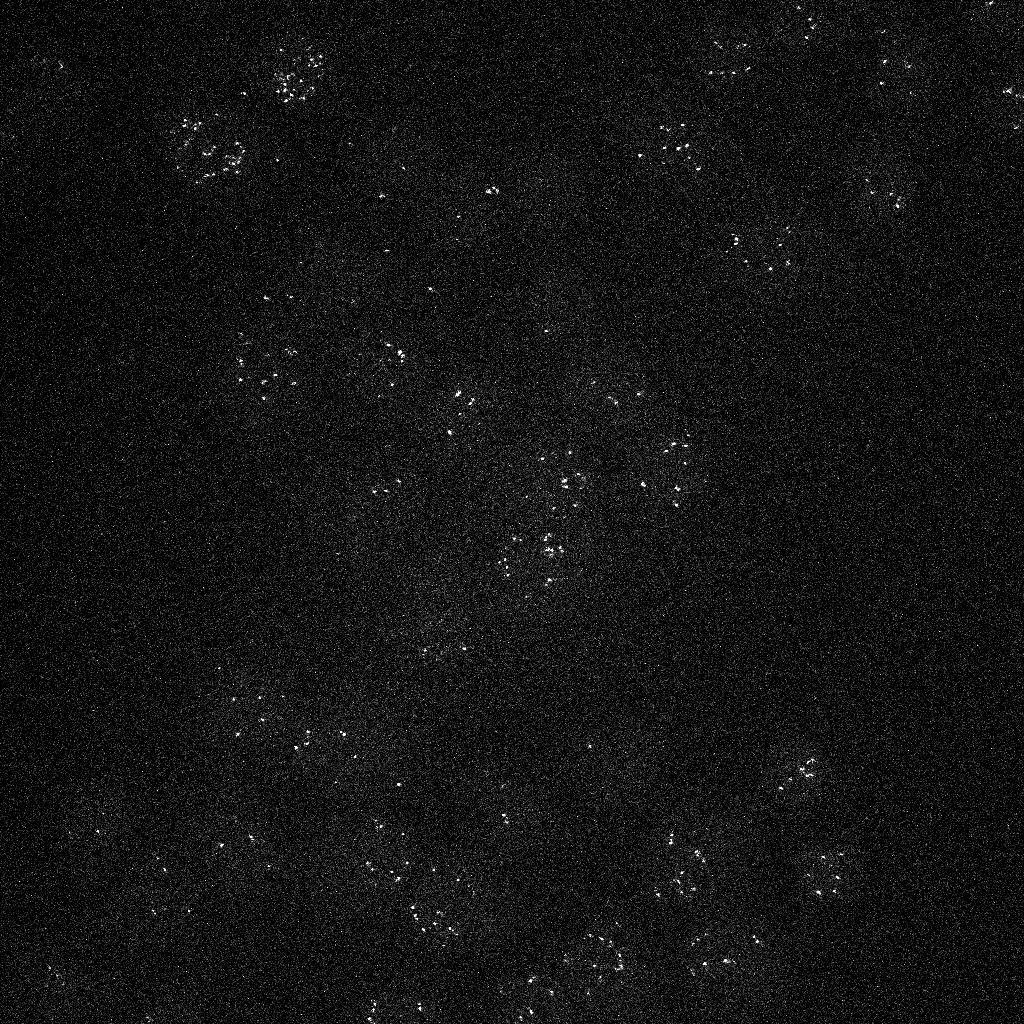

Supplement: Supplementary file 4 — Source data Fig. 4 [file 44318_2024_111_MOESM4_ESM.zip › Figure 4/Figure 4F/-IR.tif]

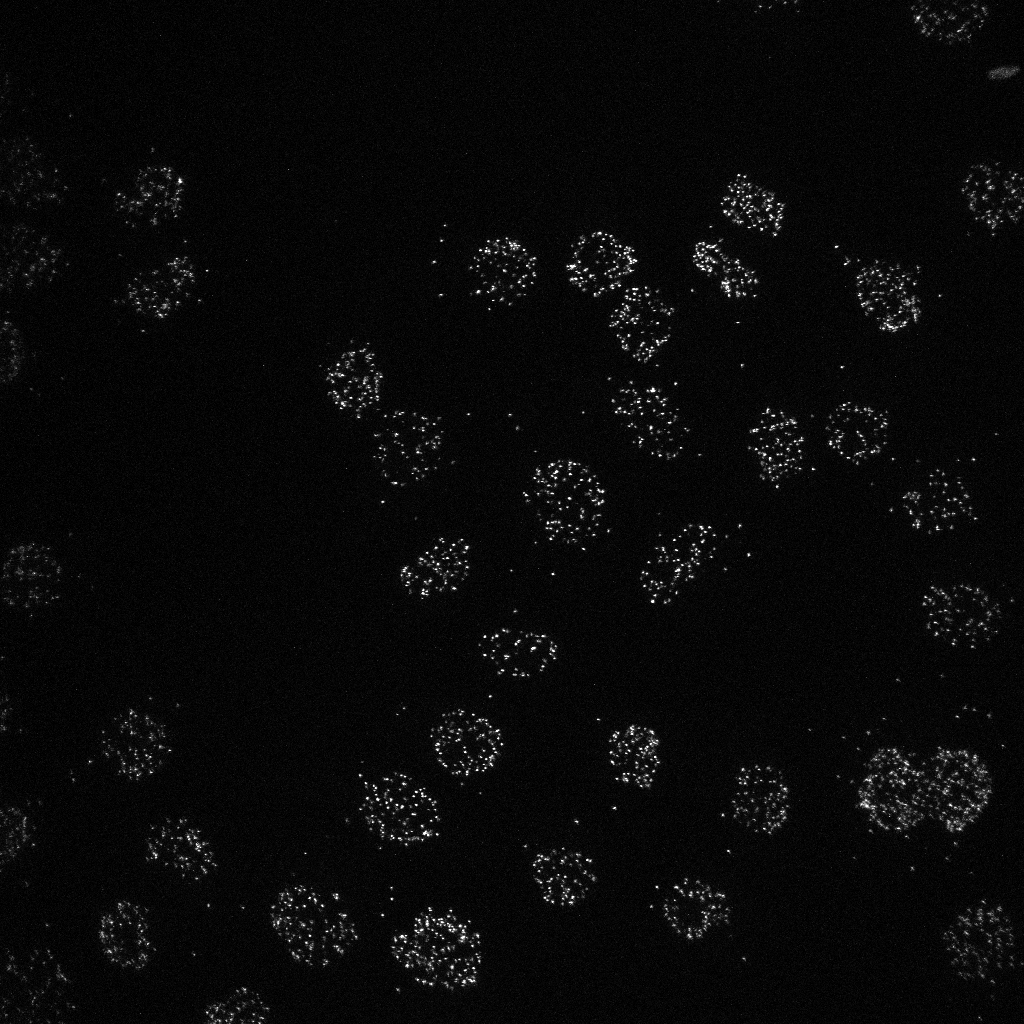

Supplement: Supplementary file 4 — Source data Fig. 4 [file 44318_2024_111_MOESM4_ESM.zip › Figure 4/Figure 4F/+IR.tif]

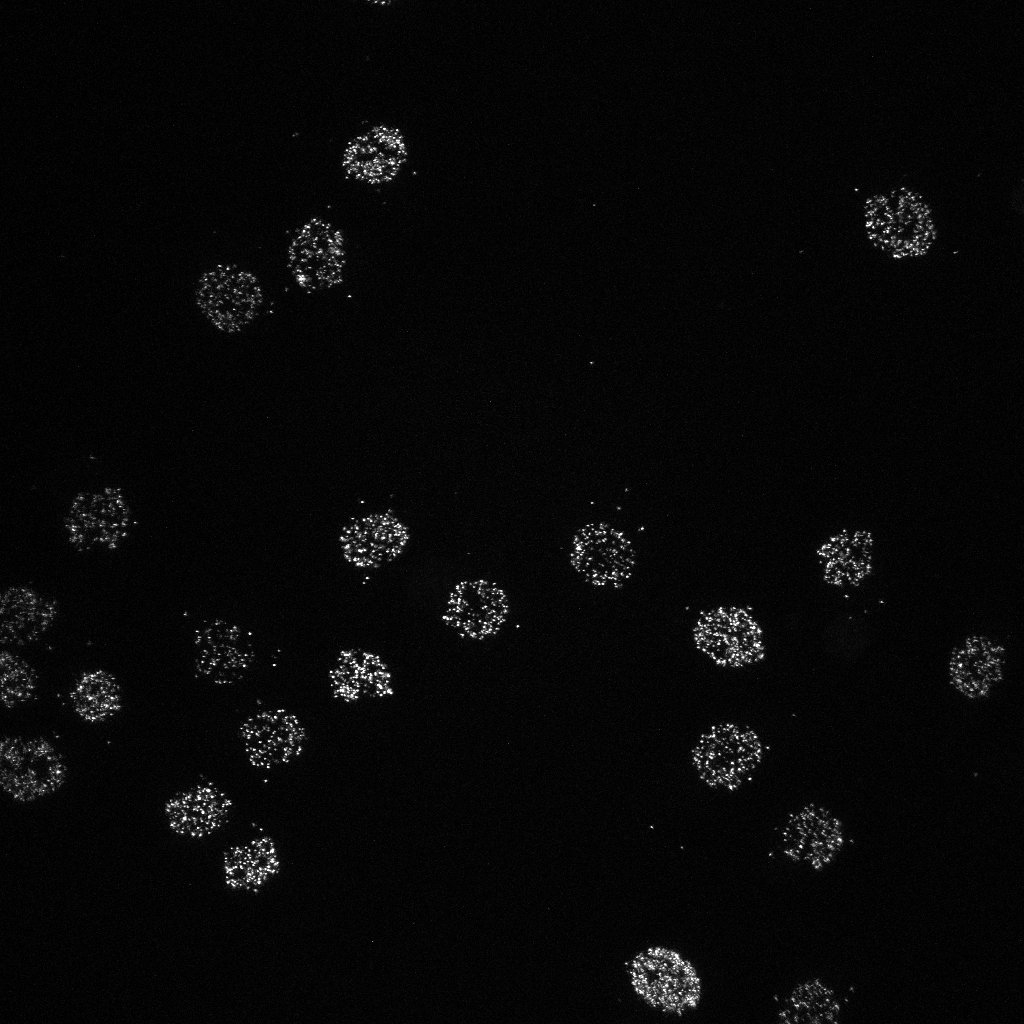

Supplement: Supplementary file 4 — Source data Fig. 4 [file 44318_2024_111_MOESM4_ESM.zip › Figure 4/Figure 4F/+IR siGATAD2B.tif]

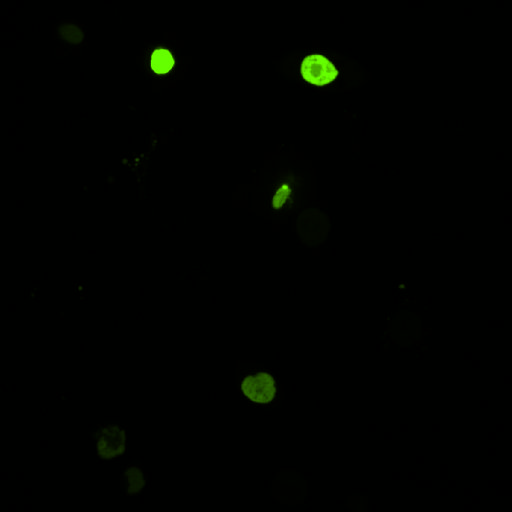

Supplement: Supplementary file 5 — Source data Fig. 5 [file 44318_2024_111_MOESM5_ESM.zip › Figure 5/Figure 5D/siGATAD2B.vsi]

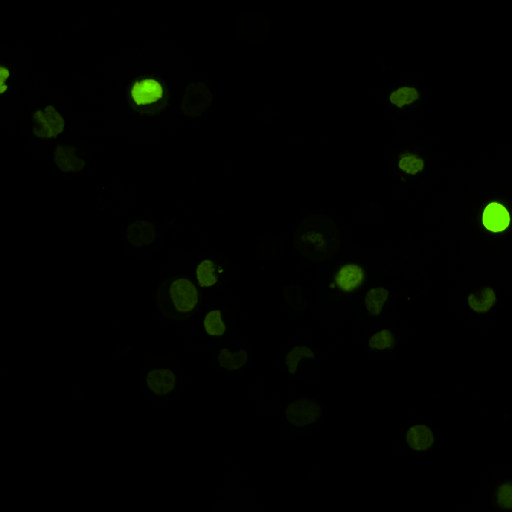

Supplement: Supplementary file 5 — Source data Fig. 5 [file 44318_2024_111_MOESM5_ESM.zip › Figure 5/Figure 5D/nc.vsi]

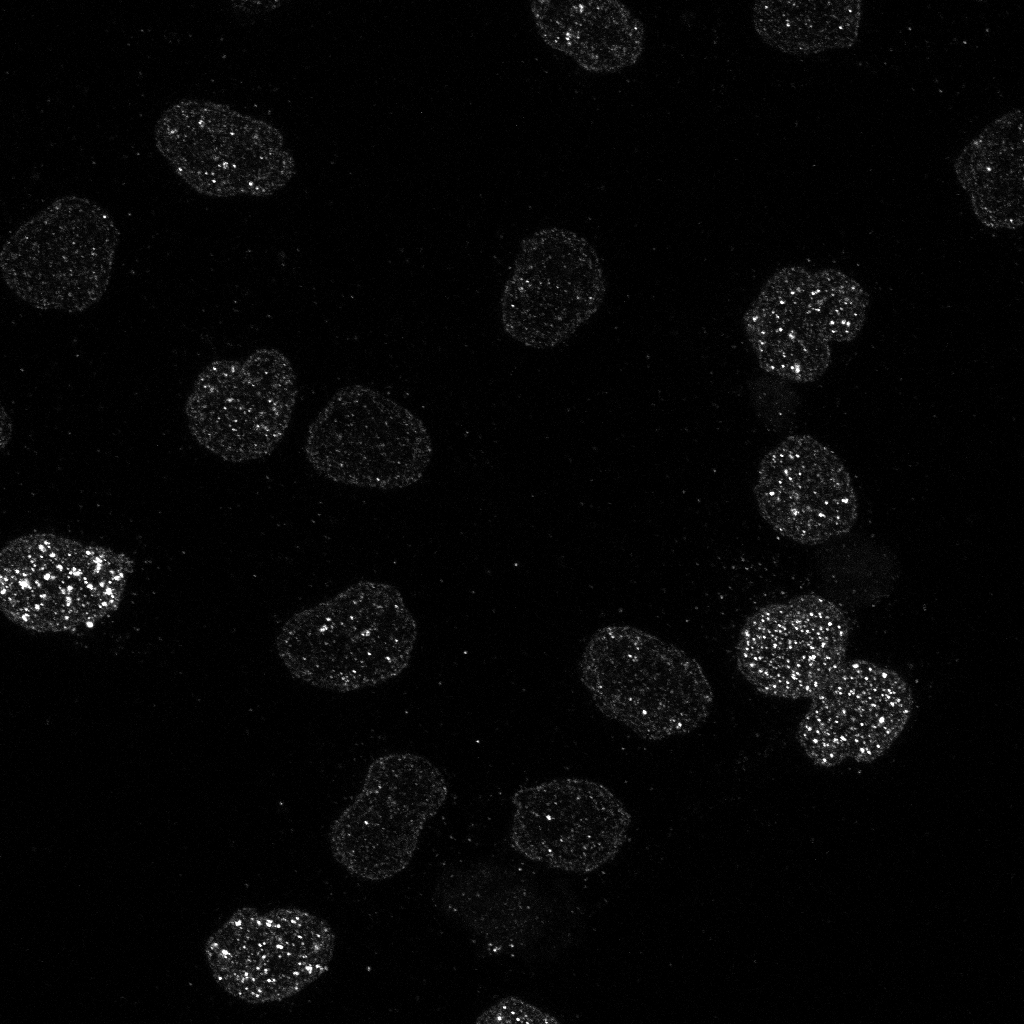

Supplement: Supplementary file 6 — Source data Fig. 6 [file 44318_2024_111_MOESM6_ESM.zip › Figure 6/Figure 6A/+IR siBRCA1.tif]

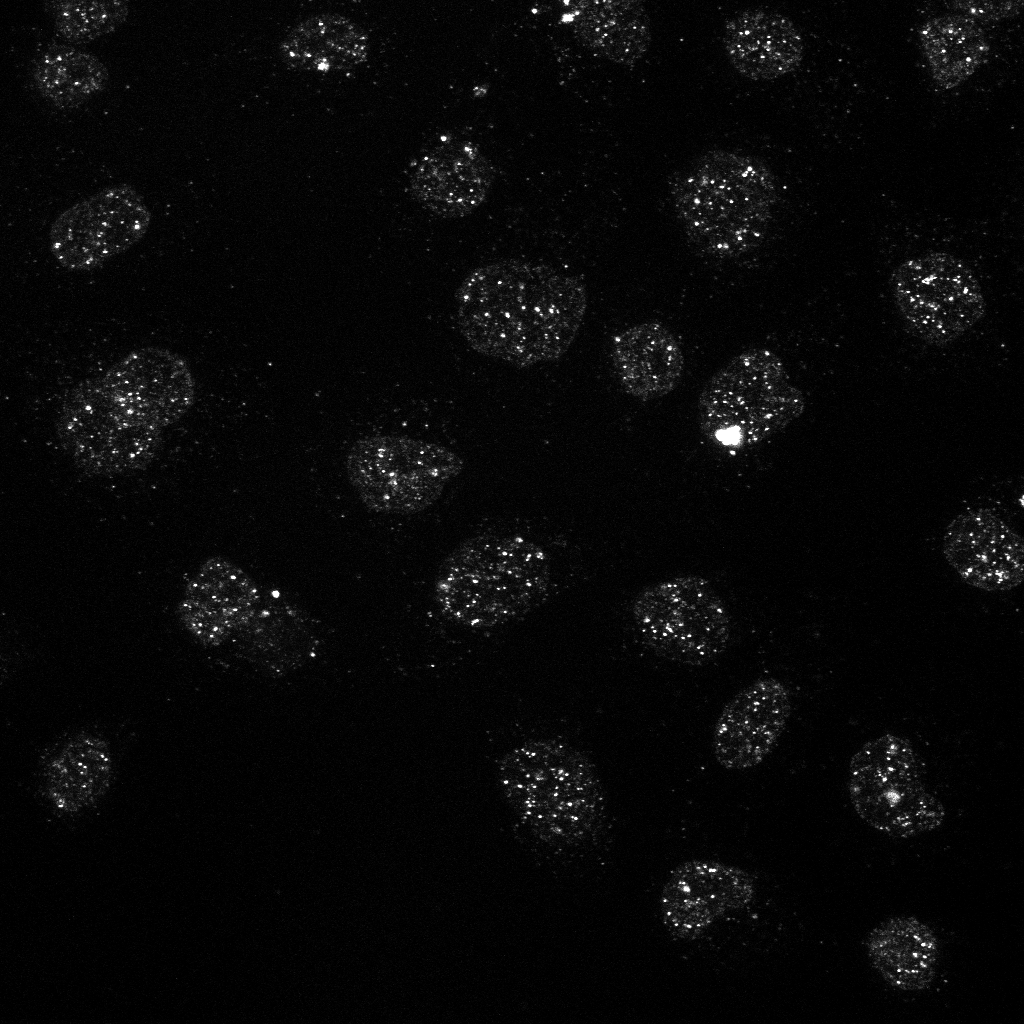

Supplement: Supplementary file 6 — Source data Fig. 6 [file 44318_2024_111_MOESM6_ESM.zip › Figure 6/Figure 6A/+IR +RNH1.tif]

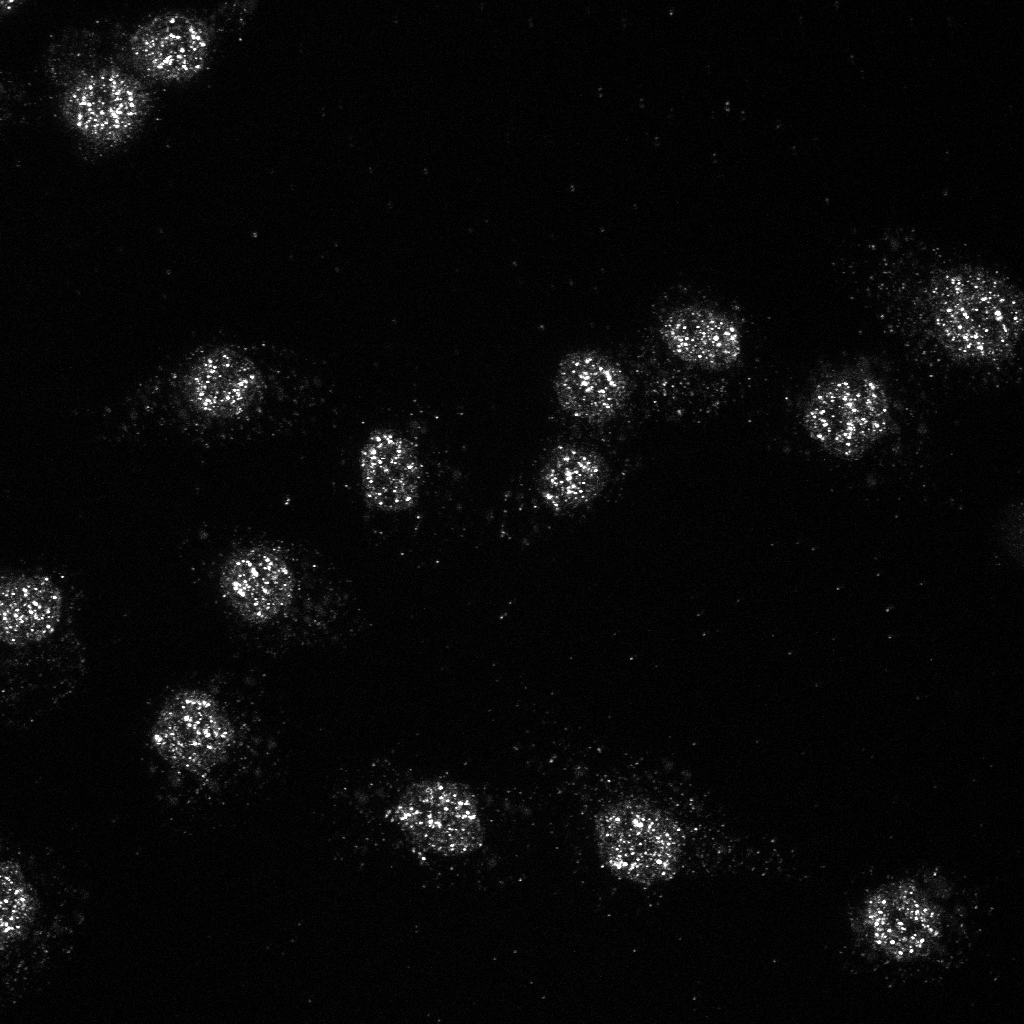

Supplement: Supplementary file 6 — Source data Fig. 6 [file 44318_2024_111_MOESM6_ESM.zip › Figure 6/Figure 6A/siGATAD2B +IR.tif]

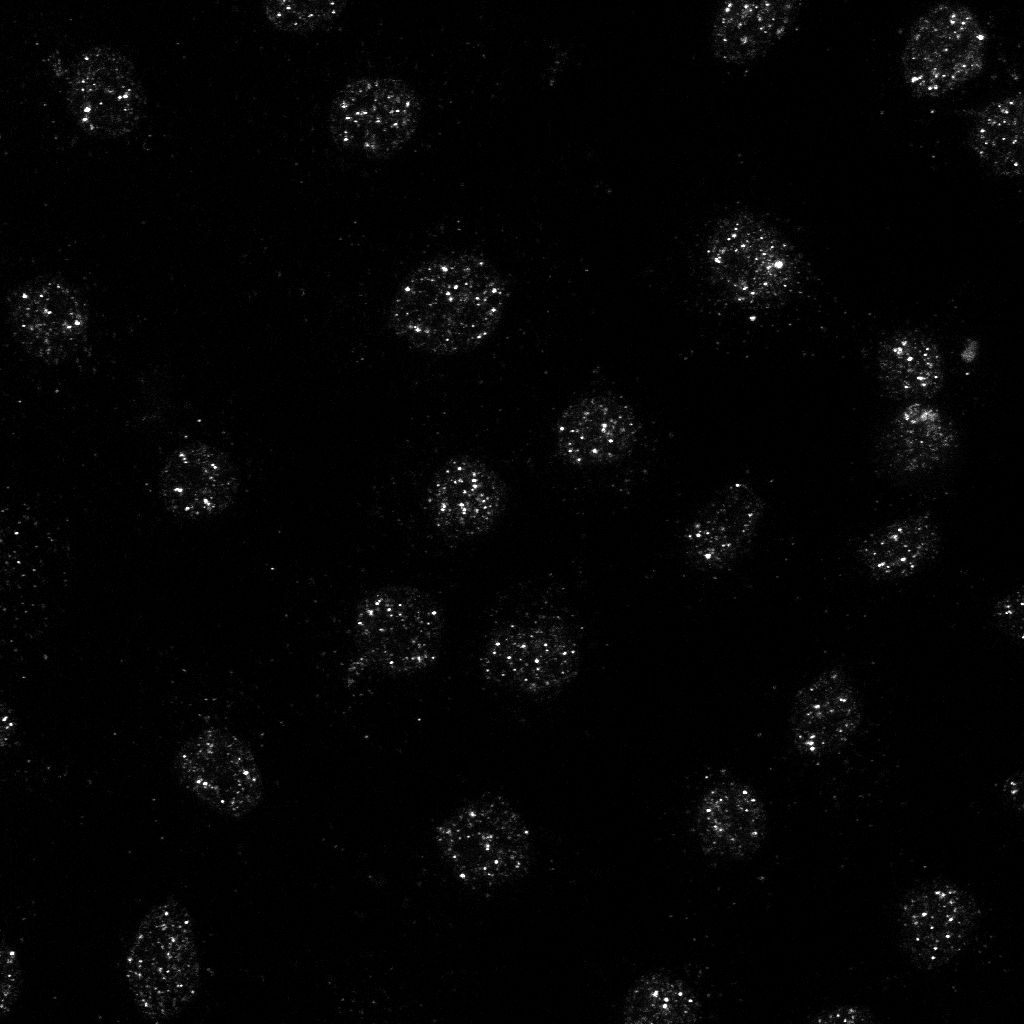

Supplement: Supplementary file 6 — Source data Fig. 6 [file 44318_2024_111_MOESM6_ESM.zip › Figure 6/Figure 6A/+IR.tif]

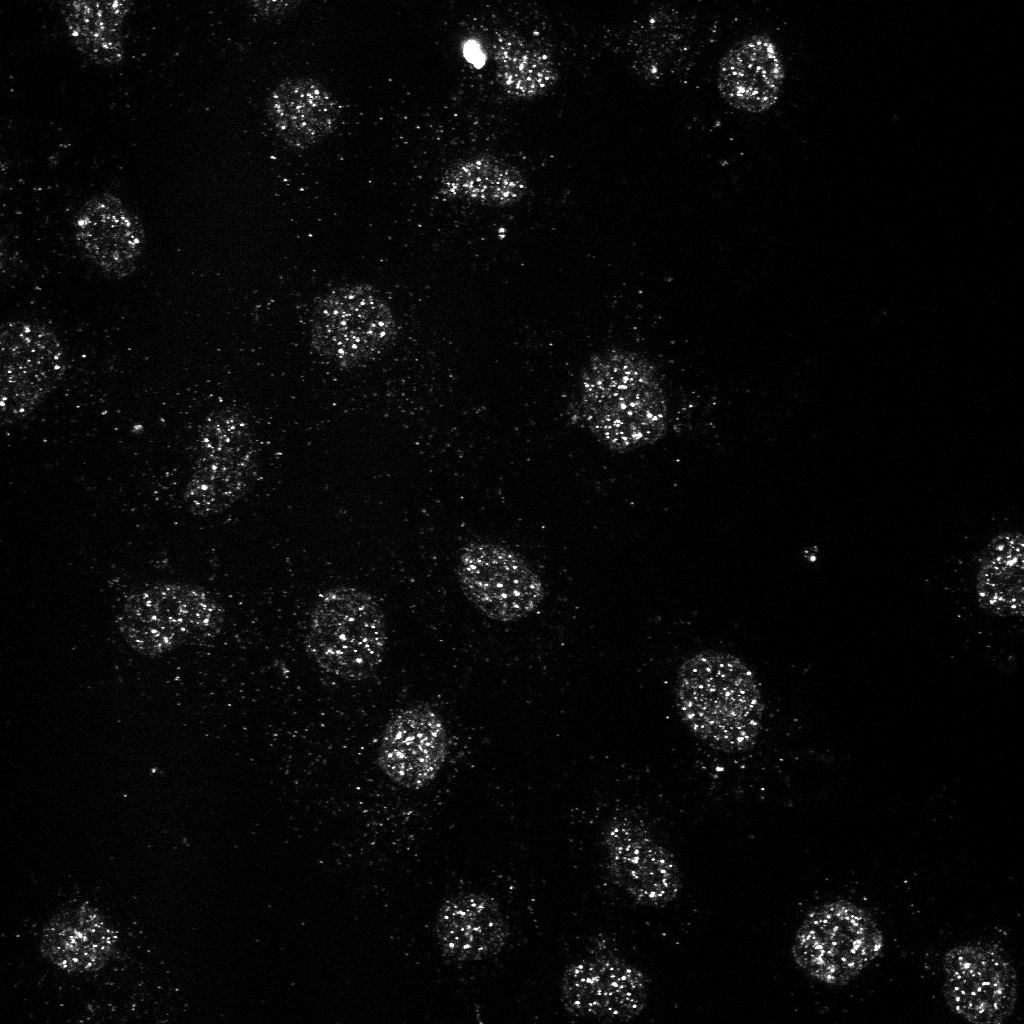

Supplement: Supplementary file 6 — Source data Fig. 6 [file 44318_2024_111_MOESM6_ESM.zip › Figure 6/Figure 6A/+IR +DRB.tif]

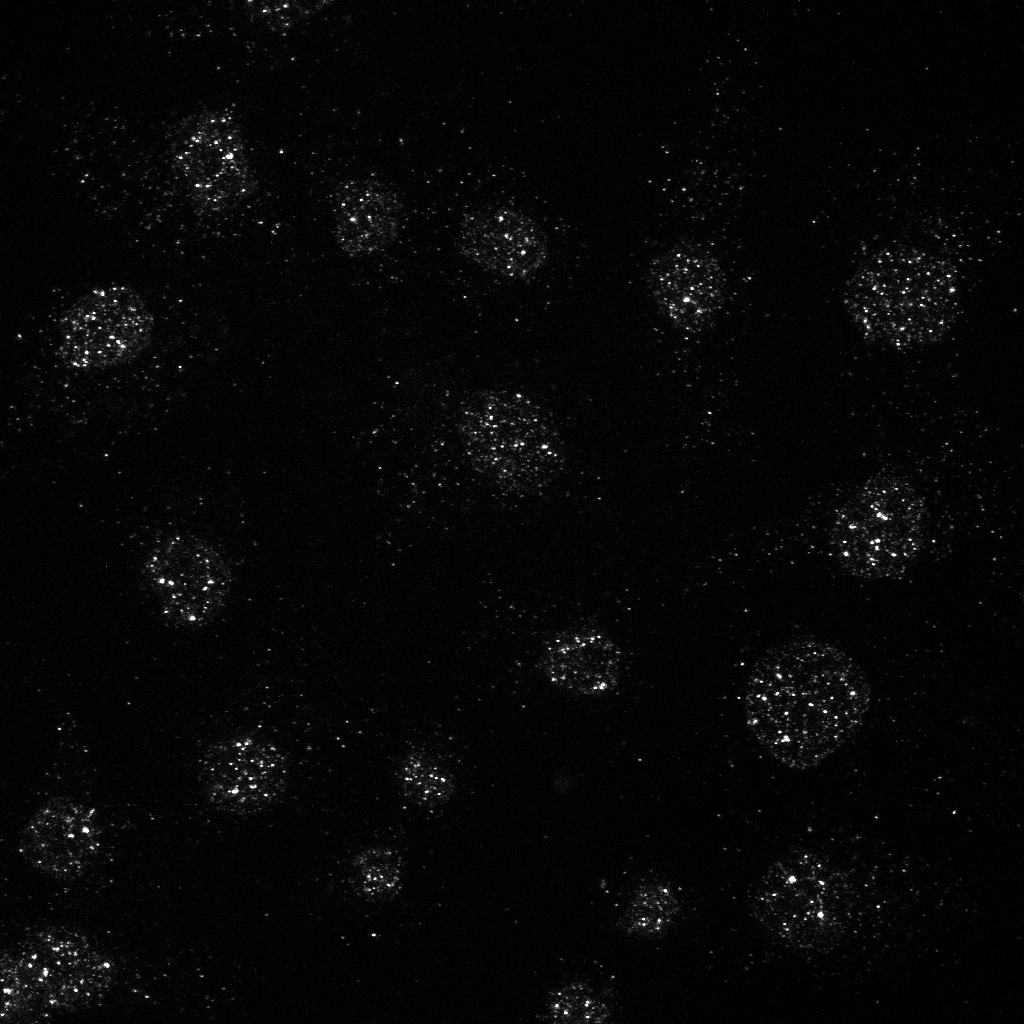

Supplement: Supplementary file 6 — Source data Fig. 6 [file 44318_2024_111_MOESM6_ESM.zip › Figure 6/Figure 6A/siMBD3 +IR.tif]

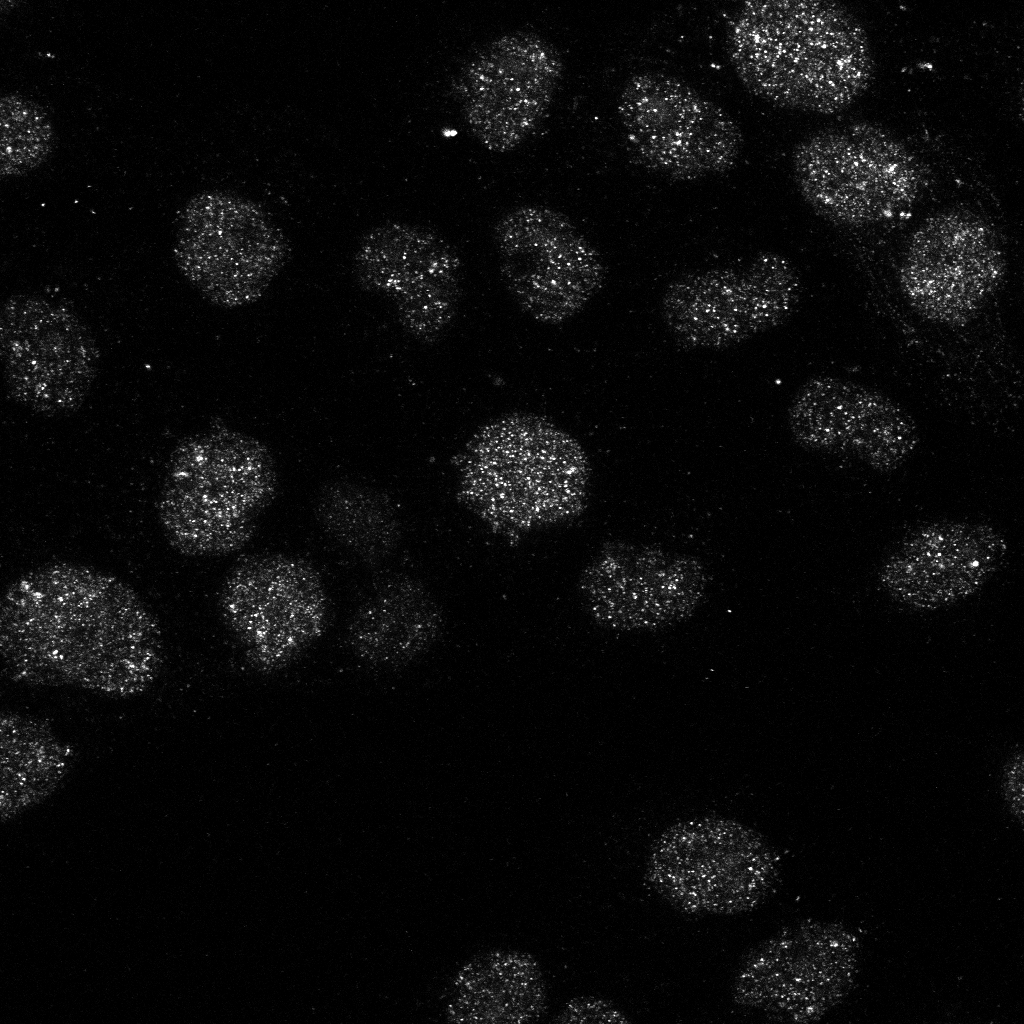

Supplement: Supplementary file 6 — Source data Fig. 6 [file 44318_2024_111_MOESM6_ESM.zip › Figure 6/Figure 6B/+IR +siMBD3.tif]

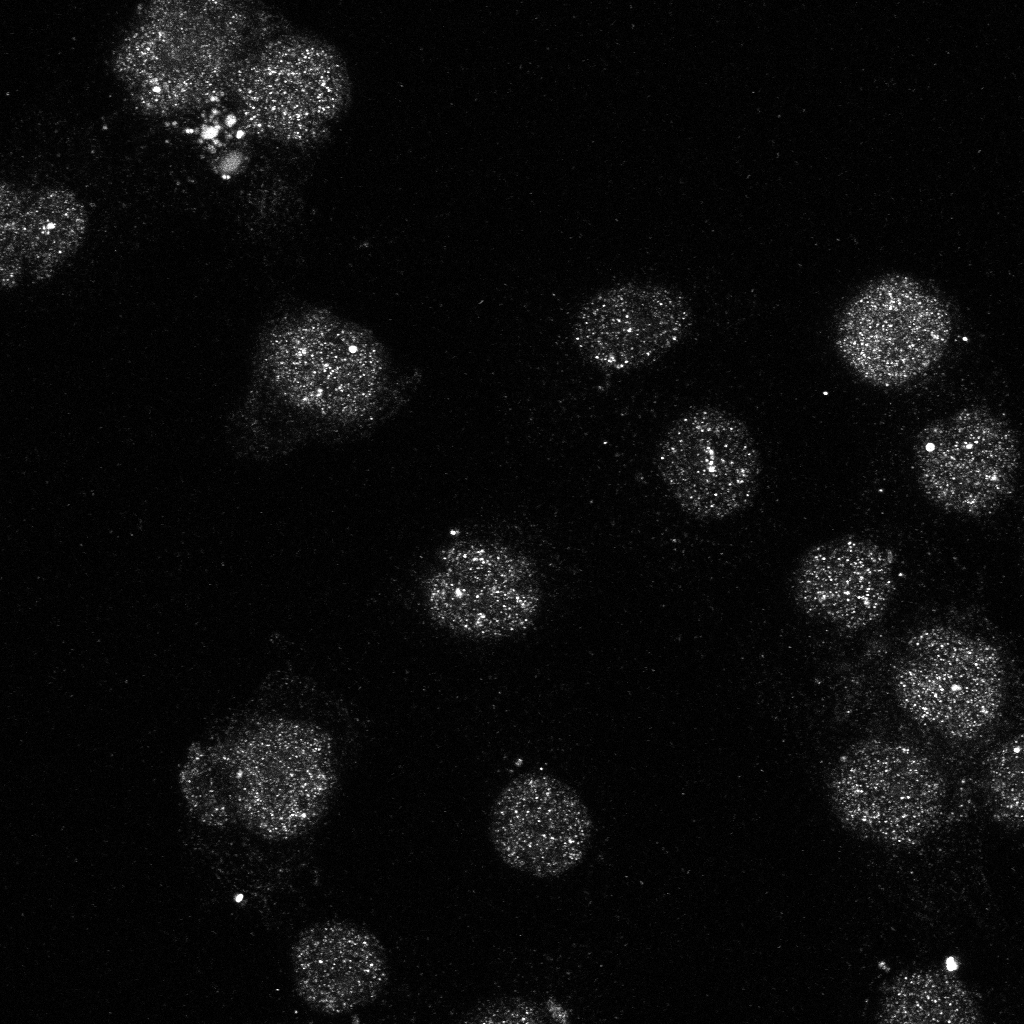

Supplement: Supplementary file 6 — Source data Fig. 6 [file 44318_2024_111_MOESM6_ESM.zip › Figure 6/Figure 6B/+IR +RNH1.tif]

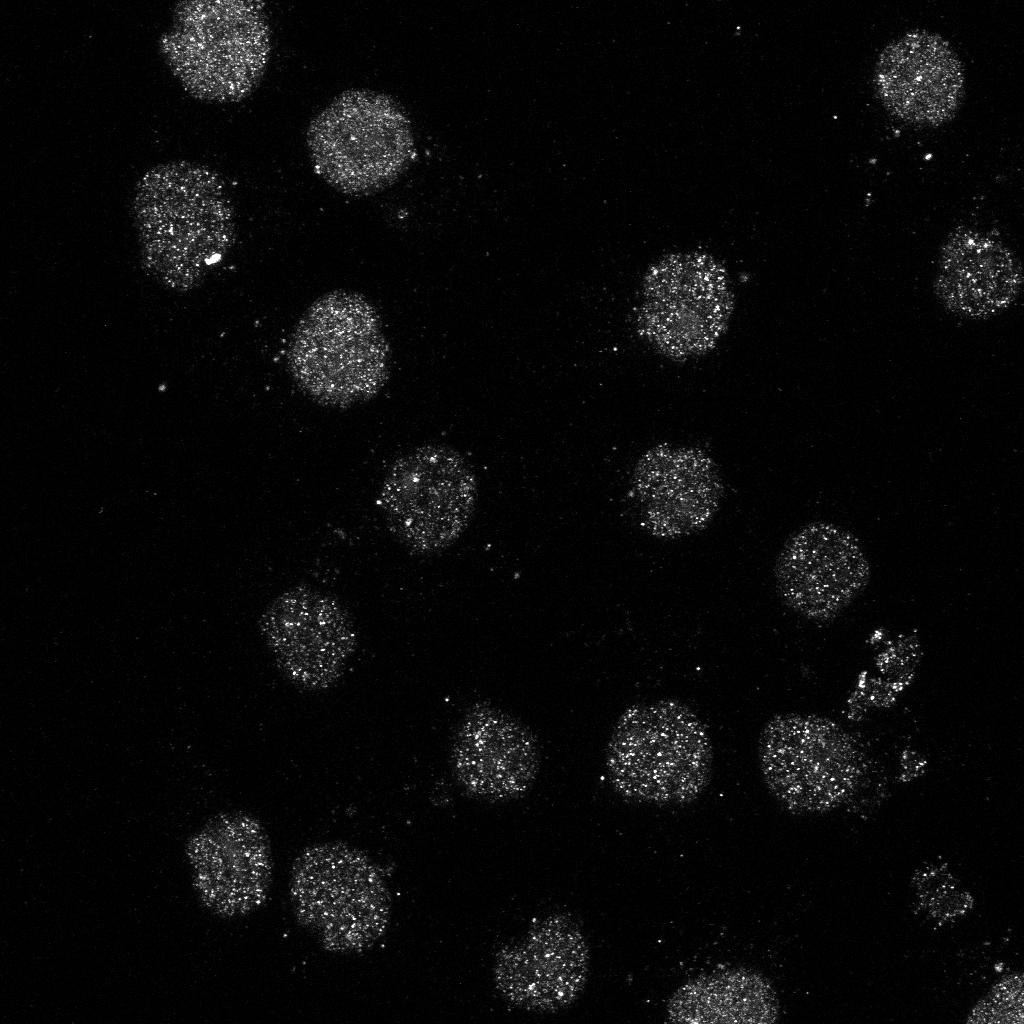

Supplement: Supplementary file 6 — Source data Fig. 6 [file 44318_2024_111_MOESM6_ESM.zip › Figure 6/Figure 6B/+IR +siGATAD2B.tif]

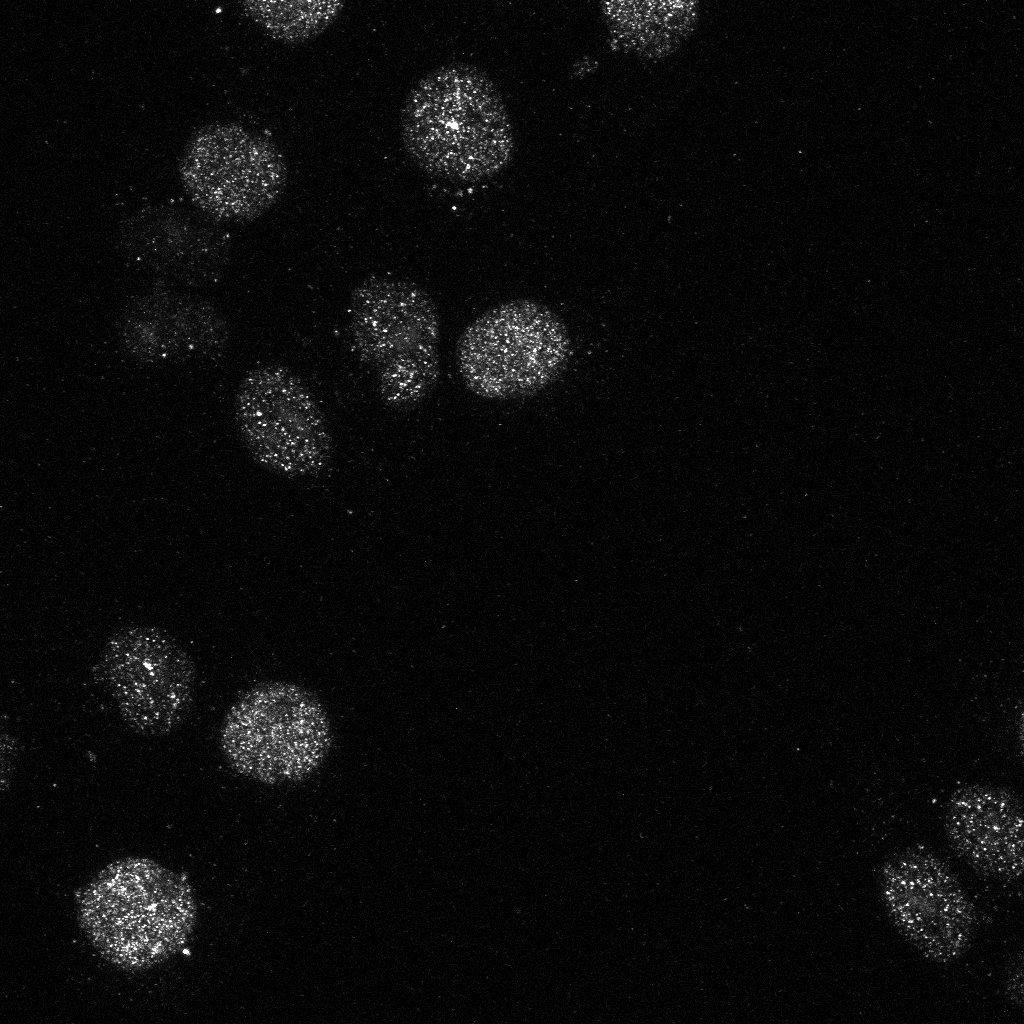

Supplement: Supplementary file 6 — Source data Fig. 6 [file 44318_2024_111_MOESM6_ESM.zip › Figure 6/Figure 6B/+IR.tif]

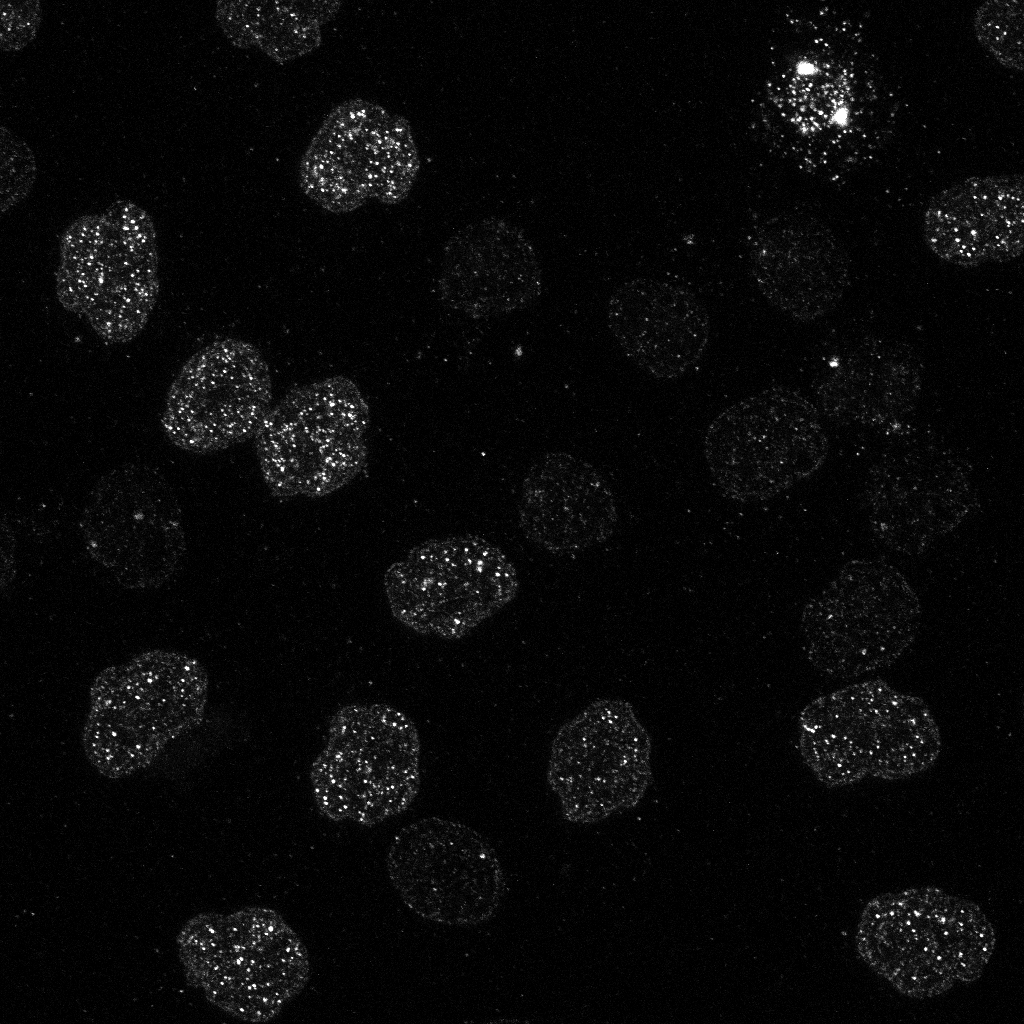

Supplement: Supplementary file 6 — Source data Fig. 6 [file 44318_2024_111_MOESM6_ESM.zip › Figure 6/Figure 6B/+IR +siBRCA1.tif]

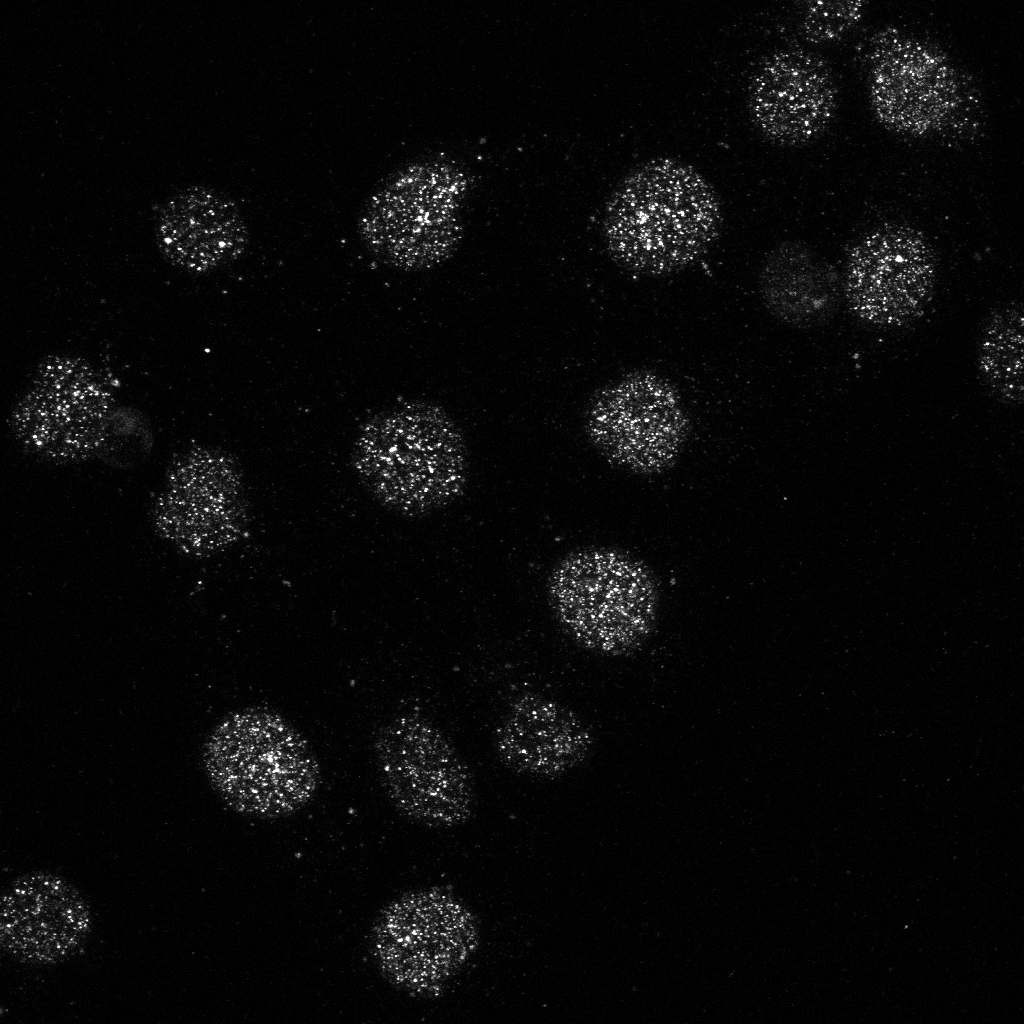

Supplement: Supplementary file 6 — Source data Fig. 6 [file 44318_2024_111_MOESM6_ESM.zip › Figure 6/Figure 6B/+IR +DRB.tif]

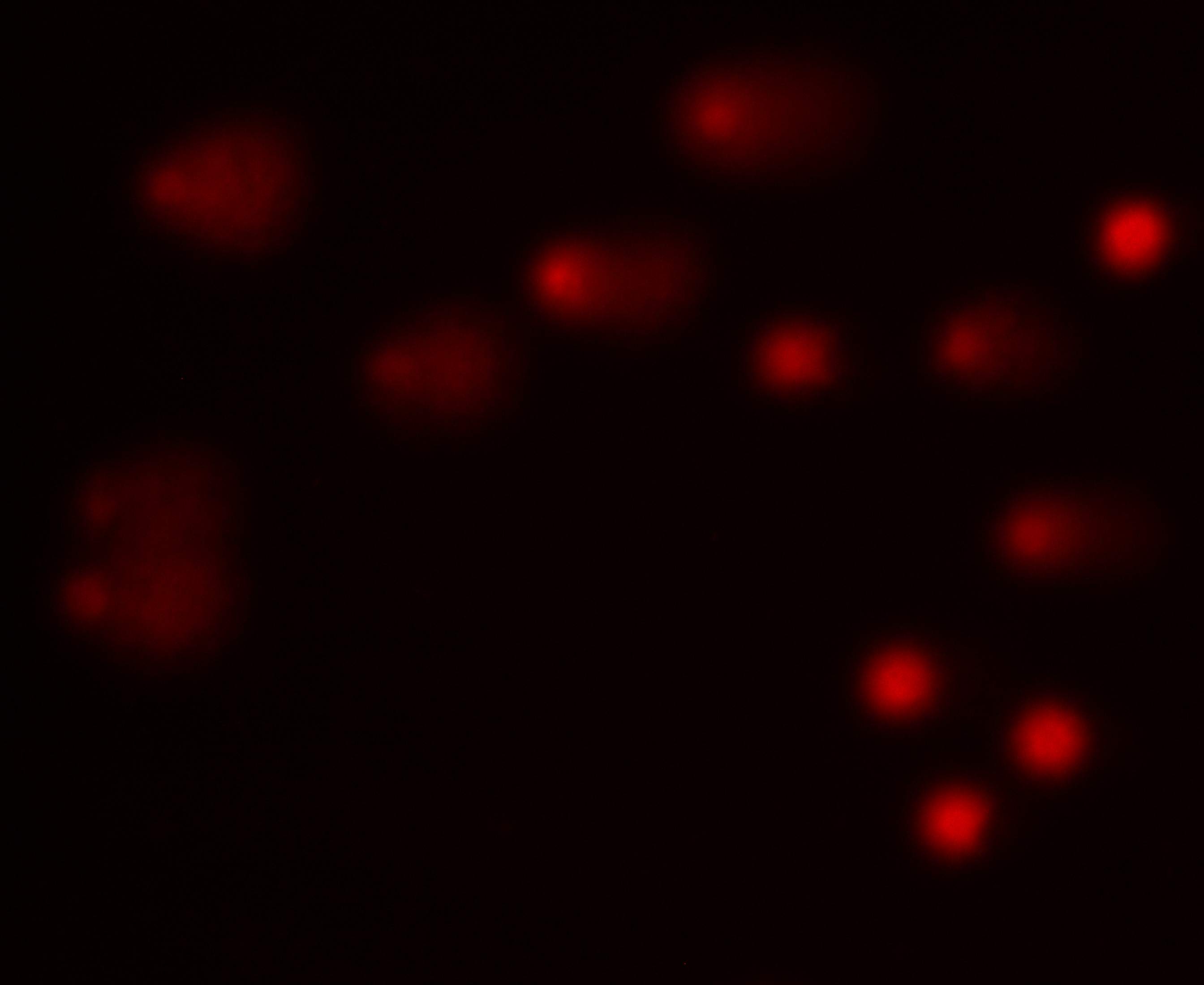

Supplement: Supplementary file 7 — Source data Fig. 7 [file 44318_2024_111_MOESM7_ESM.zip › Figure 7/Figure 7C/siGATAD2B +IR +6hr recovery.tif]

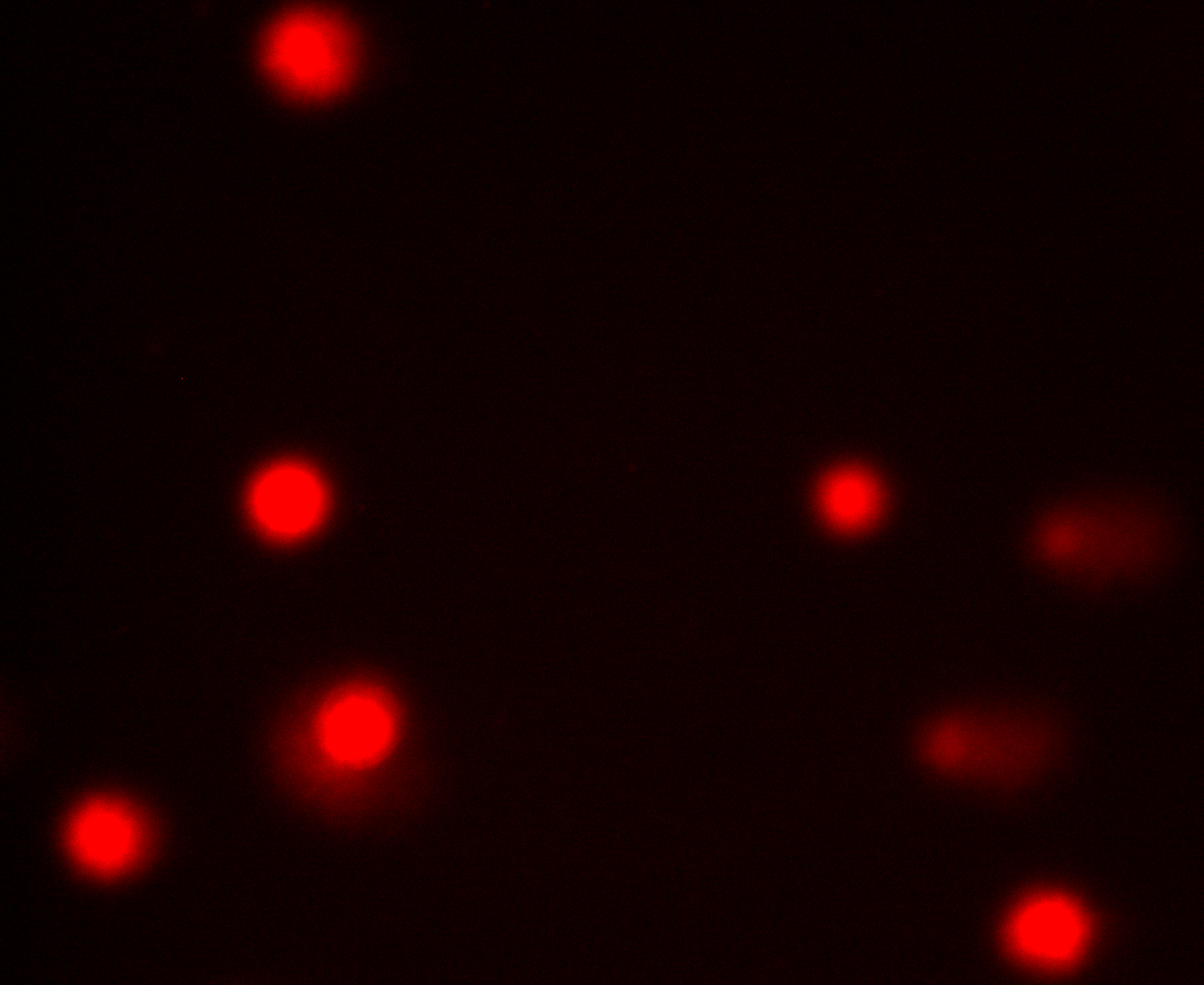

Supplement: Supplementary file 7 — Source data Fig. 7 [file 44318_2024_111_MOESM7_ESM.zip › Figure 7/Figure 7C/siMBD3 +IR +6hrs recovery.tif]

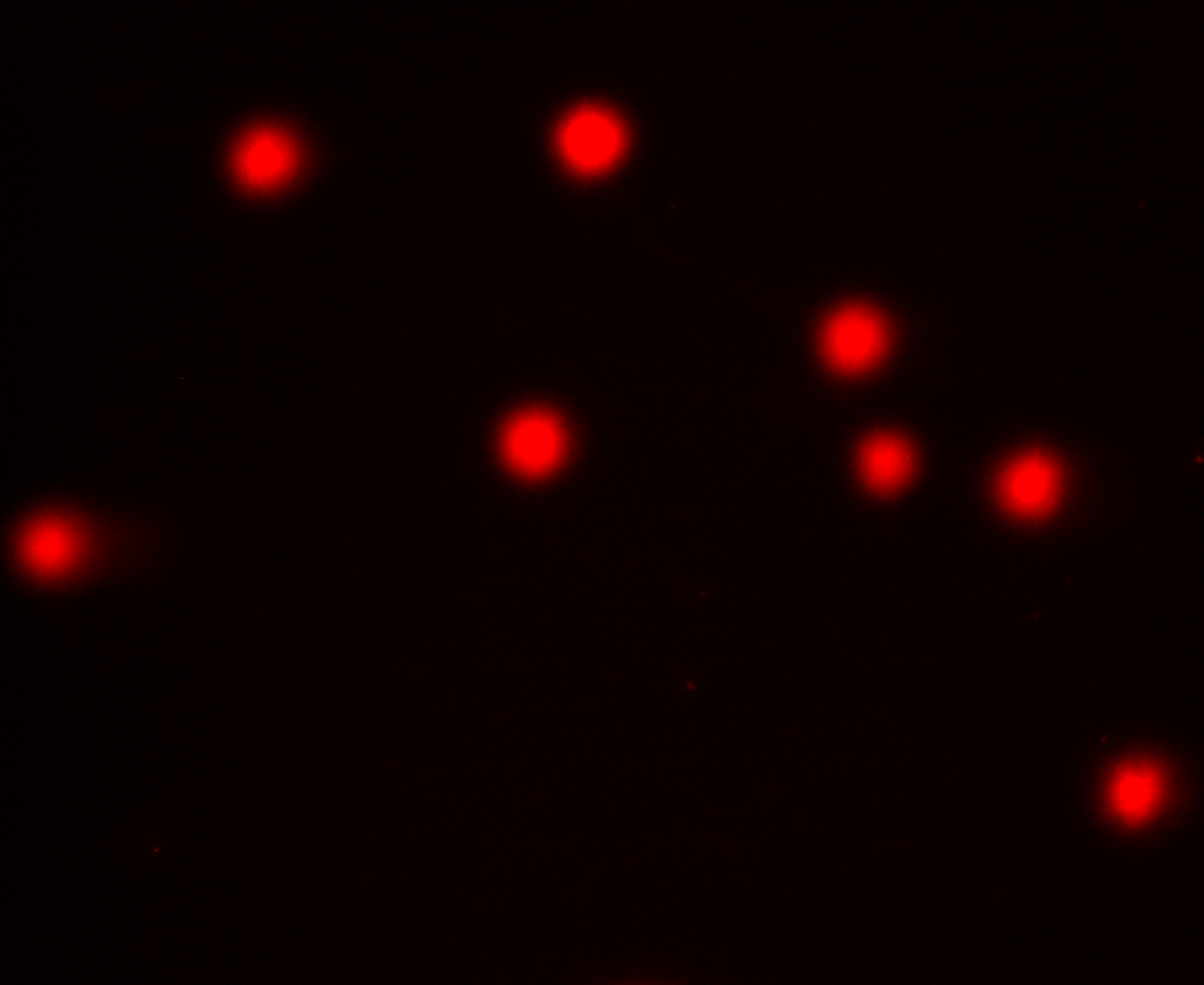

Supplement: Supplementary file 7 — Source data Fig. 7 [file 44318_2024_111_MOESM7_ESM.zip › Figure 7/Figure 7C/siNC -IR.tif]

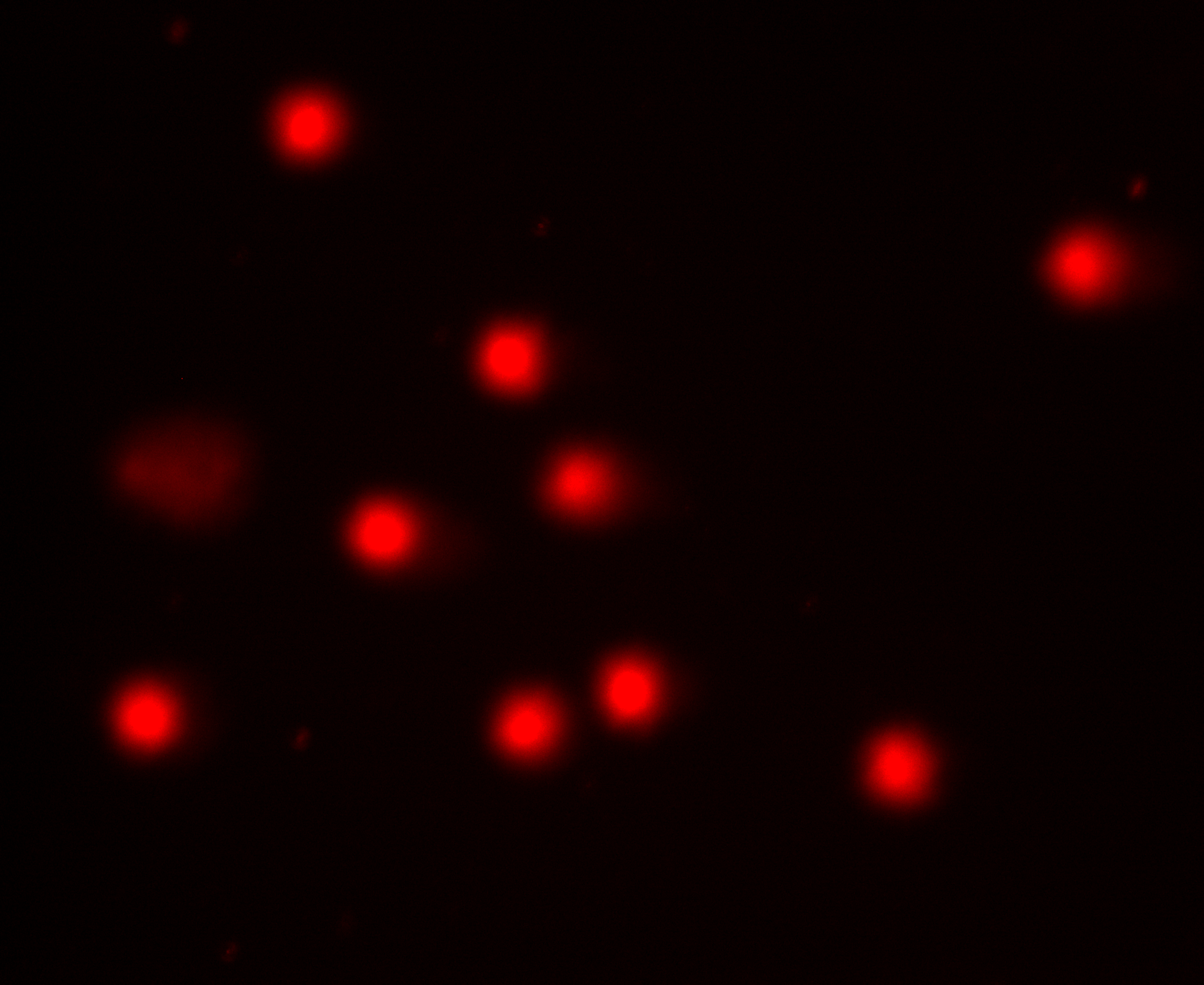

Supplement: Supplementary file 7 — Source data Fig. 7 [file 44318_2024_111_MOESM7_ESM.zip › Figure 7/Figure 7C/siGATAD2B -IR.tif]

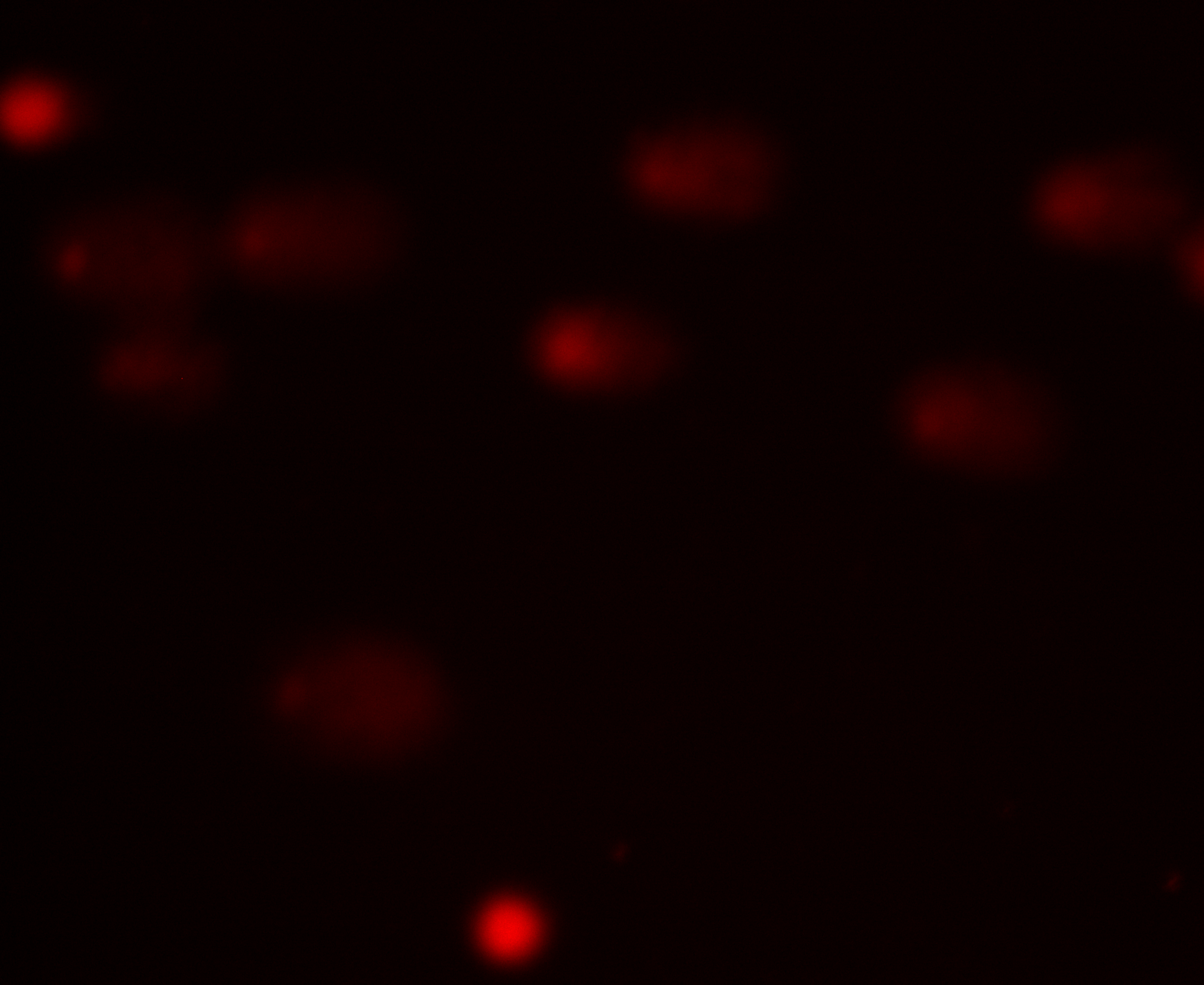

Supplement: Supplementary file 7 — Source data Fig. 7 [file 44318_2024_111_MOESM7_ESM.zip › Figure 7/Figure 7C/siGATAD2B +IR.tif]

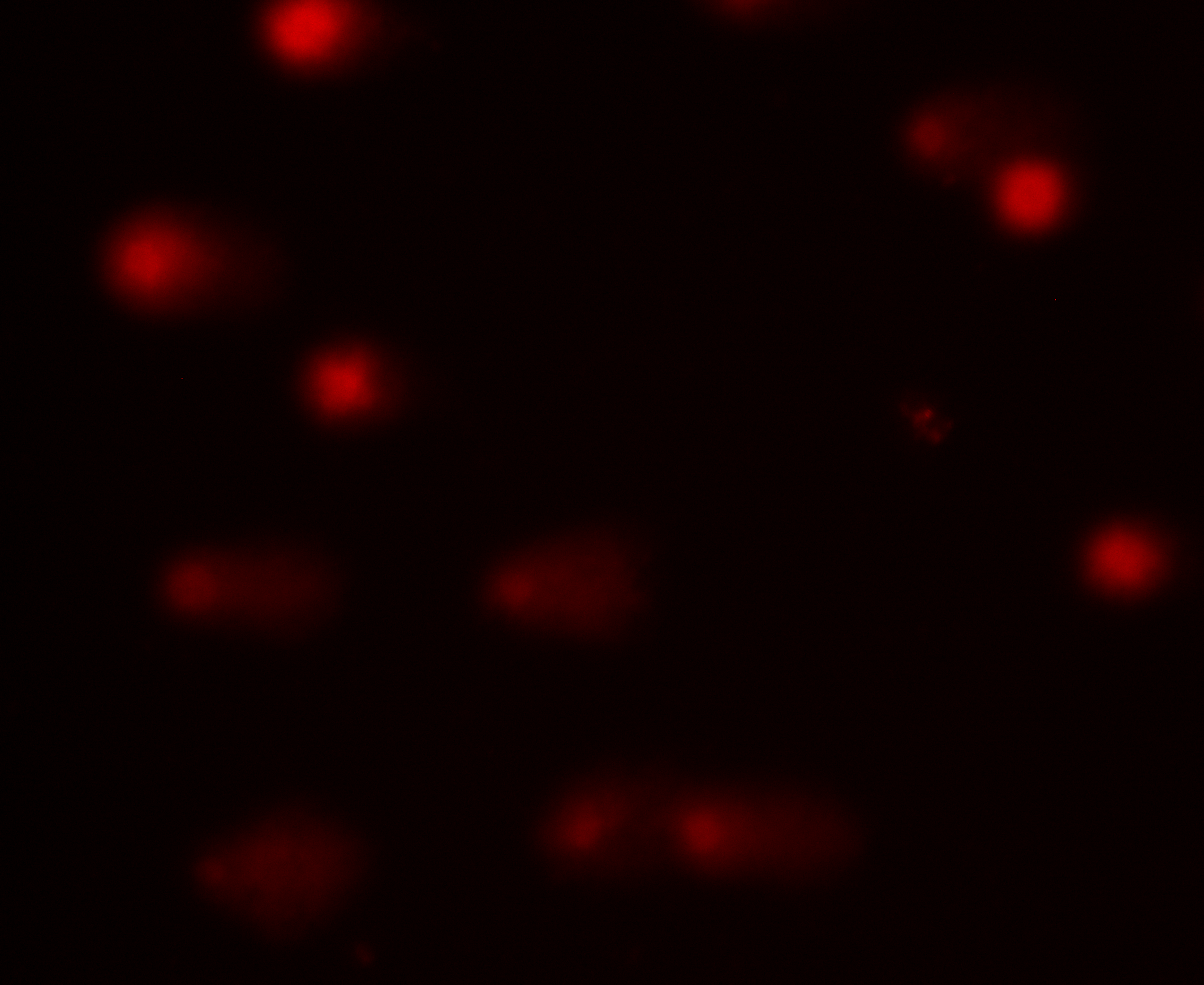

Supplement: Supplementary file 7 — Source data Fig. 7 [file 44318_2024_111_MOESM7_ESM.zip › Figure 7/Figure 7C/siNC +IR.tif]

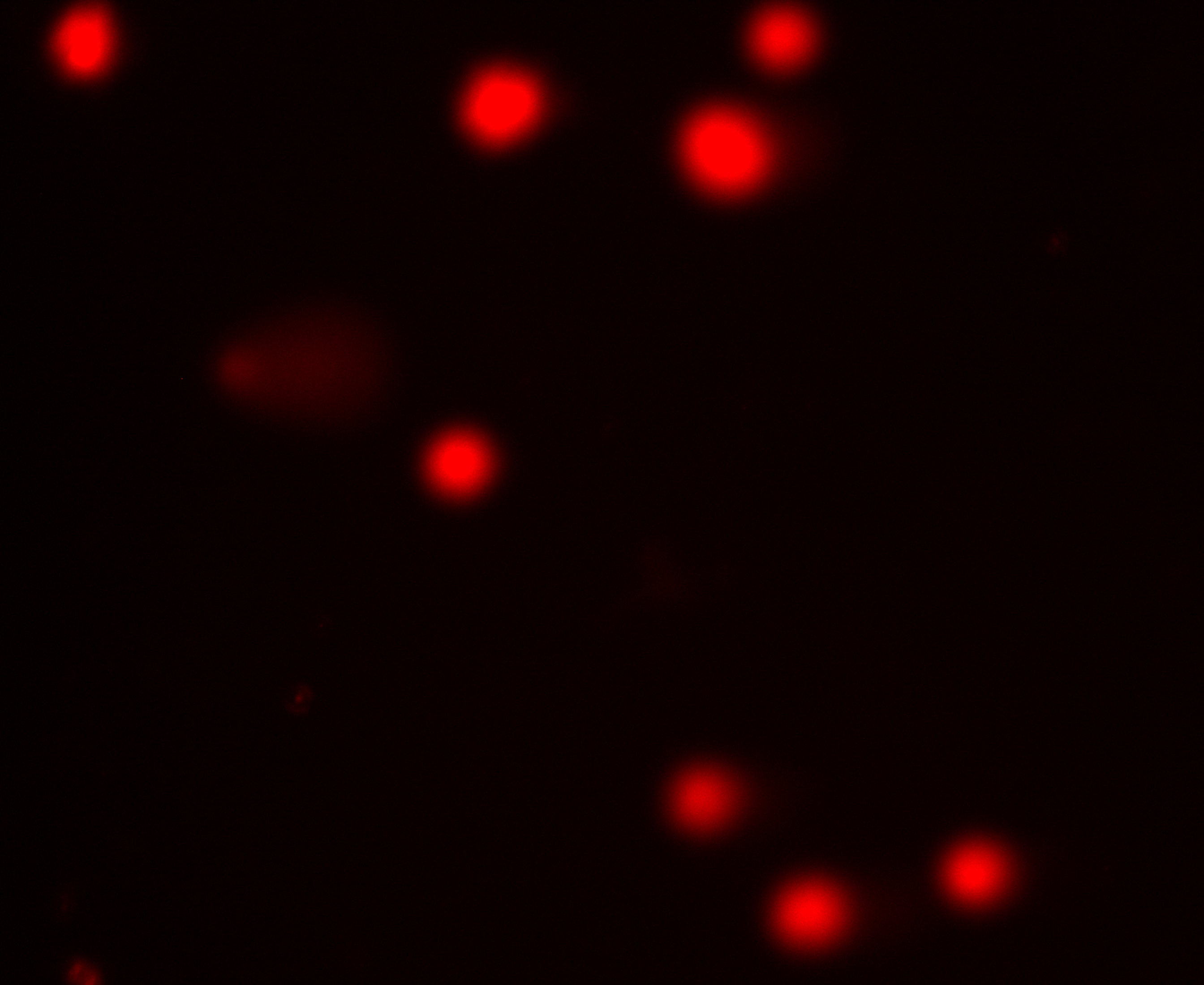

Supplement: Supplementary file 7 — Source data Fig. 7 [file 44318_2024_111_MOESM7_ESM.zip › Figure 7/Figure 7C/siNC +IR +6hrs recovery.tif]

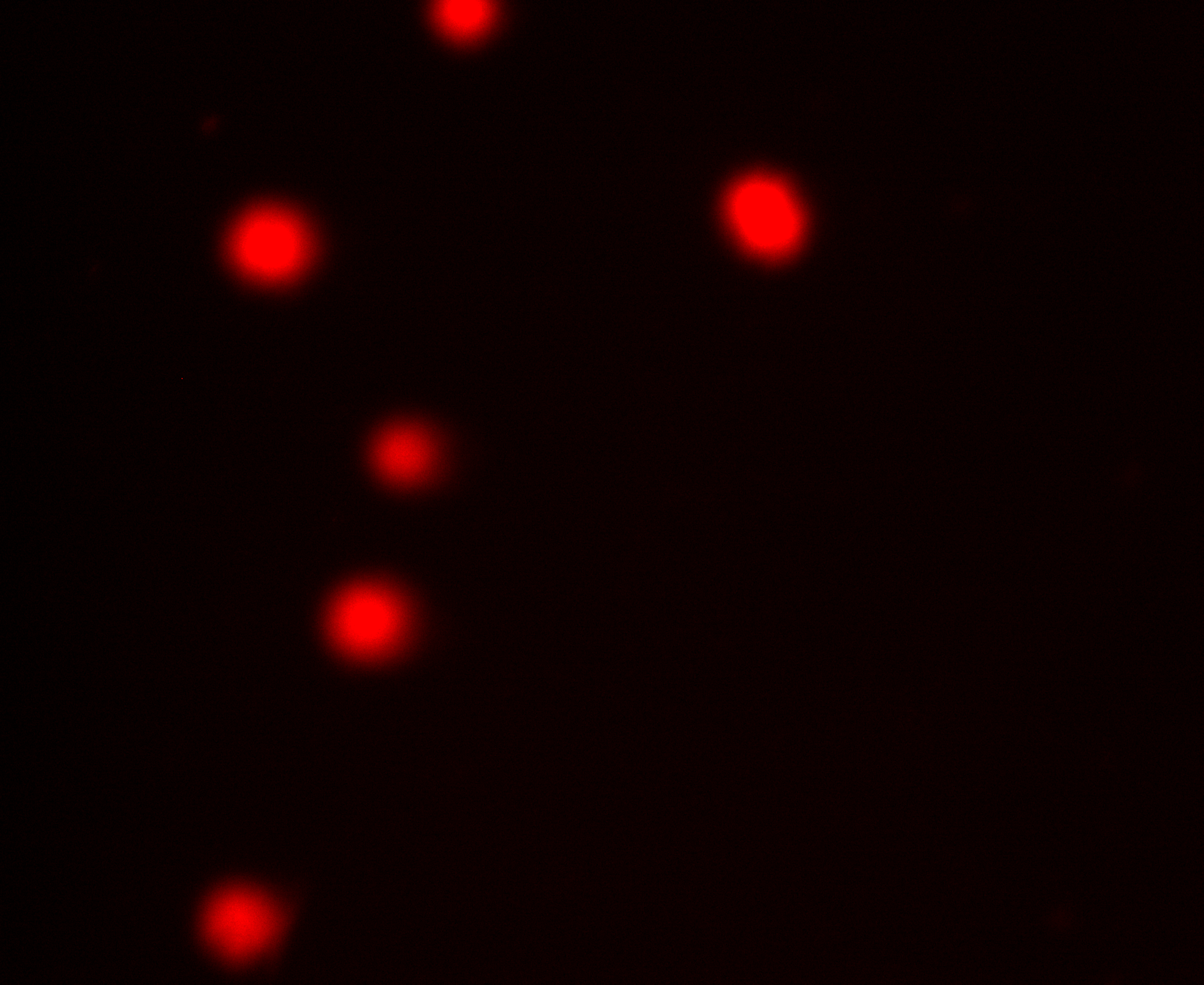

Supplement: Supplementary file 7 — Source data Fig. 7 [file 44318_2024_111_MOESM7_ESM.zip › Figure 7/Figure 7C/siMBD3 -IR.tif]

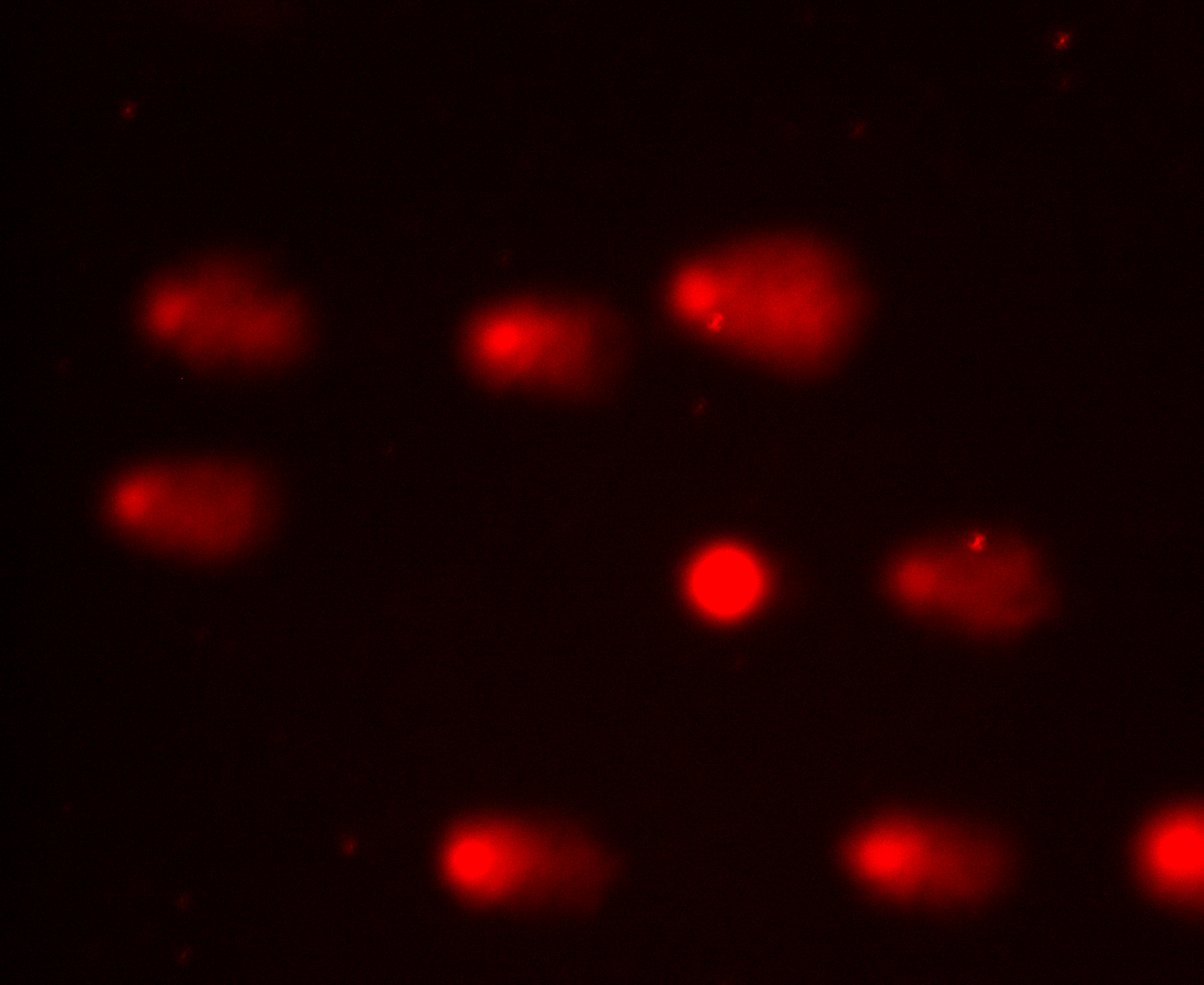

Supplement: Supplementary file 7 — Source data Fig. 7 [file 44318_2024_111_MOESM7_ESM.zip › Figure 7/Figure 7C/siMBD3 +IR.tif]
