# Supplementary material for: The GATAD2B-NuRD complex drives DNA:RNA hybrid-dependent chromatin boundary formation upon DNA damage
Source: EMBO J. 2024 May 8;43(12):8. doi: 10.1038/s44318-024-00111-7 (PMC11183058; doi:10.1038/s44318-024-00111-7)
Supplement: Supplementary file 9 — Expanded View Figures [file 44318_2024_111_MOESM9_ESM.pdf]

# Expanded View Figures

**Figure EV1. The GATAD2B-NuRD complex binds to DNA:RNA hybrids upon DNA damage.**

(**A**) Representative confocal images showing single antibody controls for PLA shown in Fig. 1. Scale bar = 10 μm. (**B**) Representative confocal images from three independent replicates showing PLA of HDAC1 and S9.6 in cells with or without IR and overexpression of RNAseH1 Left: representative confocal microscopy images and single antibody PLA control; right: quantification of left, error bar = mean ± SEM, significance was determined using nonparametric Mann–Whitney test. ****$P \leq 0.0001$. Scale bar = 10 μm, $n > 50$ cells from three biological repeats. (**C**) Representative confocal images from three independent replicates showing immunofluorescence of γH2AX staining upon laser-induced DNA damage. Cells were fixed ~2 min after irradiation of laser. Nuclei were stained by DAPI. Scale bar = 10 μm, $n > 3$. (**D**) Laser stripping of MBD3-GFP cells with or without treatment with transcription inhibitors triptolide (TPL3) and DRB. Representative spinning disk confocal microscopy images from three independent replicates and quantification ($n \geq 10$) showing GFP signals before and after laser striping at indicated time points; error bar = mean ± SEM. Scale bar = 10 μm, $n > 3$. (**E**) Laser stripping of MBD3-GFP cells with or without transiently expression of RNAseH1-RFP plasmid. Representative spinning disk confocal microscopy images from three independent replicates and quantification ($n \geq 10$) showing GFP and RFP signals before and after laser striping at indicated time points; error bar = mean ± SEM. Scale bar = 10 μm, $n > 3$. (**F**) Quantification ($n \geq 10$) showing GATAD2B-GFP and RNH1-mCherry signals before and after laser striping at indicated time points; error bar = mean ± SEM. $n = 3$ biological repeats. (**G**) ChIP-qPCR bar charts showing levels of HDAC1 (left) and GATAD2B (right) at three genes known to be bound by NuRD complex in non-damage condition in cells treated with Triptolide or overexpressing RNaseH1, $n = 3$, error bar = mean ± SEM. (**H**) Representative confocal images from three independent replicates showing PLA of MBD3 and γH2AX in cells with or without IR followed by 20 min recovery, and transcription inhibition (TLP3 or DRB) or overexpression of RNAseH1. IR = 5 Gy. Left: representative confocal microscopy images; right: quantification of left, error bar = mean ± SEM, significance was determined using nonparametric Mann–Whitney test. ****$P \leq 0.0001$. Scale bar = 10 μm, $n > 3$.

 © The Author(s)

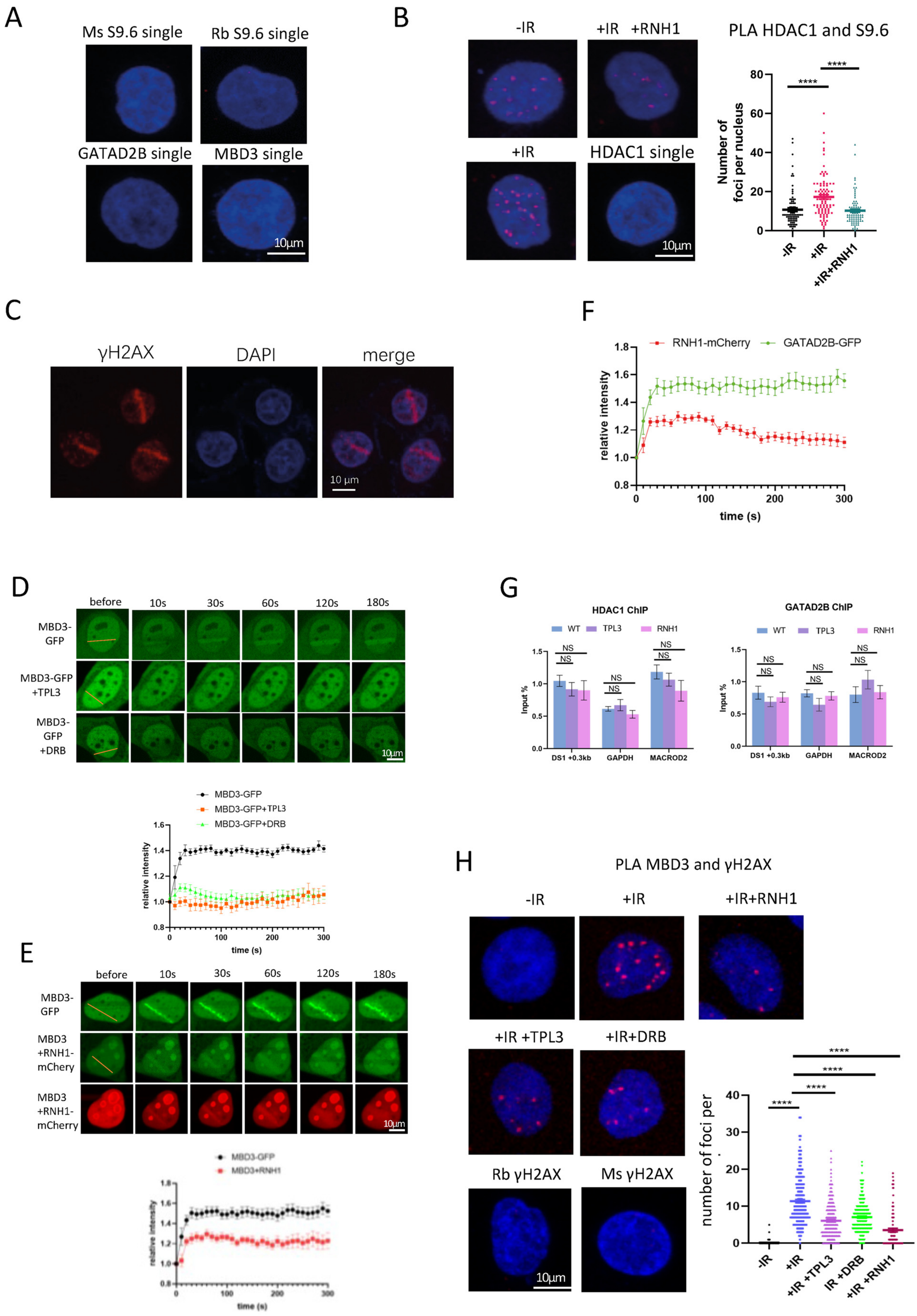
A
Ms S9.6 single
Rb S9.6 single
GATAD2B single
MBD3 single
10µm
B
-IR
+IR +RNH1
+IR
HDAC1 single
10µm
PLA HDAC1 and S9.6
Number of foci per nucleus
****
****
-IR
+IR
+IR+RNH1
C
γH2AX
DAPI
merge
10 µm
F
RNH1-mCherry
GATAD2B-GFP
relative intensity
time (s)
D
before
10s
30s
60s
120s
180s
MBD3-GFP
MBD3-GFP +TPL3
MBD3-GFP +DRB
10µm
MBD3-GFP
MBD3-GFP+TPL3
MBD3-GFP+DRB
relative intensity
time (s)
G
HDAC1 ChIP
GATAD2B ChIP
WT
TPL3
RNH1
NS
Input %
DS1 +0.3kb
GAPDH
MACROD2
E
before
10s
30s
60s
120s
180s
MBD3-GFP
MBD3 +RNH1-mCherry
MBD3 +RNH1-mCherry
10µm
MBD3-GFP
MBD3+RNH1
relative intensity
time (s)
H
PLA MBD3 and γH2AX
-IR
+IR
+IR+RNH1
+IR +TPL3
+IR+DRB
Rb γH2AX
Ms γH2AX
10µm
number of foci per
****
-IR
+IR
+IR +TPL3
IR +DRB
+IR +RNH1

© The Author(s)

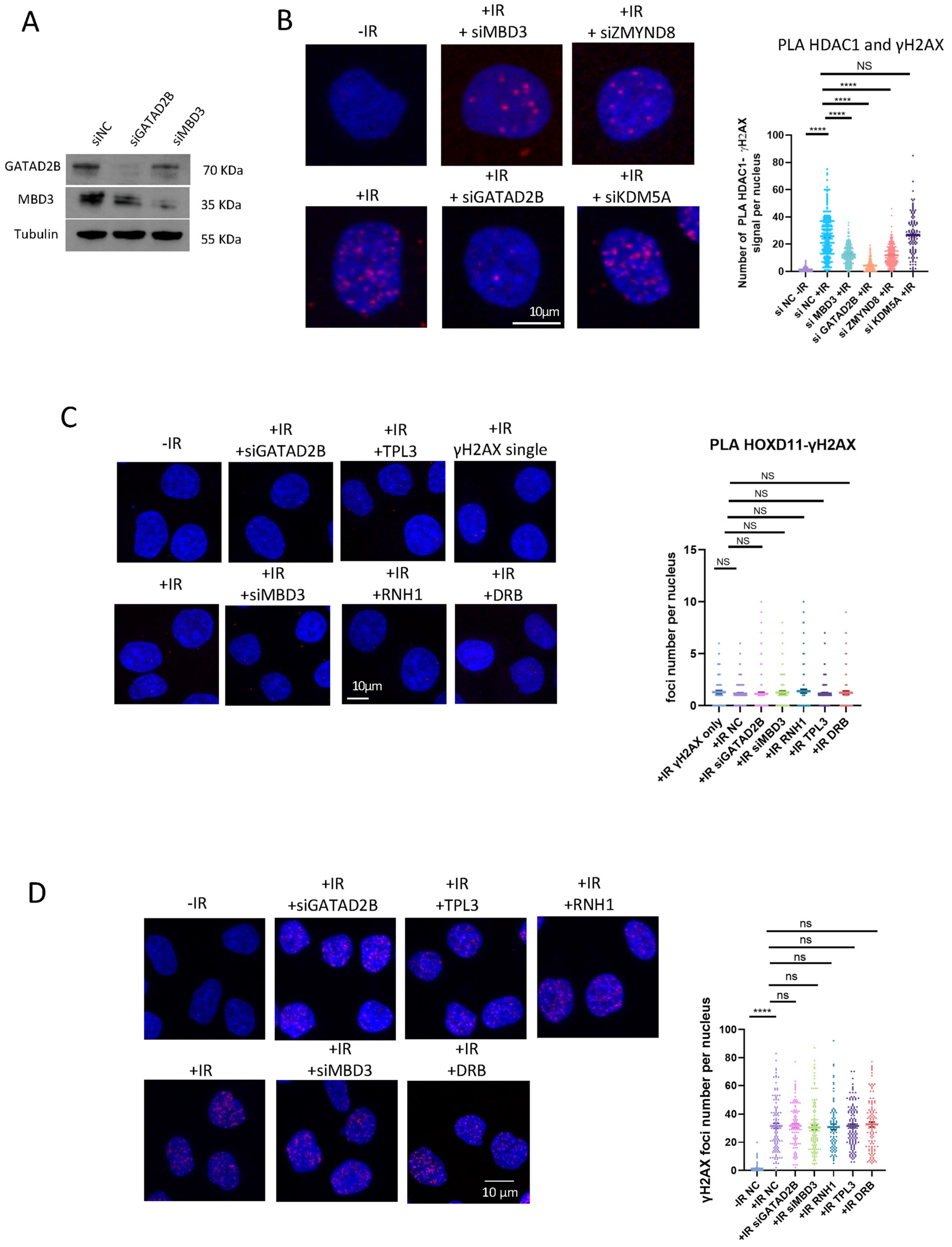
A
siNC
siGATAD2B
siMBD3
GATAD2B
MBD3
Tubulin
70 KDa
35 KDa
55 KDa
B
-IR
+IR + siMBD3
+IR + siZMYND8
+IR
+IR + siGATAD2B
+IR + siKDM5A
10µm
PLA HDAC1 and γH2AX
Number of PLA HDAC1- γH2AX signal per nucleus
NS
****
si NC -IR
si NC +IR
si MBD3 +IR
si GATAD2B +IR
si ZMYND8 +IR
si KDM5A +IR
C
-IR
+IR +siGATAD2B
+IR +TPL3
+IR γH2AX single
+IR
+IR +siMBD3
+IR +RNH1
+IR +DRB
10µm
PLA HOXD11-γH2AX
foci number per nucleus
NS
+IR γH2AX only
+IR NC
+IR siGATAD2B
+IR siMBD3
+IR RNH1
+IR TPL3
+IR DRB
D
-IR
+IR +siGATAD2B
+IR +TPL3
+IR +RNH1
+IR
+IR +siMBD3
+IR +DRB
10 µm
γH2AX foci number per nucleus
ns
****
-IR NC
+IR NC
+IR siGATAD2B
+IR siMBD3
+IR RNH1
+IR TPL3
+IR DRB

 © The Author(s)

**Figure EV2. The GATAD2B-NuRD complex specifically localises to DSBs.**

(**A**) Western blot showing efficiency of siRNA mediated knockdown of GATAD2B and MBD3, NC negative control. Tubulin was used as loading control.
(**B**) Representative confocal images from three independent replicates showing PLA of HDAC1 and γH2AX in cells with or without IR and depleted of ZMYND8 or KDM5A, IR = 5 Gy. Left: representative confocal microscopy images; right: quantification of left, error bar = mean ± SEM, significance was determined using nonparametric Mann–Whitney test. ****$P \le 0.0001$. Scale bar = 10 μm, $n > 50$ cells from 3 biological repeats. (**C**) Representative confocal images from three independent replicates showing PLA of HOXD11 and γH2AX in cells with or without IR followed by 20 min recovery, and transcription inhibition (TLP3 or DRB), overexpression of RNAseH1 or depletion of GATAD2B and MBD3, IR = 5 Gy. Left: representative confocal microscopy images; right: quantification of left, error bar = mean ± SEM, significance was determined using nonparametric Mann–Whitney test. ****$P \le 0.0001$. Scale bar = 10 μm, $n > 50$ cells from three biological repeats. (**D**) Immunofluorescence of γH2AX in cells with 5 Gy IR followed by 20 min recovery, and transcription inhibition (TLP3 or DRB), overexpression of RNAseH1 or depletion of GATAD2B and MBD3. Left: representative confocal microscopy images; right: quantification of left, error bar = mean ± SEM, significance was determined using nonparametric Mann–Whitney test. ****$P \le 0.0001$, NS no significance. Scale bar = 10 μm, $n > 50$ cells from three biological repeats.

© The Author(s)

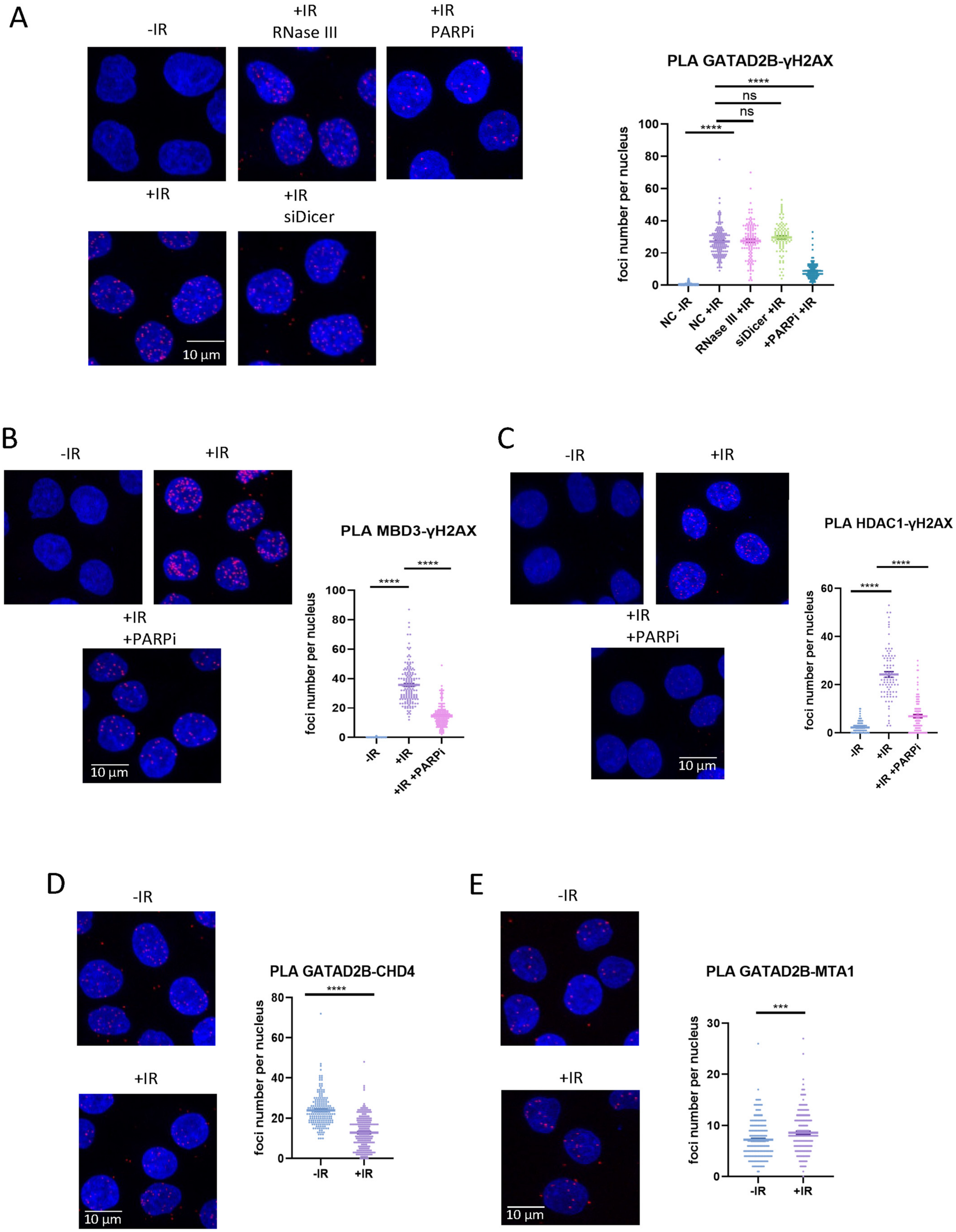
A
-IR
+IR RNase III
+IR PARPi
+IR
+IR siDicer
10 μm
PLA GATAD2B-γH2AX
****
ns
ns
****
foci number per nucleus
NC -IR
NC +IR
RNase III +IR
siDicer +IR
+PARPi +IR
B
-IR
+IR
+IR +PARPi
10 μm
PLA MBD3-γH2AX
****
****
foci number per nucleus
-IR
+IR
+IR +PARPi
C
-IR
+IR
+IR +PARPi
10 μm
PLA HDAC1-γH2AX
****
****
foci number per nucleus
-IR
+IR
+IR +PARPi
D
-IR
+IR
10 μm
PLA GATAD2B-CHD4
****
foci number per nucleus
-IR
+IR
E
-IR
+IR
10 μm
PLA GATAD2B-MTA1
***
foci number per nucleus
-IR
+IR

 © The Author(s)

**Figure EV3. The GATAD2B-NuRD localises to DSBs in PARP1-dependent manner.**

(**A**) PLA of GATAD2B and γH2AX in cells with or without IR treatment followed by 20 min recovery and treatment of the PARP inhibitor or RNase III or knockdown of Dicer. IR = 5 Gy. Left: representative confocal microscopy images from three independent replicates; right: quantification of left, error bar = mean ± SEM, significance was determined using nonparametric Mann–Whitney test. ****$P \le 0.0001$. Scale bar = 10 μm, $n > 50$ cells from three biological repeats. (**B**) PLA of MBD3 and γH2AX in cells with or without IR treatment followed by 20 min recovery and treatment of the PARP inhibitor. IR = 5 Gy. Left: representative confocal microscopy images from three independent replicates; right: quantification of left, error bar = mean ± SEM, significance was determined using nonparametric Mann–Whitney test. ****$P \le 0.0001$. Scale bar = 10 μm, $n > 50$ cells from three biological repeats. (**C**) PLA of HDAC1 and γH2AX in cells with or without IR treatment followed by 20 min recovery and treatment of the PARP inhibitor. IR = 5 Gy. Left: representative confocal microscopy images from three independent replicates; right: quantification of left, error bar = mean ± SEM, significance was determined using nonparametric Mann–Whitney test. ****$P \le 0.0001$. Scale bar = 10 μm, $n > 50$ cells from three biological repeats. (**D**) PLA of GATAD2B and CHD4 in cells with or without IR treatment followed by 20 min recovery. IR = 5 Gy. Left: representative confocal microscopy images from three independent replicates; right: quantification of left, error bar = mean ± SEM, significance was determined using nonparametric Mann–Whitney test. ****$P \le 0.0001$. Scale bar = 10 μm, $n > 50$ cells from 3 biological repeats. (**E**) PLA of GATAD2B and MTA1 in cells with or without IR treatment followed by 20 min recovery. IR = 5 Gy. Left: representative confocal microscopy images from three independent replicates; right: quantification of left, error bar = mean ± SEM, significance was determined using nonparametric Mann–Whitney test. ***$P \le 0.001$. Scale bar = 10 μm, $n > 50$ cells from three biological repeats.

© The Author(s)

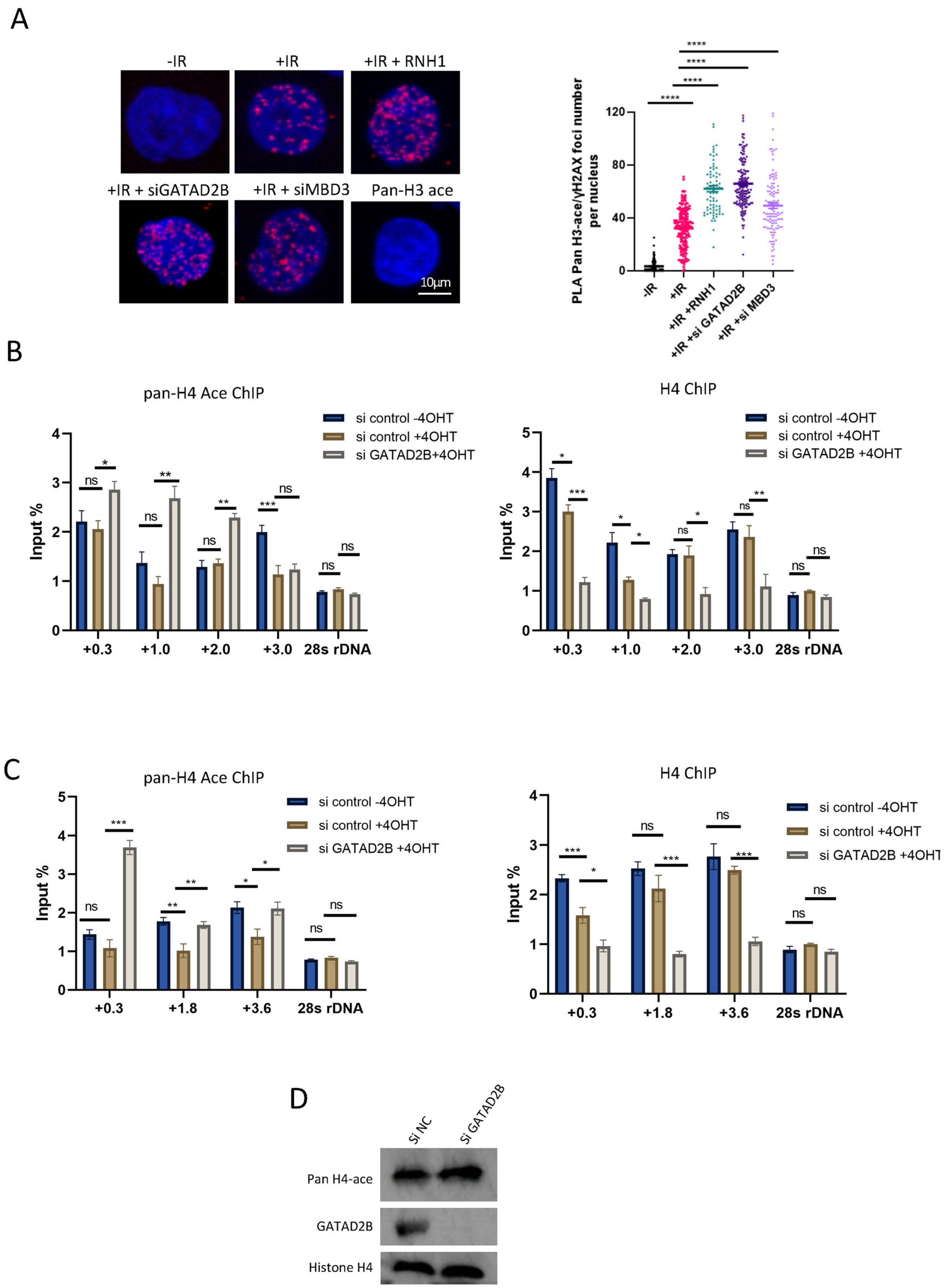
A
-IR
+IR
+IR + RNH1
+IR + siGATAD2B
+IR + siMBD3
Pan-H3 ace
10μm
PLA Pan H3-ace/γH2AX foci number per nucleus
0
40
80
120
****
****
****
****
-IR
+IR
+IR +RNH1
+IR +si GATAD2B
+IR +si MBD3
B
pan-H4 Ace ChIP
si control -4OHT
si control +4OHT
si GATAD2B+4OHT
Input %
+0.3
+1.0
+2.0
+3.0
28s rDNA
ns
*
**
***
H4 ChIP
si GATAD2B +4OHT
C
pan-H4 Ace ChIP
+0.3
+1.8
+3.6
28s rDNA
H4 ChIP
D
Si NC
Si GATAD2B
Pan H4-ace
GATAD2B
Histone H4

 © The Author(s)

**Figure EV4. The lack of GATAD2B-NuRD complex leads to histone hyper-acetylation at DSBs.**

(**A**) PLA of pan-acetyl H3 and γH2AX in cells with or without IR followed by 20 min recovery, and overexpression of RNAseH1 or depletion of GATAD2B and MBD3. IR = 5 Gy. Left: representative confocal microscopy images from three independent replicates; right: quantification of left, error bar = mean ± SEM, significance was determined using nonparametric Mann–Whitney test. $^{****}P \le 0.0001$. Scale bar = 10 μm, $n > 50$ cells from three biological repeats. (**B**) Bar chart showing pan-acetyl H4 (left) and histone H4 (right) ChIP levels at indicated sites (related to Fig. 4E) next to AsiSI cut in cells with or without 4OHT and depletion of GATAD2B, error bar = mean ± SEM, significance was determined using nonparametric Mann–Whitney test. $^{***}P \le 0.001$, $^{**}P \le 0.01$, n.s. not significant, $n = 3$. (**C**) Bar chart showing pan-acetyl H4 (left) and histone H4 (right) ChIP levels at indicated sites (related to Fig. 4F) next to AsiSI cut in cells with or without 4OHT and depletion of GATAD2B, error bar = mean ± SEM, significance was determined using nonparametric Mann–Whitney test. $^{***}P \le 0.001$, $^{**}P \le 0.01$, n.s. not significant, $n = 3$. (**D**) Western blot showing effect of knockdown of GATAD2B on histone H4 acetylation levels, NC, negative control. Histone H4 was used as loading control.

© The Author(s)

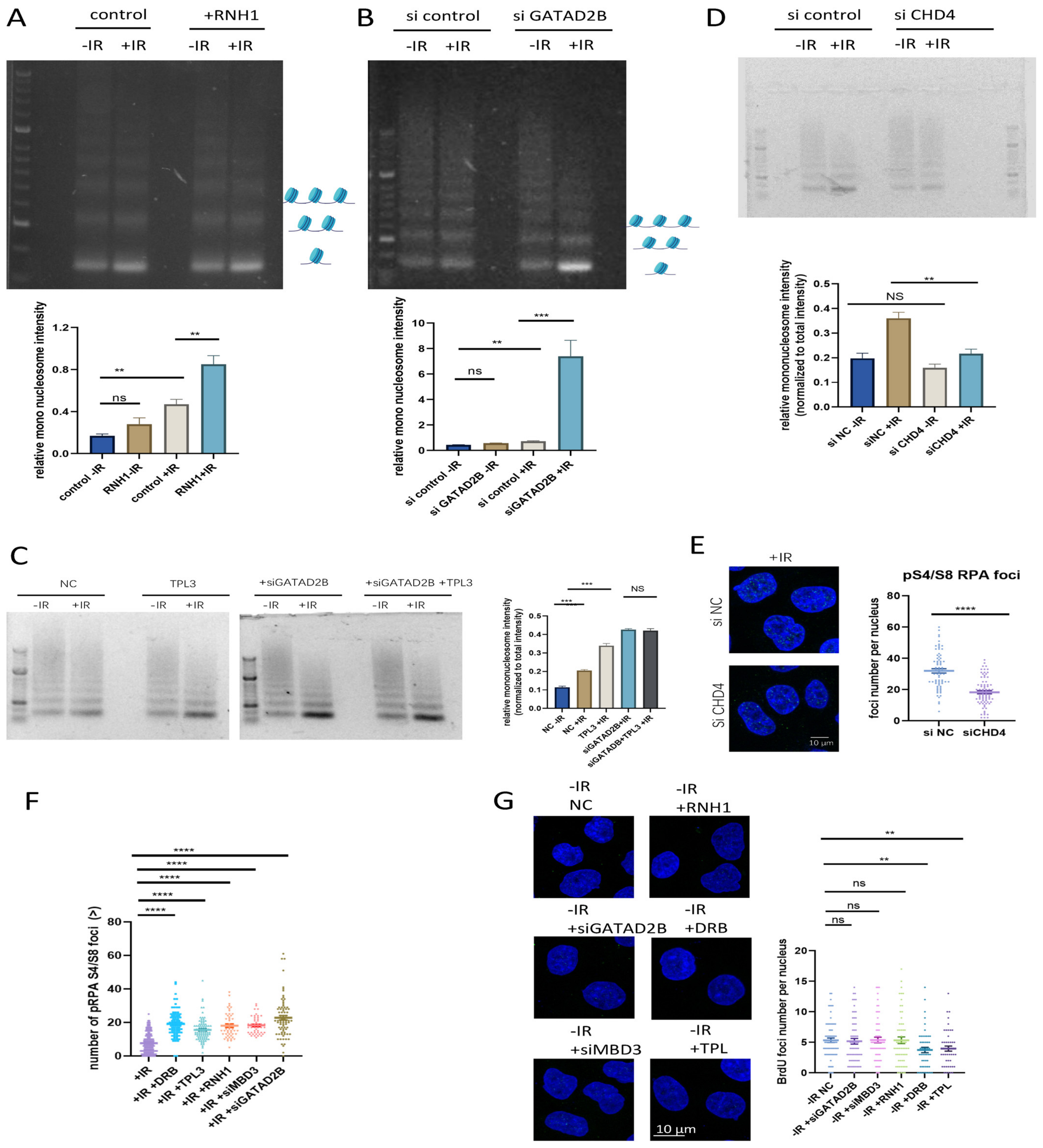

A
control
+RNH1
-IR +IR -IR +IR
relative mono nucleosome intensity
control -IR
RNH1-IR
control +IR
RNH1+IR
B
si control
si GATAD2B
-IR +IR -IR +IR
si control -IR
si GATAD2B -IR
si control +IR
siGATAD2B +IR
D
si control
si CHD4
relative mononucleosome intensity (normalized to total intensity)
si NC -IR
siNC +IR
si CHD4 -IR
siCHD4 +IR
C
NC
TPL3
+siGATAD2B
+siGATAD2B +TPL3
E
+IR
si NC
Si CHD4
pS4/S8 RPA foci
foci number per nucleus
si NC
siCHD4
F
number of pRPA S4/S8 foci (>)
+IR
+IR +DRB
+IR +TPL3
+IR +RNH1
+IR +siMBD3
+IR +siGATAD2B
G
-IR NC
-IR +RNH1
-IR +siGATAD2B
-IR +DRB
-IR +siMBD3
-IR +TPL
10 μm
BrdU foci number per nucleus
-IR NC
-IR +siGATAD2B
-IR +siMBD3
-IR +RNH1
-IR +DRB
-IR +TPL


 © The Author(s)

**Figure EV5. The GATAD2B-NuRD complex prevents chromatin hyper-relaxation.**

(**A**) Top: Representative DNA gel from three independent replicates showing nucleosome profile of wt and cells overexpressing RNaseH, with or without 5 Gy IR followed by 15 min recovery, after MNase treatment and DNA extraction. Bottom: Bar chart showing relative mononucleosome intensity of each lane. Significance was determined by Student *t* test: $^{**}P \leq 0.01$, n.s. not significant, error bar = mean ± SEM. (**B**) Top: Representative DNA gel from three independent replicates showing nucleosome profile of wt and cells depleted of GATAD2B, with or without 5 Gy IR followed by 15 min recovery, after MNase treatment and DNA extraction. Bottom: Bar chart showing relative mononucleosome intensity of each lane. Significance was determined by Student *t* test: $^{***}P \leq 0.001$, $^{**}P \leq 0.01$, n.s. not significant, error bar = mean ± SEM. (**C**) Left: Representative DNA gel from three independent replicates showing nucleosome profile of wt and cells depleted of GATAD2B, with or without triptolide treatment and in combination, with or without 5 Gy IR followed by 15 min recovery, after MNase treatment and DNA extraction. Right: Bar chart showing relative mononucleosome intensity of each lane. Significance was determined by Student *t* test: $^{***}P \leq 0.001$, n.s. not significant, error bar = mean ± SEM. (**D**) Top: Representative DNA gel from three independent replicates showing nucleosome profile of wt and cells depleted of CHD4, with or without 5 Gy IR followed by 30 min recovery, after MNase treatment and DNA extraction. Bottom: Bar chart showing relative mononucleosome intensity of each lane. Significance was determined by Student *t* test: $^{**}P \leq 0.01$, n.s. not significant, error bar = mean ± SEM. (**E**) Left: representative confocal images showing immunofluorescence signals of phospho-S4/S8 RPA32 in cells treated with 5 Gy IR followed by 2 h recovery with knockdown of CHD4. Right: quantification of left, error bar = mean ± SEM, significance was determined using nonparametric Mann–Whitney test. $^{****}P \leq 0.0001$. Scale bar = 10 μm, $n > 50$ cells from three biological repeats. (**F**) Analysis and quantification of larger phospho-S4/S8 RPA32 foci, as defined by defined by a prominence threshold of 2500 in the Find Maxima tool, in cells treated with 5 Gy IR followed by 2 h recovery with overexpression of RNAseH1 or transcription inhibition (TLP3 or DRB) or depletion of GATAD2B and MBD3. Error bar = mean ± SEM, significance was determined using nonparametric Mann–Whitney test. $^{****}P \leq 0.0001$, Scale bar = 10 μm, $n > 50$ cells from three biological repeats. (**G**) Representative confocal images from three independent replicates showing immunofluorescence signals of BrdU in cells upon no damage conditions with transcription inhibition (TLP3 or DRB), overexpression of RNAseH1 or depletion of GATAD2B and MBD3. Right: quantification of left, error bar = mean ± SEM, significance was determined using nonparametric Mann–Whitney test. $^{**}P \leq 0.01$, ns, no significance. Scale bar = 10 μm, $n > 50$ cells from three biological repeats.

© The Author(s)
